# Sequence, Structure and Ligand Binding Evolution of Rhodopsin-Like G Protein-Coupled Receptors: A Crystal Structure-Based Phylogenetic Analysis

**DOI:** 10.1371/journal.pone.0123533

**Published:** 2015-04-16

**Authors:** Steffen Wolf, Stefan Grünewald

**Affiliations:** 1 Department of Biophysics, CAS-MPG Partner Institute for Computational Biology, Key Laboratory of Computational Biology, Shanghai Institutes for Biological Sciences, Chinese Academy of Sciences, Shanghai, P. R. China; 2 Department of Biophysics, Ruhr-University Bochum, Bochum, Germany; 3 CAS-MPG Partner Institute for Computational Biology, Key Laboratory of Computational Biology, Shanghai Institutes for Biological Sciences, Chinese Academy of Sciences, Shanghai, P. R. China; Okayama University, JAPAN

## Abstract

G protein-coupled receptors (GPCRs) form the largest family of membrane receptors in the human genome. Advances in membrane protein crystallization so far resulted in the determination of 24 receptors available as high-resolution atomic structures. We performed the first phylogenetic analysis of GPCRs based on the available set of GPCR structures. We present a new phylogenetic tree of known human rhodopsin-like GPCR sequences based on this structure set. We can distinguish the three separate classes of small-ligand binding GPCRs, peptide binding GPCRs, and olfactory receptors. Analyzing different structural subdomains, we found that small molecule binding receptors most likely have evolved from peptide receptor precursors, with a rhodopsin/S1PR1 ancestor, most likely an ancestral opsin, forming the link between both classes. A light-activated receptor therefore seems to be the origin of the small molecule hormone receptors of the central nervous system. We find hints for a common evolutionary path of both ligand binding site and central sodium/water binding site. Surprisingly, opioid receptors exhibit both a binding cavity and a central sodium/water binding site similar to the one of biogenic amine receptors instead of peptide receptors, making them seemingly prone to bind small molecule ligands, e.g. opiates. Our results give new insights into the relationship and the pharmacological properties of rhodopsin-like GPCRs.

## Introduction

G-protein coupled receptors (GPCRs) form the largest group of membrane receptors [1]. Though they exhibit an extraordinary broad scope of recognized stimuli such as peptides, small organic molecules, calcium ions, and even light, all receptors share a heptahelical transmembrane (7TM) motif. Though GPCRs are the target of about 40% of all drugs commercially available [2], the first GPCR structure became available as late as 2000 with the successful crystallization of bovine rhodopsin [3]. Due to the development of new crystallization techniques, GPCR structure determination then had its major breakthrough in 2007 with the crystallization of the β_2_-adrenergic receptor (ADBR2) [4,5]. So far, 24 receptors have been structurally determined by x-ray protein crystallization [3–28].

In this work, we assess how far this subset of 7TM domain structures can give insight into the evolutionary relationship of the full set of known human GPCRs, with a special focus on rhodopsin-like GPCRs. The phylogeny of GPCRs was first studied based on a set of known receptor sequences [29]. The commonly used systematical classification system, the GRAFS-system by Fredriksson et al. [30], was developed from the data of the human genome project. In this system, GPCRs form the five distinct families of glutamate (G), rhodopsin-like (R), adhesion (A), frizzled/taste (F) and secretin (S) receptors. 90% of the receptors are found in the rhodopsin-like family, which is subdivided into the classes α–δ. However, GPCR phylogeny still is a highly debated field, as the receptors exhibit signs of phylogenetic mosaicism [31], and have developed under multiple overlapping evolutionary pathways [32]. 20 of the crystallized GPCRs belong to the rhodopsin-like class [3–22,26,27]. Furthermore, four receptor structures of non-rhodopsin GPCRs are available as structural models, of which two receptors are from the secretin family [23,25], one receptor from the frizzled/taste family [24], and one from the metabotropic glutamate receptor family [28]. Due to this availability of both rhodopsin-like and non-rhodopsin-like GPCR structures, we are now able to revise this classification based on structural information. While evolution of proteins so far is mostly discussed on a sequence comparison basis, paleontology deduces evolutionary sequences from comparison of structural features of species. With the now available set of GPCR structures, we here attempt to assess the evolution of GPCRs by combining both of these two possible approaches. We used the three non-rhodopsin GPCR structures to find the branching point between non-rhodopsin and rhodopsin-like GPCRs.

A better understanding of the underlying phylogeny can help in the deorphanization of GPCRs, whose ligands are still unknown, and therefore propose to be new druggable targets for pharmaceutical research [33]. Furthermore, we can detect new relations between GPCRs with this understanding, and thus find new insight into their pharmacological properties. The available set of GPCR structures now enables us to make an exact determination of sequence parts belonging to different structural subdomains, which is more precise than results from secondary structure prediction. By restricting our analysis to the 7TM domain, and ligand binding residues, we can gain precise information about the evolutionary development of these structural subfeatures. Especially for comparing ligand-binding residues, it is essential that we now can make a direct match and comparison of residues forming these sites in different structures, and thereby overcome biases introduced by sequence alignment algorithms. We first performed this analysis with the sequences of crystallized GPCRs transmembrane helices together with their loop domains. Here we found that the connection point between rhodopsin-like and non-rhodopsin-like GPCRs is found between peptide-binding receptors and small molecule receptors, close to the position of rhodopsin. We analyzed how this tree-like evolution corresponds to the changes of structural features found within the existing GPCR crystal structures. The development of small molecule receptors seems to have started with a hydrophobic ligand-recognizing receptor, most likely an opsin precursor, by an interplay of small molecule ligands and the opsin extracellular loop 2 (el2). Last, in an analysis of ligand-binding and sodium-binding [34,35] cavity forming residues, opioid and biogenic amine receptors exhibit signs of homoplasy, possibly explaining the affinity of opioid receptors for opiates despite being peptide receptors.

## Materials and Methods

### Phylogenetic analysis of GPCR structures

For the structural analysis, we considered the human sequences of a set of 24 crystallized GPCRs [3–28]. In case that several crystal structures were available for one receptor, we chose the one with highest available resolution for data analysis [respective PDB IDs: rhodopsin (OPSD): 1U19 [36]; β_2_ adrenergic receptor (ADBR2): 2RH1 [37]; β_1_ adrenergic receptor (ADBR1): 2YCW [6]; A_2A_ adenosine receptor (AA2AR): 4EIY [20]; D3 dopamine receptor (D3DR): 3PBL [8]; H1 histamine receptor (H1HR): 3RZE [10]; (S1PR1): 3V2Y [11]; chemokine receptor CXCR4 (CXCR4): 3OE0 [9]; chemokine receptor CCR5 (CCR5): 4MBS [26]; δ-opioid receptor (OPRD): 4EJ4 [17]; μ-opioid (OPRM): 4DKL [14]; κ-opioid (OPRK): 4DJH [15]; N/OFQ opioid (OPRX): 4EA3 [16]; M2 muscarinic receptor (ACM2): 3UON [12]; M3 muscarinic receptor (ACM3): 4DAJ [13],; neurotensin receptor 1 (NTR1): 4GRV [18]; protease-activated receptor 1 (PAR1): 3VW7 [19]; serotonine receptor 1B (5HT1B): 4IAR [21]; serotonine receptor 2B (5HT2B): 4IB4 [21]; purine receptor P2Y12 (P2Y12): 2PXZ [27]; glucagon receptor (GLR): 4L6R [23]; smoothened receptor (SMO): 4JKV [24]; corticotropin-releasing factor receptor 1 (CRFR1): 4K5Y [25]; metabotropic glutamate receptor 1 (GRM1): 4OR2 [28]]. All structures were loaded into PyMol [38], a standard protein structure visualizer, for structural analysis and structure/sequence comparison. In case that a GPCR structure was obtained from a non-human or mutation-stabilized receptor, the respective native human sequence was used for the following sequence comparisons. Structure/sequence comparison was performed directly in PyMol. Rhodopsin-like GPCR helices were aligned according to the most conserved residues from Ballesteros-Weinstein numbering [39]. If this was not applicable (helix V in S1PR1 and P2Y12), we additionally focused on overlaying the sequences to keep Tyr5.58 conserved [40,41]. For the rhodopsin- and non-rhodopsin-like GPCR alignment, we focused on finding a best fit for the full heptahelical helix Cα atom arrangement with the “align” function of PyMol, followed by an individual fit of each transmembrane helix. To additionally take into account the loop domains in this evaluation, we extracted the respective sequences of extra- and intracellular loops 1–3, and aligned each loop of the 24 receptors separately. The resulting aligned loops were then introduced into the structural alignment between the respective helices. The resulting alignment is attached in FASTA format as S1 File. Phylogenetic trees were calculated with PhyML 3.0 [42], using JTT as substitution model. PhyML is a tool for the fast calculation of computationally expensive maximum likelihood [43] phylogenetic trees, and allows different choices of substitution models and tree rearrangement methods (Nearest Neighbor Interchange [NNI] and Subtree Pruning and Regrafting [SPR]). Dendroscope3 [44] was used for tree visualization. Dendroscope is a program for working with rooted phylogenetic trees and networks. It provides a number of methods for drawing and comparing rooted phylogenetic networks, and for computing them from rooted trees. The respective network analysis and visualization was performed using SplitsTree4 [45], using maximum likelihood distances and JTT [46] as substitution model to calculate NeighborNet networks. NeighborNet is a distance-based method to construct circular splits networks [45]. In short, a splits graph is an assembly of several possible evolutionary trees for a given sequence set. Thus it can display multiple possible evolutionary relationships at once, which is necessary to resolve signals of lateral gene transfer, recombination events, mosaicism, and homologous evolution [45]. In contrast to this, the calculation of a maximum likelihood tree only gives the tree-like relationship that is most probable under the assumed model of sequence evolution. If the input distances from the sequence set correspond to a perfect phylogeny, then the network will only contain the splits (“branches”) of the most probable tree, and network and tree analysis will give the same result. SplitsTree4 [45] is an interactive and comprehensive tool for calculating different types of phylogenetic networks from sequences, distances, and trees. We chose the JTT model, as it was the only method implemented in SplitsTree4 capable to calculate maximum likelihood NeighborNets across the full range of sequence lengths we employed. For ligand binding pocket sequences, we calculated a network with uncorrected P distances, as some of the sequences were too dissimilar to compute meaningful maximum likelihood distances. Evolutionary Trace (ET) calculations were performed with the Baylor code [47,48] using the BLOSUM62 matrix [49] and default parameters. ET assesses the evolutionary relationship of protein sequences by calculating a sequence similarity-based phylogenic tree. Secondary structure predictions were performed with Psipred [50,51]. Pispred takes the profile output of PSI-BLAST [52] and takes a consensus secondary structure prediction from four independently trained sets of neural networks. All molecular figures were prepared with PyMol [38].

### Phylogenetic analysis of full human GPCR repertoire

For the full non-rhodopsin GPCR set and the rhodopsin-like GPCR set, GPCR amino acid sequences were obtained from the Uniprot database [53] and are named accordingly. From this set of 828 reviewed Uniprot GPCR sequence entries (status of March 21^st^ 2012), we removed the sequences of GPR98, GP112, GP179, CELR1, CELR2, and CELR3, as their length (>1500 amino acids) prolonged the phylogenetic calculations to an unreasonable extend (no completion after 8 days). The sequence set used for alignment and phylogenetic analysis thus contains 822 entries. Sequence alignments were performed using MUSCLE 3.8 [54] with default parameters. MUSCLE is a fast and efficient algorithm for multiple sequence alignment procedures, which includes fast distance estimation using k-mer counting, progressive alignment using a profile function called the “log-expectation” score, and refinement using tree-dependent restricted partitioning. Phylogenetic analysis was performed with PhyML 3.0 [42]. A PhyML analysis of the full GPCR set failed, probably due to too much sequence variation. We therefore developed a hierarchical approach to determine the best fitting crystal structures for 7TM sequence determination. For this, we added the sequences of the 24 structurally known GPCRs [3–17,19,21–25,27,28,38,55] in their full length sequence to the set. Structural analysis for 7TM determination was performed with PyMol [38]. After an initial sequence alignment of the full set with MUSCLE, we constructed an initial Neighbor Joining tree using the BLOSUM62 matrix (calculations were done with Jalview [56,57], a graphical user interface for sequence comparison). We then extracted the leaf with the longest distance from the leaves containing the crystal structure sequences, and repeated sequence alignment and leaf extraction. We performed this approach iteratively, extracting seven leaves (groups 1–7), until the GPCR structural sequences were distributed over the tree of the remaining sequences. The remaining leaves were separated so that each of them contains a minimum of one GPCR structure sequence. Leftover sequences were collected in additional groups. A detailed list of the members of each group is given in S2 File. To all sequence set groups, the GPCR structure sequence set was added. All groups were then separately subjected to a MUSCLE sequence alignment and Neighbor Joining tree construction. For each group, the GPCR structure sequence, which was closest to the majority of sequences, was chosen for the determination of the 7TM helices. In case of ambiguity, the group was iteratively subdivided, realigned, and appearing leaves extracted along with their closest structurally available receptor sequence. The detailed group/structure combinations can be found in S2 File. The 7TM positions of the respective set were then determined from the respective template GPCR structure, and the sequences cut down to the presumed 7TM domain (i.e. transmembrane helices I-VII together with interconnecting loops). All 7TM sequence groups were then reunited into two large sequence sets: the resulting groups 1 to 6b, 8a(c), and vomeronasal receptors in 9a were found to be non-rhodopsin-like receptors, and thus merged into the non-rhodopsin group. The remaining groups (6c to 11c) were merged into the rhodopsin-like group. To find the connecting node between rhodopsin-like and non-rhodopsin-like receptors, the 7TM domain sequences of the four crystallized non-rhodopsin-like GPCRs were added to the rhodopsin-like GPCR set, and the 7TM domain sequences of the rhodopsin-like GPCRs to the non-rhodopsin-like GPCR set, vice versa. With these sets, one final MUSCLE alignment each was performed followed by a PhyML maximum likelihood analysis. The maximum likelihood analysis was performed with an initial Neighbor Joining tree, and JTT as amino acid substitution model. Tree topology, branch length, and rate parameters were optimized. Topology optimization was performed starting with a NNI, or an SPR tree topology search, respectively, followed by a second optimization with the respective other method. To assess the robustness of the resulting tree, we carried out a bootstrap analysis with 528 replicas. The trees can be found in Newick format in S3 and S4 Files, and the respective alignments in FASTA format in S5 and S6 Files. We here display the trees with the highest likelihood from both optimization runs. In the rhodopsin-like GPCR tree, we removed SMO, GP141, GP143, and GP148 from display, as they appeared unreasonably far from the remaining sequences, and thus seem not to be correctly placed within the tree. For finding the connection to the non-rhodopsin GPCRs, we therefore relied on the respective positions of GRM1, GLR, and CRFR1.

## Results and Discussion

### Phylogenetic analysis of crystal structure subset of human GPCRs

**Fig 1:**
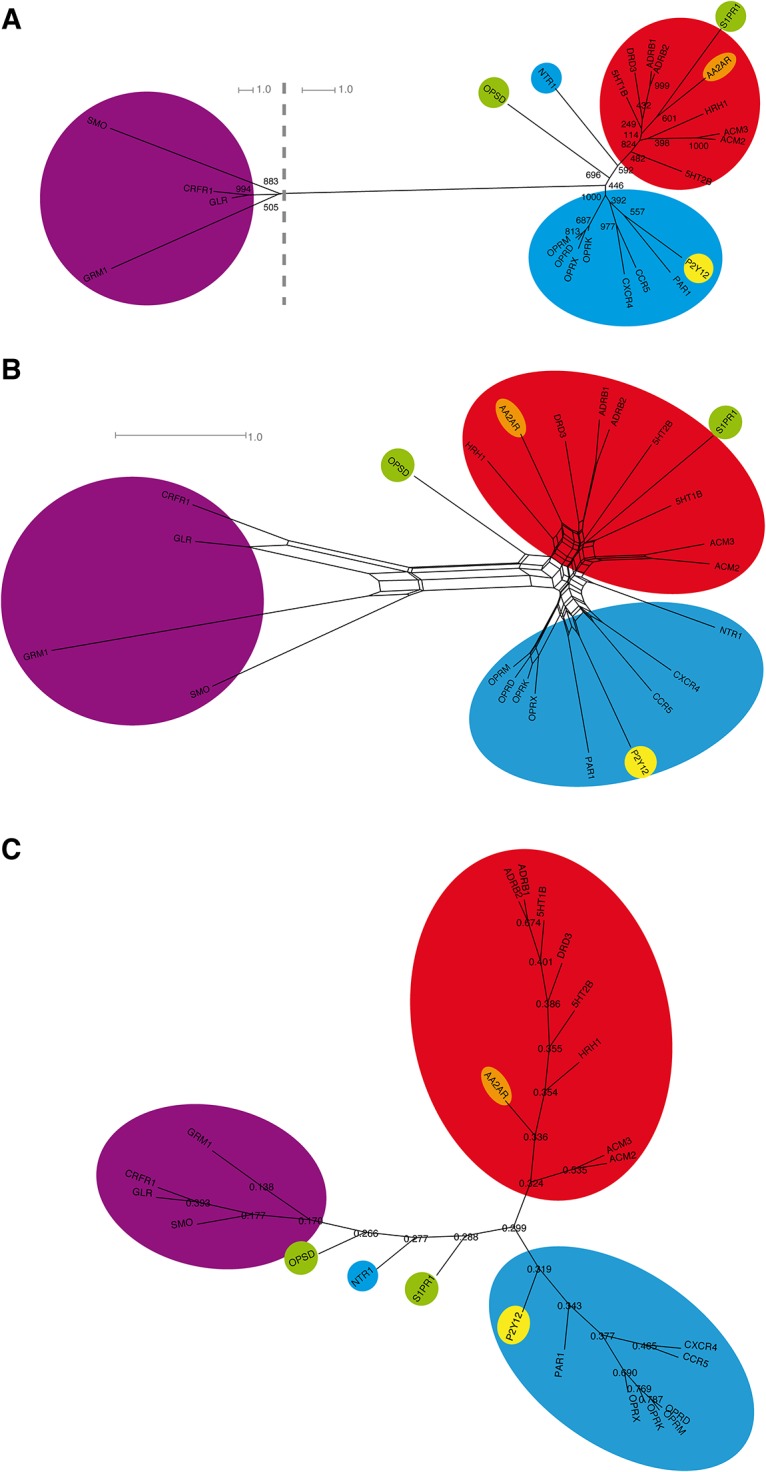
Phylogenetic analysis of the GPCR crystal structure set. Maximum likelihood tree (A) analysis, maximum likelihood distance-based NeighborNet (B), and Evolutionary Trace sequence similarity-based tree (C) of the 7TM domain of crystallized receptors [3–28]. Naming according to the Uniprot database [53]. Receptors with hydrophobic residues highlighted in green, adenosine receptors in orange, biogenic amine receptors in red, non-rhodopsin-like receptors in purple, peptide-binding receptors in cyan, purine receptors in yellow. Numbers in (A) denote how often the respective node was found in bootstrap replicas (1000 samples in total). Numbers in (C) denote the ratio of sequence similarity between both sequence groups forming the respective node. The two tree analyses show a separation of rhodopsin-like GPCRs into small molecule receptors, i.e. amine receptors, on one side, and peptide/purine receptors on the other side, with rhodopsin (OPSD) being in between both subgroups. The connection with the non-rhodopsin-like GPCRs is formed in the middle between both branches, too, and is positioned close to rhodopsin and neurotensin receptor 1 (NTR1). The network and both tree analyses all show a good topological agreement, indicating a general tree-like evolution signal. However, the network exhibits large meshes around the connection point of non-rhodopsin-like and rhodopsin-like GPCRs, indicating a certain ambiguity about their precise connection.

Fig 1 shows a maximum likelihood tree (A) and a maximum likelihood distance-based NeighborNet [58] (B) of the 7TM domain of the 24 crystallized receptors. The sequences of the 7TM helices were aligned according to their structural best fit, and the connecting loops were subjected to a sequence alignment with MUSCLE [54]. We added the helix-connecting loops to the 7TM helices, as a focus on solely the helices would introduce a gap into the protein sequence, and thus create artifacts in the calculated evolutionary trees. The overall bootstrap values of the tree are reasonably high (>400) and show a good overall robustness of the tree, with the exception of the 5HT1B (249) and AA2AR / S1PR1 branching nodes (114). The tree analysis shows a separation of rhodopsin-like GPCRs into small molecule receptors, i.e. amine receptors, AA2AR, and S1PR1, on one side, and peptide/purine receptors on the other side. Rhodopsin (OPSD) branches off between both subgroups. The connection with the non-rhodopsin-like GPCRs is formed in the middle between both branches, too, and is positioned close to rhodopsin and neurotensin receptor 1 (NTR1). With a bootstrap value of 446 out of 1000, we hold this connection point to be reasonably robust. The relative positions of the rhodopsin-like GPCRs are in qualitative agreement with the earlier analysis of Fredriksson et al. [30], while the connection node with non-rhodopsin-like GPCRs is at a previously not reported position. To counter-check the robustness of the tree topology, we performed a NeighborNet analysis. The network and the tree analysis both show a good topological agreement, indicating a general tree-like evolution signal. However, the network exhibits large meshes around the connection point of non-rhodopsin-like and rhodopsin-like GPCRs, indicating a certain ambiguity of their connection node. Furthermore, NTR1 is found at a position with an equal distance towards both the muscarinic acetylcholine receptor 2 (ACM2) and the chemokine receptor CXCR4, and further away from rhodopsin than seen in the tree analysis. S1PR1 and AA2AR, too, differ in their network positions from the ones in the tree analysis. We assume that these differences derive from the relatively small number of protein sequences used in the alignment.

To further evaluate the robustness of our maximum likelihood-based tree, we calculated a Evolutionary Trace (ET) sequence similarity tree [47,48] from our crystal structure-based alignment. The ET method was already successfully applied to find evolutionary important amino acids in biogenic amine receptors [59,60]. Fig 1C shows the resulting sequence similarity-based tree. The ET tree topology is in good agreement with our maximum likelihood tree in Fig 1A: non-rhodopsin receptors, biogenic amine receptors and peptide receptors are well separated, and rhodopsin / NTR1 are found between the three groups. A difference is found for the placement of S1PR1, which is now close to rhodopsin / NTR1. Due to this nice overall agreement of our maximum likelihood tree and the ET tree, we hold the observed subgroup connection and our tree topology to be robust.

Based on these results, it seems that in the subset of crystallized rhodopsin-like GPCRs, two main evolution branches exist: peptide-binding GPCRs and small molecule binding GPCRs. The development observed in the subset implies that small molecule binders seem to have evolved via hydrophobic ligand-binding GPCR precursors. The hydrophobic ligand binders themselves later developed into opsines and sphingosine receptors. An exception from this rule is the purine receptor P2Y12, which seems to have developed out of peptide receptors, too. This is in agreement with the observation that the P2Y12 crystal structure is closer to the structure of the protease-activated receptor PAR1 receptor than to ADRB2 [27]. However, as stated above, the small structure-based sequence set seems to contain artificial positions of NTR1, AA2AR, and S1PR1. To remove these artifacts, we extended our results from crystal structures to the full scope of known human GPCR protein sequences. For this, we used the available crystal structures as templates to determine the 7TM motifs of the GPCRs without available structural model.

We initially set out to assess the evolution of the full scope of the human GPCR repertoire with our structure-based approach. Our analysis reveals the same subgroups like observed by Fredriksson et al. [30], while it additionally includes vomeronasal receptors and several orphan receptor groups. However, as S1 Fig shows, while some subfamilies seem to be well resolved (glutamate receptors, frizzled/smoothened receptors, secretin receptors, taste receptors type 2, vomeronasal receptors, adhesion receptors), others lack a clear separation from the tree basis (orphan families) or are inexplicably separated over different nodes (taste receptors type I, EGF-like receptors). Our analysis thus cannot give a clear picture of the evolution of non-rhodopsin-like receptors yet. We assume that the currently available four non-rhodopsin GPCR structures do not sufficiently cover the full sequence range for a phylogenetic analysis. In the following, we therefore here focus on the analysis of the rhodopsin-like GPCR family of Fredriksson et al. [30]

### Phylogenetic analysis of structure-based alignment reveals new ordering of human rhodopsin-like GPCR

**Fig 2:**
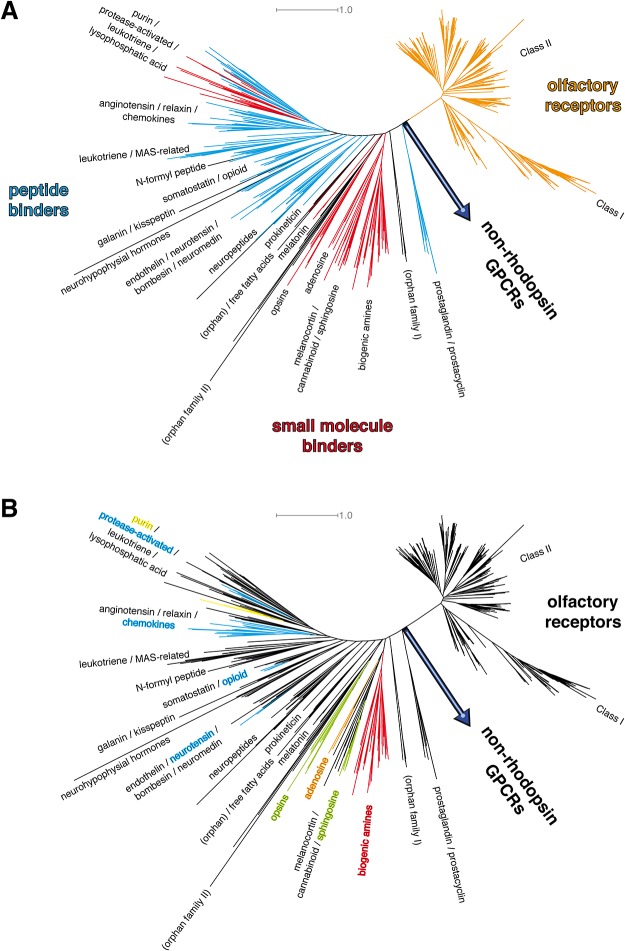
Phylogenetic analysis of the full rhodopsin-like GPCR crystal structure set. Phylogenetic analysis of known human rhodopsin-like GPCR protein 7TM sequences after assignment of the transmembrane helices by alignment of the full scope of human GPCR protein sequences with the respective 7TM sequences from receptors with available x-ray structures. A: classification according to known ligand binding. The tree shows three major branches: olfactory receptors (in orange), small molecule binders (red), peptide binders (cyan). Orphan receptors in black. Olfactory receptors form a subclass of their own within the rhodopsin-like class. Small molecule binding GPCRs form a clear cluster. Peptide receptors cluster at the left end of the tree, starting with the neuropeptides subbranch. A 2^nd^ set of small ligand receptors, which are the purine, leukotriene, and the free fatty acids receptors, mixes with protease-activated receptors. B: classification according to ligand properties as found in X-ray crystallography structures. Color scheme like in Fig 1. Known crystallized ligands can be subdivided into hydrophobic ligands (green), biogenic amines (red), hydrophilic ligands (orange), peptides (cyan), and purines (yellow). The small molecule binding receptor families closest to the common node with non-rhodopsin GPCRs are hydrophobic ligand GPCRs. The overall picture is in good agreement with the one found for the crystallized GPCRs from Fig 1.

To extend our crystal structure-based phylogenetic tree presented in Fig 1A, and remove artifacts from a too small number of sequences, we expand our investigation to the full scope of human rhodopsin-like GPCR sequences. As can be seen in Fig 2A, the newly calculated tree topology is in good agreement with the crystal structure-based tree in Fig 1A. It shows three major branches for the rhodopsin-like GPCR: olfactory receptors, small molecule binders, and peptide binders. The two different mammalian olfactory receptor classes (termed I and II) [61] are well resolved. Surprisingly, they are found apart from the other small molecule binding GPCRs, suggesting that they branched off at an early state of development of rhodopsin-like GPCRs [62,63]. Though no olfactory GPCR structure has been solved yet, we are convinced that our suggested position of this family is correct, as we used the rhodopsin structure to assign helix positions, which proved to be a good template for the homology modeling of olfactory receptors [64]. Furthermore, a new crystal structure of opsin in an active state bound to a lipid molecule suggest a close structural connection between opsin and olfactory receptors [65]. While the general topology of our tree is in overall agreement with the one from Fredriksson et al. [30], the position of the branching point between olfactory receptors, non-rhodopsin GPCRs, and the remaining rhodopsin-like GPCRs is a new finding and the most pronounced difference to this classification.

Fig 2B shows the new tree resolved by ligand properties: The small molecule binding receptors branching off closest to the common node with non-rhodopsin GPCRs are hydrophobic ligand binders, here sphingosine receptors and opsins, together with adenosine receptors. This suggest that their ancestors form the earliest small molecule binding GPCRs. Peptide receptors can be found before (prostaglandin & prostacyclin receptors) and after (e.g. opioid receptors, chemokine receptors) the branching nodes of the hydrophobic ligand receptors. Consequently, the ancestral rhodopsin-like GPCRs seem to have been peptide receptors, which is in agreement with the findings of Pele et al. [32]. This means that while opioid and chemokine receptors retained the property to bind and recognize peptide ligands, as most non-rhodopsin GPCRs do, some structural change must have led to the development of small molecule binding receptors, and this change must have happened with the development of an opsin / sphingosin receptor precursor. A similar structural change must have happened twice, as purine / leukotriene / free fatty acid receptors seem to have evolved out of a protease-activated receptor precursor. This suggests a possible easy interchangeability of peptide and purine binding, which is in agreement with the assumption that GPCRs have evolved from cAMP receptors [63], cAMP receptors essentially are purine receptors, too, but are absent in the human genome. In addition, the ligand property-focussed tree in Fig 2B allows for a tentative characterization of ligand properties of orphan receptors present in the analysis. For example, GPR161, which was recently found to be a new drug target for breast cancer treatment [66], is present in the orphan family II / opsin branch. Therefore, it most likely is recognizing hydrophobic small molecule ligands. However, we have to state clearly that the tree analysis of this large GPCR subset shows a high uncertainty, especially at the basis nodes (bootstrap values of 0 to 50 with 528 replicas). Therefore, we investigate in the following how far structural details of the available crystal structures support this tree.

### An opsin precursor forms the loophole in development from peptide to small molecule GPCRs

**Fig 3:**
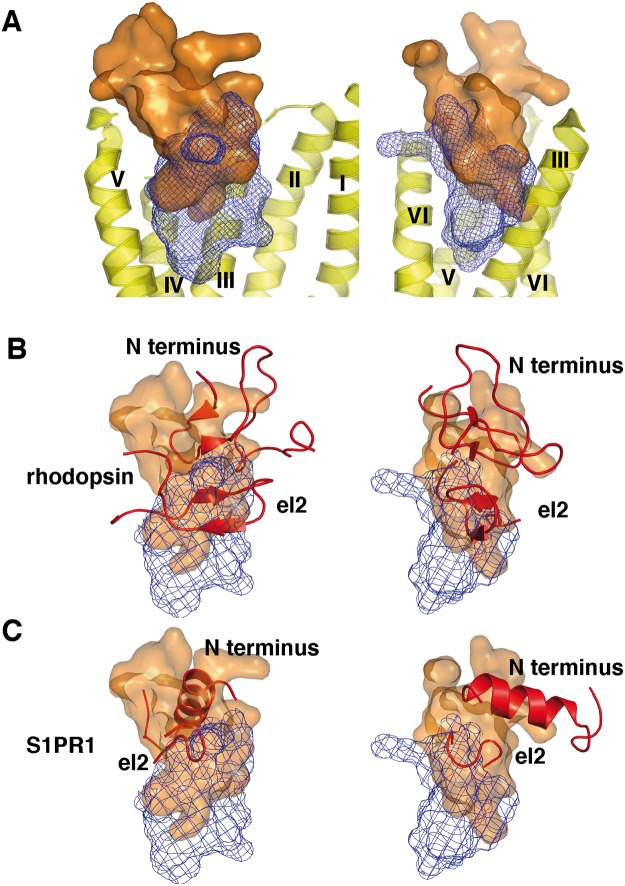
Consensus binding volume of peptide and small molecule ligands. A: Volume accessed by peptide ligands [9,18] and small organic ligands [7,8,11–13,21,22,36,37] as observed in rhodopsin-like GPCR crystal structures after superposition of their 7TM helices. Left and right images are views rotated by 90 degrees. Peptide ligand volume as orange surface, small organic ligand volume as blue mesh. Helices from rhodopsin (PDB ID 1U19) [36] displayed in yellow as optical reference. Both types of ligands bind to the targeting receptors between helices II-VII. While peptide ligands bind around the height of the extracellular end of the helical bundle, small molecule ligands bind in a volume within the extracellular half of the helical bundle. Both volumes share a common region. B, C: Overlay of small ligand accessed volume (blue mesh) and peptide ligand accessed volume (orange surface) with N termini and el2 from rhodopsin (B) and S1PR1 (C). In rhodopsin, the N terminus and el2 fill out the peptide binding volume, while el2 shows a good match with the region claimed by both peptide binding volume and small ligand binding volume. In S1PR1, N terminus and el2 still both are positioned within the peptide binding volume. However, el2 is shortened compared to rhodopsin, and only partially fills the volume claimed by both peptide and small ligand binding.

We first evaluate if ligand binding in GPCRs exhibits conserved features, which we can relate to the protein structure. Fig 3 shows the volume occupied by peptide ligands in peptide-binding GPCRs (PDB IDs 3OE0 [9] and 4GRV [18]) and by organic ligands in small molecule-binding GPCRs (PDB IDs 2YCW [6], 3EML [7], 3PBL [8], 3RZE [10], 3V2Y [11], 3UON [12], 4DAJ [13], 4IAR [21], 4IB4 [21], 4NTY [27], 1U19 [36], and 2RH1 [37]) after superposition of their 7TM helices. Fig 3A displays the position of these volumes in respect to the 7TM bundle. Both peptide ligands and small molecule ligands bind to the targeting receptors between helices II-VII. While peptide ligands bind around the height of the extracellular end of the helical bundle, small molecule ligands bind in a volume within the extracellular half of the helical bundle. Both volumes share a common region, which interestingly is found in the volume occupied by the rhodopsin el2 (see Fig 3B). We cannot tell if this contact volume is important for receptor activation or protein stability, as active state or agonist-bound GPCR structures only exist for four receptors so far [67–72], which is not sufficient for such an analysis. In the following, we examine how the different receptor structures interact with these contact domains. For this, we set the respective structures into the evolutionary order proposed by our rhodopsin-like family tree in Fig 2.

**Fig 4:**
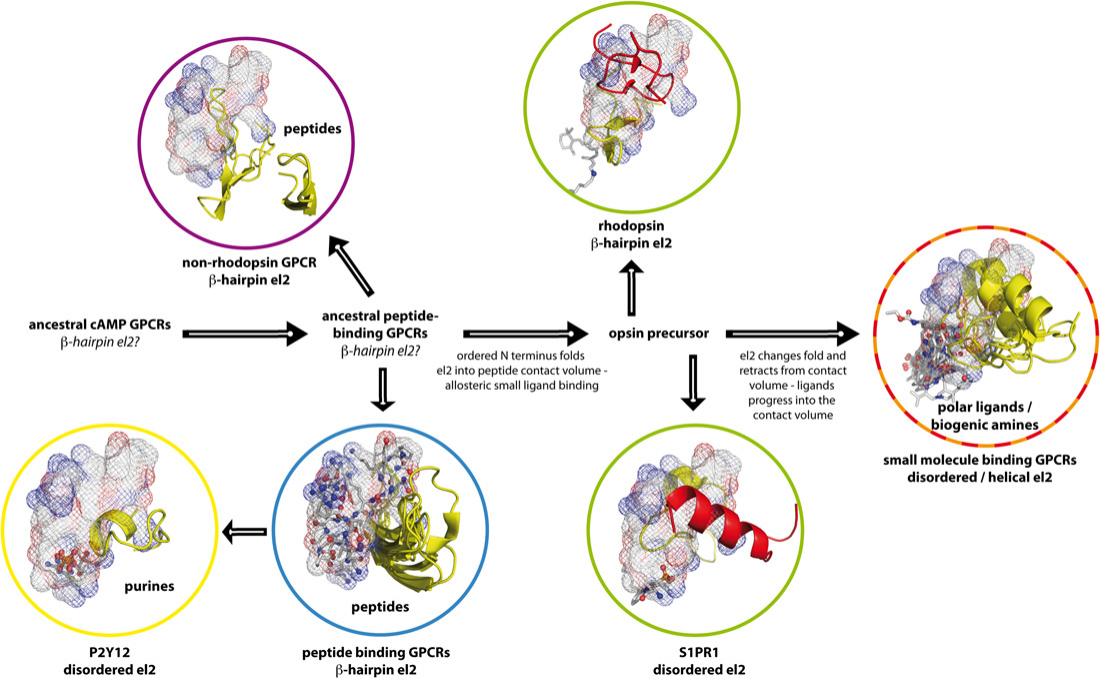
Development of peptide and small molecule binding GPCRs by protein self-interaction with el2. Peptide ligand binding volume in rhodopsin-like GPCRs (see Fig 3) as mesh, small organic ligands (see Fig 3) in grey sticks with polar oxygen / nitrogen atoms as spheres, el2 as yellow cartoon, N termini as red cartoon. In all known non-rhodopsin GPCR structures (purple circle) [23–25,28], el2 forms a β–hairpin structure, which surrounds the peptide binding volume. In rhodopsin-like peptide receptor structures with bound peptide ligand (blue circle) [9,18], el2 forms a β-hairpin, which flanks this domain, too. This common arrangement suggests that during evolution, both rhodopsin-like and non-rhodopsin peptide binders kept common ancestral peptide ligand binding features. Furthermore, both classes kept a common position and shape of el2. The length of the rhodopsin el2 is in good agreement with the one observed in peptide-binding GPCRs. In rhodopsin (green circle, top), el2 goes right through the common peptide / small molecule ligand volume, and is held there by steric constraints from the N terminus (see Fig 3B). Retinal binds underneath and outside of this contact domain at a position close to the protein center. In the sphingosine receptor (green circle, bottom), el2 is disordered and has contracted in comparison to rhodopsin, while still being in contact with the N terminus (compare Fig 3C). The ligand has advanced into the common ligand domain, performing contacts there. We therefore assume that an opsin precursor forms the link between peptide-binding GPCRs and small molecule-binding GPCRs. In small molecule-binding GPCRs (red / orange circle), el2 is disordered or helical and has retracted from the peptide-binding domain. Small molecule ligands bind in the peptide contact volume at the position of el2 in rhodopsin, and below it. It seems that the ancestral rhodopsin-like GPCRs initially possessed an el2 in the form of a β-hairpin. Peptide binders retained el2 in this form. Small molecule ligands for GPCRs seem to have then developed as allosteric binders via an opsin ancestor as key intermediate, with el2 substituting a bound peptide ligand. Along the proposed pathway, small molecule ligands then seem to have substituted el2, who retracted from the binding site and lost the β-hairpin conformation. Purine receptors seem to have undergone a similar development.

Fig 4 shows the development of GPCR structures into peptide and small molecule receptors proposed by our tree analysis. As stated above, cAMP receptors are the common ancestors of GPCRs (with the exception of glutamate receptors) [63]. From this, peptide-binding GPCRs seem to have evolved first [32]. The peptide binding volume, small organic molecule ligands, and el2 exhibit an interesting relationship along the presumed evolutionary pathway: in all known non-rhodopsin GPCR structures [23–25,28], which so far all represent peptide binders, el2 forms a β–hairpin structure, which surrounds the peptide binding volume of rhodopsin-like GPCRs. We here classify the crystallized glutamate receptor GRM1 as a peptide receptor, too, as glutamate binds within an extracellular protein domain, which transfers its conformational changes into the transmembrane domain. Thus, a protein/protein contact activates the transmembrane domain. In rhodopsin-like peptide receptor structures with bound peptide ligand [9,18], el2 is found in form of a β-hairpin, too, and also flanks the peptide binding volume. This common arrangement suggests a retention of both peptide ligand binding features and el2 loop form and position during peptide binding GPCR evolution. Confirming our assumption from the previous section, both rhodopsin-like and non-rhodopsin peptide binders must have evolved from a common precursor. In rhodopsin, el2 goes right through the peptide-binding domain, and is held there by steric constraints from the N terminus. Retinal binds underneath the contact domain at a position close to the protein center. The length of the rhodopsin el2 is in good agreement with the one observed in peptide binding GPCRs, and its position equals the position of the common peptide / small molecule ligand volume (see Fig 3B). In the sphingosine receptor structure (see Fig 3C), el2 is held in this dual binding volume due to contacts with the N terminus as well. However, el2 here is disordered and has partially retracted from the binding volume in comparison to rhodopsin. Like a compensation for this, the ligand has advanced into the peptide-binding domain, performing contacts there. In polar ligand and biogenic amine GPCRs, el2 is disordered or helical and has retracted from the peptide-binding domain. Small molecule ligands bind in the peptide contact volume at the position of el2 in rhodopsin, and below it.

Combining these findings with our phylogenic tree in Fig 2, we judge that the ancestral rhodopsin-like GPCRs initially possessed an el2 in the form of a β-hairpin. Peptide binders and rhodopsin retained el2 in this form. The first small molecule-binding GPCR seems to have been an opsin precursor. As can be seen in Fig 3B, both N terminus and el2 seem to substitute a bound orthosteric peptide ligand. This finding is particularly interesting, as ligand-free opsin at low pH adopts a conformation, which closely resembles the active meta II state [73] and is capable to bind and activate the rhodopsin-specific G-protein transducin [74]. In agreement with this, the position and conformation of el2 in the meta II—state rhodopsin [72,75], in retinal free [76] and G-protein mimic-bound opsin [74] is very similar, while the arrangement in inactive dark-state rhodopsin [36] differs from them (see S2 Fig). Furthermore, el2 structural changes are associated with formation of the meta II state [77]. Retinal itself does not bind within this peptide binding volume, but below it. This suggests that retinal is not a conventional small molecule ligand. Instead, it seems that the orthosteric binding site of the chromophore has developed from an allosteric binding site in an initially peptide-binding receptor. This peptide binder must have developed the capability to bind retinal covalently via the appearance of Lys296. This model is in agreement with the findings of Feuda et al. [78], and negates a direct evolutionary connection between microbial and animal rhodopsins [79]. In the sphingosine receptor, el2 has partially retracted from the peptide contact domain. Instead, the sphingosine ligand extends from the new small molecule-binding site into the peptide-binding domain, forming polar contacts there, and partially substitutes el2 (compare Fig 3C). This trend continues in biogenic amine receptors and the A_2A_ adenosine receptor, where el2 has completely retracted from the contact domain. As a compensation, small molecule ligands form contacts with the receptor in the new orthosteric small ligand binding site, and within the bottom of the peptide contact domain. We here need to point out that while we deduce this structural evolution from current receptors, the real development happened within ancestors of these receptors. Therefore, they all contain additional changes, e.g. the loss of the el1-el2 disulphide bond in S1PR1 [11], which we do not discuss here. Purine receptors seem to have undergone a similar, separate development from peptide receptors to small molecule receptors. However, as stated above, this might be a development back to cAMP receptors [63].

Concerning olfactory receptors, we currently cannot judge if their origin lies within an ancestral opsin as well, or if they followed a separate evolutionary pathway. On one hand, a development of olfactory receptors out of an opsin ancestor is a valid hypothesis: as stated before, rhodopsin is a suitable structural template for modeling olfactory receptors [64,65], which implies a high structural similarity. Both receptor subclasses bind hydrophobic ligands within their 7TM core. As we show in S3 Fig, el2 exhibits a quite conserved sequence and length (ca. 35 amino acids) in olfactory receptors, which is seven amino acids more than in the rhodopsin el2. There is no obvious sequence similarity between el2 in olfactory receptors and in rhodopsin. However, for the example of 20 randomly chosen olfactory receptors, a secondary structure prediction via Psipred [51] predicts predominantly sheet features for their el2 sequences. We therefore assume that like in rhodopsin, el2 in olfactory receptors forms a β-hairpin. On the other hand, several arguments exist for a separate evolution of small molecule binders and olfactory receptors: our phylogenetic analysis shows a maximum likelihood distance between opsins and olfactory receptors, which is considerably longer than the opsin / small molecule binder distance. Though they share the most conserved helical amino acid residues of rhodopsin-like GPCRs [39], olfactory receptors definitively form a GPCR subfamily of their own at least in humans. Furthermore, olfactory receptors exhibit peculiar ligand binding properties: one receptor can be activated by several ligands, which in turn induce different levels of signaling [80]. Last, it is possible that odorant recognition is not achieved via olfactory receptors directly, but mediated by odorant-binding proteins [81]. These proteins can absorb odorants, and then bind to the extracellular domain of olfactory receptors [82], turning them into peptide binders. Only a crystal structure of an olfactory receptor will give a definitive answer on the relationship between opsins and olfactory receptors.

Summarizing these results based on structural comparison of el2, the structural features within the available GPCR structures are in line with both our calculated maximum likelihood trees for the crystal structure comparison and for rhodopsin-like GPCRs. Small molecule binding ligands in GPCRs seem to have developed as allosteric binders via an opsin ancestor as key intermediate, with el2 substituting a bound peptide ligand and thus probably stabilizing it. We are aware that the current number of 24 GPCR structures is still too small to make a final statement on the evolution of el2 structure. However, we see the current set of structures to be sufficiently consistent with our theory on el2 evolution to support our model. Along the proposed pathway, small molecule ligands then seem to have substituted el2, which retracted from the binding site and lost the β-hairpin conformation. Rhodopsin can therefore be seen as a good model system to study the structures and activation mechanisms of both peptide and small molecule binding GPCRs [83]. This is in so far interesting, as it would render the sense of light detection to be the origin of development of the small molecule-based hormonal response system in our central nervous system (CNS). This coincides with our visual system being an actual part of our CNS instead of being connected to it via a peripheral nerve [84]. It is possible, that during evolution, light-sensing opsin ancestors developed in nervous tissues, which then led to the development of small molecule receptors in the CNS. A good candidate for such an intermediate is a melanopsin precursor, as melanopsin mediates a large variety of physiological responses to light, and from its sequence is closer related to invertebrate than to vertebrate rhodopsins [85].

We here have to state that all so far listed GPCR crystal structures come from mammalian or avian organisms. They therefore represent GPCR structures at a contemporary state, and thus only allow a limited assessment of GPCR evolution due to missing “fossil” GPCRs. However, as the crystal structure of squid (*Todarodes pacificus*) rhodopsin is available (PDB ID 2Z73) [86], we can verify how well preserved the position of el2 is by a comparison with the structure of bovine rhodopsin [3,36]. Our comparison in S4 Fig shows that the position in both structures is identical, despite the obvious difference in el2 sequences. Consequently, we can make the assumption that the position of el2 is highly conserved in rhodopsins, and needs to have been constant since the last common ancestor of mammals and squids. It thus has to be an evolutionary old feature across the animal kingdom. This supports our hypothesis that the el2 position in rhodopsin represents an evolutionary old transition between peptide and small molecule receptors, which still can be seen in present day structures.

### Opioid receptors exhibit small ligand receptor-like binding pockets and sodium-binding sites

**Fig 5:**
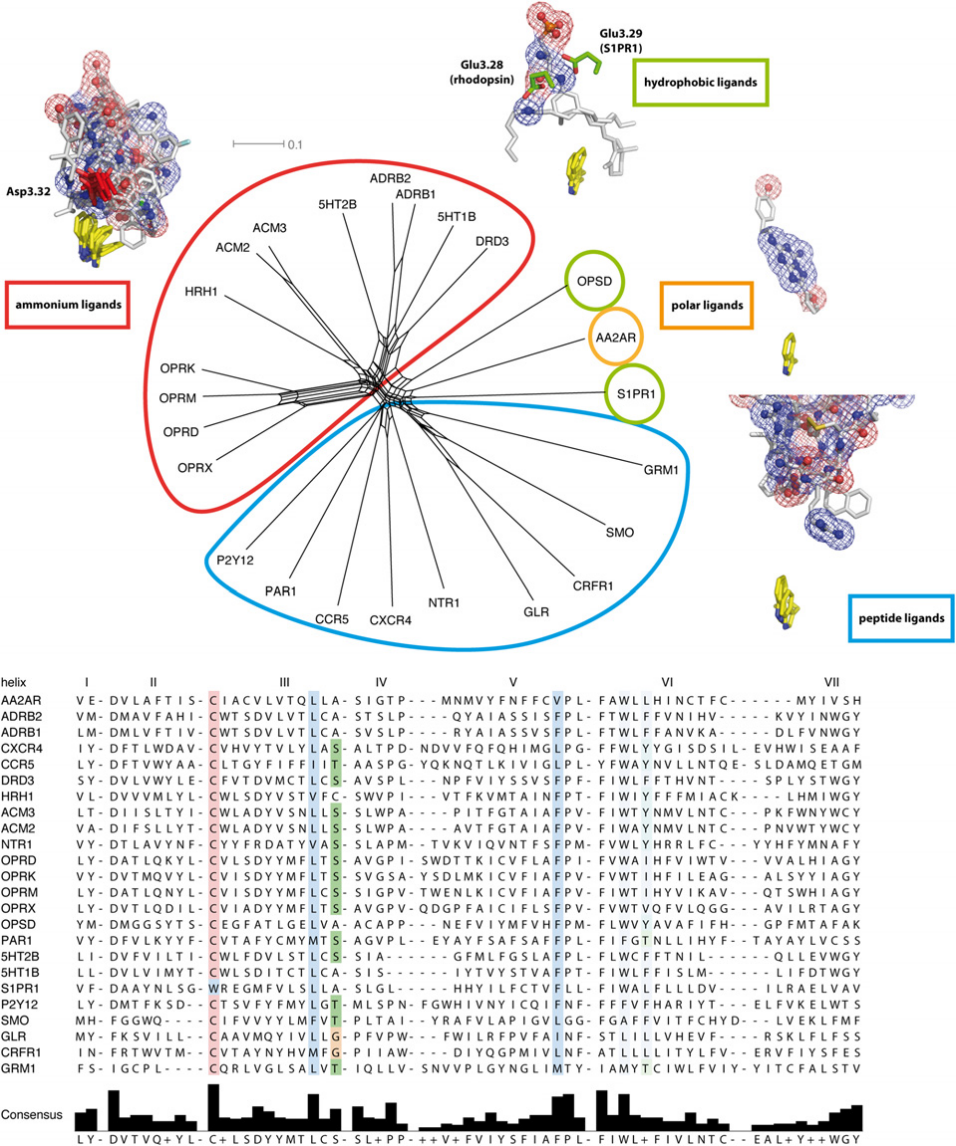
NeighborNet analysis of GPCR ligand binding sites. Top: uncorrected P distance based NeighborNet of residues forming the ligand binding sites in GPCR crystal structures. Ligands and characteristic residues in sticks, their characteristic contact-forming polar oxygen/nitrogen atoms as spheres and mesh. Trp6.48 in yellow displayed as visual point of reference. The network analysis reveals four dominant categories of ligand binding cavities: peptide ligand receptors (cyan), hydrophobic ligand receptors (green), containing opsins and sphingosine receptors, polar ligand receptors (orange), containing adenosine receptors; and ammonium ligand receptors (red), containing muscarinic acetylcholine receptors, biogenic amine receptors, and opioid receptors. Hydrophobic ligand receptors share a glutamate at the similar positions 3.28 or 3.29. Surprisingly, opioid receptors are found in a group together with biogenic amine receptors, but not with peptide ligand receptors. They share the major contact residue Asp3.32 with acetylcholine and biogenic amine receptors. Bottom: Sequence alignment of amino acids forming small molecule binding sites in analyzed GPCR crystal structures.

In the following, we perform a further analysis on the development of the amino acids flanking the ligand binding pockets in our investigated set of crystallized GPCRs. We found that a NeighborNet analysis with maximum likelihood distances resulted in a network with conflicting groupings, observable by widening of the internal faces and missing singular edges for the proteins (see S5 Fig). To get a better picture of the absolute similarity of the binding sites, we performed an uncorrected P distance analysis. This compares the binding sites by counting matching amino acids having the same sequence (and herein therefore spatial) position. A similar analysis was performed on the residues of the retinal binding pocket [87], which we extend now to the set of contact residues for all ligands in the available structures. Fig 5 shows the resulting NeighborNet. Four dominant categories of ligand binding cavities appear: peptide ligand receptors, containing the chemokine receptors and non-rhodopsin GPCRs; hydrophobic ligand receptors, containing the opsins and sphingosine receptors, hydrophilic ligand receptors, containing adenosine receptors; and ammonium ligand receptors, containing muscarinic acetylcholine receptors, biogenic amine receptors, and opioid receptors. Please note that in this framework, we categorize metabotropic glutamate receptors as peptide receptors (see above). Surprisingly, opioid receptors are found in a group together with the biogenic amine receptors, but not with peptide ligand receptors, sharing the major contact residue Asp3.32 with acetylcholine and biogenic amine receptors. However, this aspartate does not seem to be the major source of this similarity, as its removal from the uncorrected P distance analysis does not change the overall topology of the network (see S6 Fig, left). The same holds true to the glutamate positions at positions 3.28 or 3.29, respectively, in hydrophobic ligand receptors (see S6 Fig, right), which form a salt bridge with the respective bound ligand. Possibly, the placing of glutamates at position 3.28 or 3.29, respectively, was an important step in their evolutionary development to become small molecule binders.

**Fig 6:**
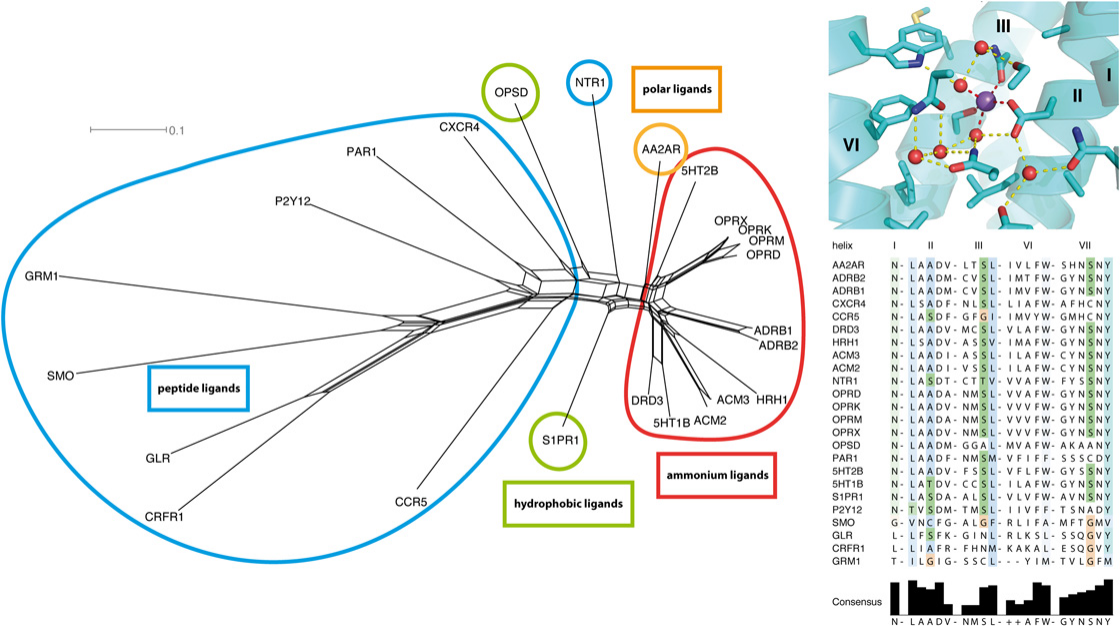
NeighborNet analysis of GPCR sodium binding sites. Uncorrected P distance based NeighborNet analysis of residues forming the central sodium / water binding site in GPCR crystal structures. Left: NeighborNet analysis. The network analysis reveals four dominant categories of ligand binding cavities: peptide ligand receptors (cyan), hydrophobic ligand receptors (green), containing opsins and sphingosine receptors, polar ligand receptors (orange), containing adenosine receptors; and ammonium ligand receptors (red), containing muscarinic acetylcholine receptors, biogenic amine receptors, and opioid receptors. The overall topology of the network is in good agreement with the one of the ligand binding site presented in Fig 5, which suggests an evolutionary connection between both sites. Top right: overview of residues forming the central sodium/water binding site (in PDB ID 4N6H [35]). Sodium in purple sphere, water oxygen atoms in red spheres, binding site forming amino acids as sticks. Bottom right: sequence comparison of analyzed amino acid positions.

We furthermore analyzed the evolutionary relationship of amino acids forming the central sodium binding site in GPCRs [88]. This site is known from AA2AR [20,34], the δ-opioid receptor (OPRD) [35], ADRB1 [89], and PAR1 [19] as an allosteric regulator for agonist binding. Residues forming contacts with the sodium ion were shown to be highly conserved [88]. However, the first coordination shell of the ion is partially formed by protein-bound water molecules. Such protein-internal water molecules are highly important for the structure and function of heptahelical transmembrane proteins [90–92], and show a conserved pattern of positions in GPCRs, as well [93]. We therefore extended the investigation to all residues being in contact with the sodium ion and water molecules in this central binding site. Fig 6 displays the resulting uncorrected P distance network: Surprisingly, the network shares the same general features like the one of the ligand binding site. As both analyzed networks share eight amino acid positions, we removed these residues from the ligand binding site comparison, and re-analyzed the resulting sequence set. S7 Fig shows the resulting NeighborNet: the overall network topology does not change, as well. The high similarity of the network topology for both ligand binding site and sodium/water binding site suggests a common evolutionary path, which is in good agreement with their functional coupling for small molecule binding [34]. The reason for the special placement of opioid receptors in both networks remains to be elucidated: from the results of our data, it seems that opioid receptors may not be exclusively primed to bind endorphins, i.e. peptide ligands, but to have the potential to interact with small molecule ligands, too.

Summing up, our network analysis proposes a coupled evolution of the GPCR ligand binding sites together with the central sodium/water binding sites. It seems that though opioid receptors developed as peptide receptors, both their ligand binding site and central sodium/water binding site are similar to the ones of biogenic amine receptors, suggesting a high degree of homoplasy between both subclasses. Though the primary ligands in opioid signaling in the brain are endorphins, i.e. peptides, the binding site might be primed for the interaction with small molecule ligands. In agreement with this, opioid and β-adrenergic receptor ligands exhibit common pharmacophores [94]. From our data alone, we cannot determine what is the evolutionary driving force for this development. A co-evolution of opiate receptors together with opiates in humans seems unlikely, as even evolutionarily distant organisms like zebrafish show responses to opiates [95]. Another possibility to explain this similarity would be the direct binding of biogenic amines and their derivatives to opioid receptors as a regulatory element in the central nervous system. Last, it might be a coincidental case of homoplasy due to a common usage of a tyrosine-based pharmacophore [94]. In any case, our own opioid receptors seem to be evolutionary primed to small ligand and thus opiate binding, which may pose a physical explanation to the strong action of such drugs on the central nervous system [14,96].

## Conclusions

We here present the first evolutionary analysis of the human GPCR repertoire based on both sequences and structural features of the now available set of 24 GPCR structures, with focus on rhodopsin-like GPCRs. We discriminate them into the subclasses of peptide binding GPCRs, small molecule ligand binding GPCRs, and olfactory receptors. Concerning ligand binding, we propose that the critical step in the evolutionary development of small molecule ligand receptors lies in an opsin ancestor. Crucial therein is the interplay of small molecule ligands and el2 to substitute a bound peptide ligand. The presented evolutionary tree seems to be closely connected to the development of the human central nervous system. In an evolutionary analysis of ligand binding site and central sodium/water binding cavity forming residues, we show that the evolution of these sites is closely coupled. Last, we found that opioid receptors and muscarinic acetylcholine / biogenic amine receptors exhibit homoplasy, possibly explaining the strong affinity of opiates to opioid receptors, despite them being peptide receptors. We hope that these findings will contribute to the overall understanding of the GPCR family, and to neurophysiological, pharmaceutical & biophysical research as well.

## Supporting Information

S1 Fig Maximum likelihood tree analysis of the 7TM domain without the N-/C-termini of non-rhodopsin GPCR sequences. Only four non-rhodopsin structures are available for sequence analysis (the containing branches are highlighted in purple). While some subfamilies seem to be well resolved (glutamate receptors, frizzled/smoothed receptors, secretin receptors, taste receptors type 2, vomeronasal receptors, adhesion receptors), others lack a clear separation from the tree basis (orphan families) or are inexplicably separated over different nodes (taste receptors type I, EGF-like receptors). The tree analysis thus does not give a clear picture of this class of receptors yet. We assume that the currently available four non-rhodopsin GPCR structures do not sufficiently cover the full sequence range of the tree for a phylogenetic analysis.
(TIF)

S2 Fig Comparison of the position of el2 in different intermediates of the rhodopsin photochemical reaction. Dark-state rhodopsin (PDB ID 1U19) [36] in cyan, Meta II (3PXQ [75] and 4A4M [72]) in red, G-protein mimic bound opsin (3DQB) [74] in yellow, and opsin (3CAP) [76] in orange. While meta II—rhodopsin and the two opsin structures show a very similar arrangement of el2, it is different in dark-state rhodopsin.
(TIF)

S3 Fig Comparison of predicted secondary structure of el2 in olfactory receptors (left) and rhodopsin (right). The secondary structure of the el2 of 20 randomly chosen olfactory receptors (see bottom) majorly contains β-strands. A similar prediction results for the el2 of rhodopsin, which is known to form a β-hairpin [3,36]. We therefore assume that the el2 of olfactory receptors exhibit a β-hairpin shape as well, despite the lack of sequence similarity with the el2 of rhodopsin (see bottom; sequence alignment according to Gelis et al. [64]).
(TIF)

S4 Fig Comparison of el2 and the N terminus in rhodopsin (red, PDB ID 1U19) [36] and squid rhodopsin (yellow, PDB ID 2Z73) [86]. Top: conserved peptide binding volume (orange surface) and small molecule binding volume (blue mesh) displayed as in Fig 3. While the N terminus differs in fold, but not in position between both structures, the fold and position of el2 is practically identical in both structures. Bottom: Sequence comparison between squid (OPSD_TODPA, Uniprot accession No. P31356), human (OPSD_HUMAN, P08100), and bovine (OPSD_BOV, P02699) rhodopsin. Human and bovine rhodopsin el2 sequences are almost identical. Their el2 structure should therefore be comparable with each other. Despite the nearly identical shape, squid rhodopsin differs considerably in its sequence from both mammalian rhodopsins.
(TIF)

S5 Fig NeighborNet analysis (JTT model) of the ligand binding pockets. The network shows a considerable spreading of internal nodes and no clear singular edges for proteins, showing no clear tree-like evolution of the respective proteins.
(TIF)

S6 Fig Maximum parsimony NeighborNet analysis (uncorrected P distances) of ligand binding residues without residue position Asp3.32 (left) and Glu3.28 / Glu3.29 (right). Both removal of Asp3.32 and Glu3.28 / 3.29 is not altering the network topology.
(TIF)

S7 Fig Maximum parsimony NeighborNet analysis (uncorrected P distances) of ligand binding residues without residues involved in the sodium binding site. Removing the amino acids involved in both ligand and sodium binding site does not alter the overall network topology.
(TIF)

S1 File Crystal structure-based alignment in FASTA format. (DOCX)

S2 File Subgroup members and respective structures used for 7TM domain determination. (DOCX)

S3 File Full non-rhodopsin GPCR tree in Newick format. (DOCX)

S4 File Full rhodopsin-like GPCR tree in Newick format (DOCX)

S5 File Alignment of non-rhodopsin GPCR 7TM sequences in FASTA format. (FA)

S6 File Alignment of rhodopsin-like GPCR 7TM sequences in FASTA format. (FA)
