# Supplementary figures and images for: Sequence, Structure and Ligand Binding Evolution of Rhodopsin-Like G Protein-Coupled Receptors: A Crystal Structure-Based Phylogenetic Analysis
Source: PLoS One. 2015 Apr 16;10(4):e0123533. doi: 10.1371/journal.pone.0123533 (PMC4399913; doi:10.1371/journal.pone.0123533)

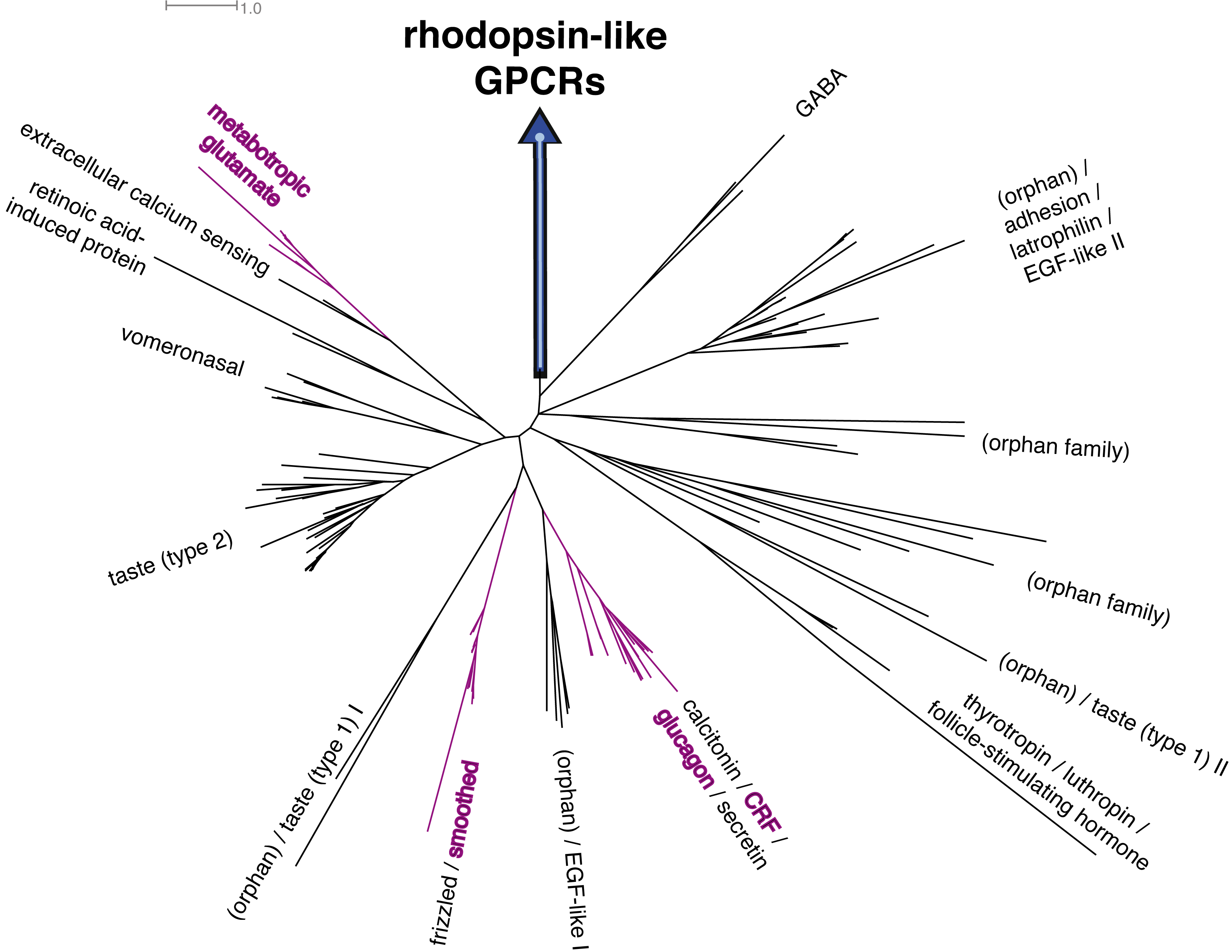

Supplement: S1 Fig — Only four non-rhodopsin structures are available for sequence analysis (the containing branches are highlighted in purple). While some subfamilies seem to be well resolved (glutamate receptors, frizzled/smoothed receptors, secretin receptors, taste receptors type 2, vomeronasal receptors, adhesion receptors), others lack a clear separation from the tree basis (orphan families) or are inexplicably separated over different nodes (taste receptors type I, EGF-like receptors). The tree analysis thus does not give a clear picture of this class of receptors yet. We assume that the currently available four non-rhodopsin GPCR structures do not sufficiently cover the full sequence range of the tree for a phylogenetic analysis. (TIF) [file pone.0123533.s001.tif]

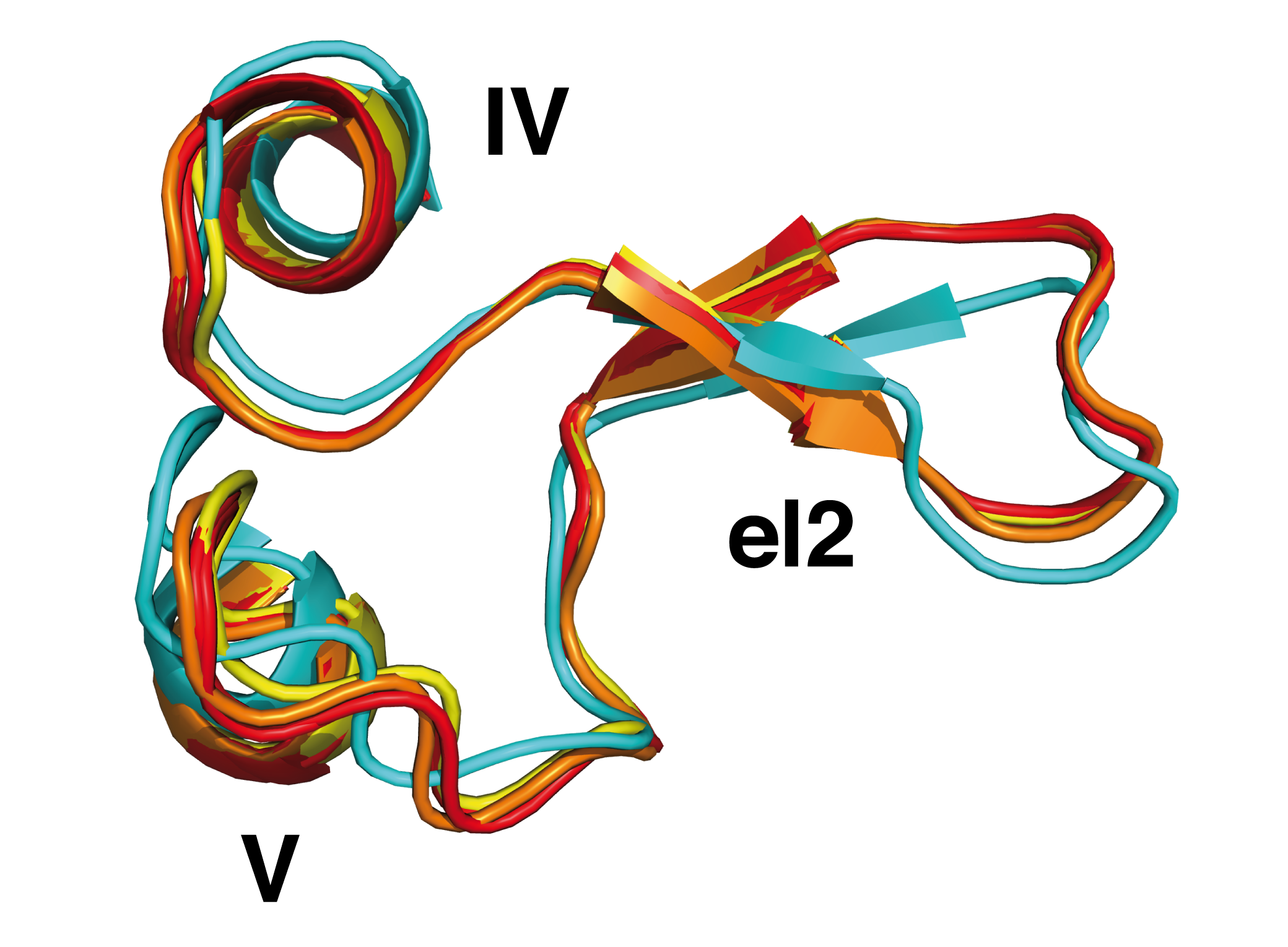

Supplement: S2 Fig — Dark-state rhodopsin (PDB ID 1U19) [36] in cyan, Meta II (3PXQ [75] and 4A4M [72]) in red, G-protein mimic bound opsin (3DQB) [74] in yellow, and opsin (3CAP) [76] in orange. While meta II—rhodopsin and the two opsin structures show a very similar arrangement of el2, it is different in dark-state rhodopsin. (TIF) [file pone.0123533.s002.tif]

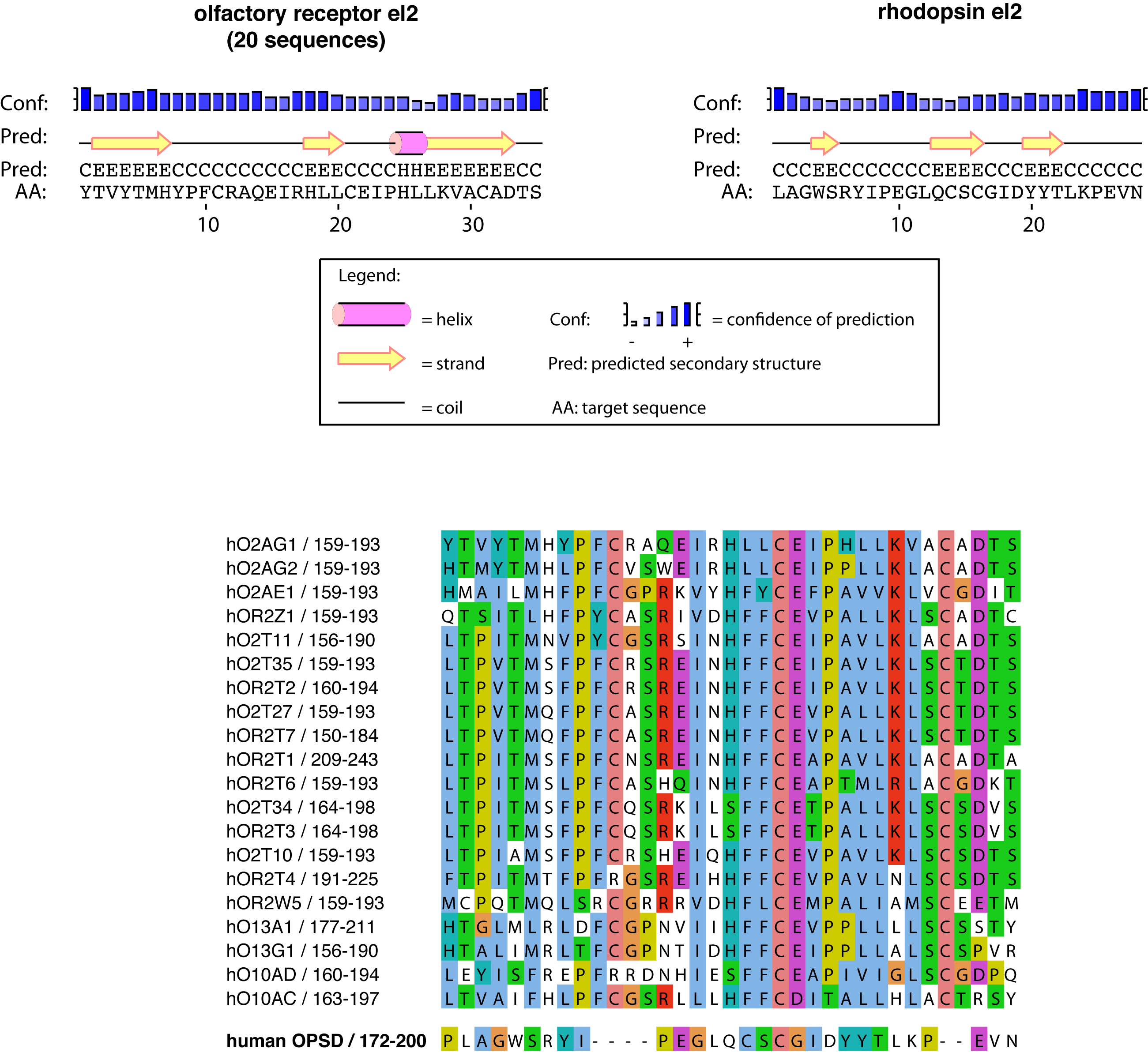

Supplement: S3 Fig — The secondary structure of the el2 of 20 randomly chosen olfactory receptors (see bottom) majorly contains β-strands. A similar prediction results for the el2 of rhodopsin, which is known to form a β-hairpin [3,36]. We therefore assume that the el2 of olfactory receptors exhibit a β-hairpin shape as well, despite the lack of sequence similarity with the el2 of rhodopsin (see bottom; sequence alignment according to Gelis et al. [64]). (TIF) [file pone.0123533.s003.tif]

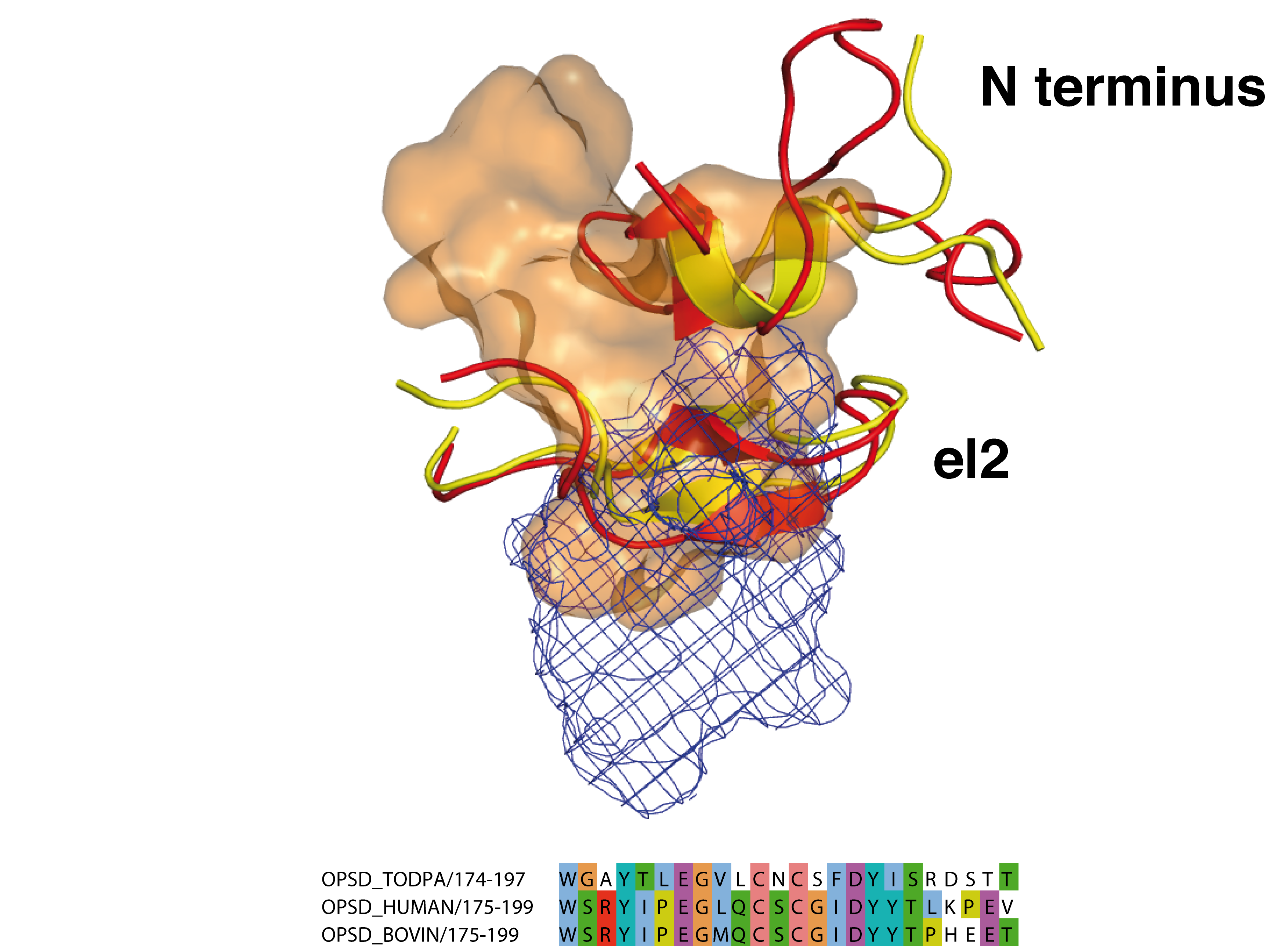

Supplement: S4 Fig — Top: conserved peptide binding volume (orange surface) and small molecule binding volume (blue mesh) displayed as in Fig 3. While the N terminus differs in fold, but not in position between both structures, the fold and position of el2 is practically identical in both structures. Bottom: Sequence comparison between squid (OPSD_TODPA, Uniprot accession No. P31356), human (OPSD_HUMAN, P08100), and bovine (OPSD_BOV, P02699) rhodopsin. Human and bovine rhodopsin el2 sequences are almost identical. Their el2 structure should therefore be comparable with each other. Despite the nearly identical shape, squid rhodopsin differs considerably in its sequence from both mammalian rhodopsins. (TIF) [file pone.0123533.s004.tif]

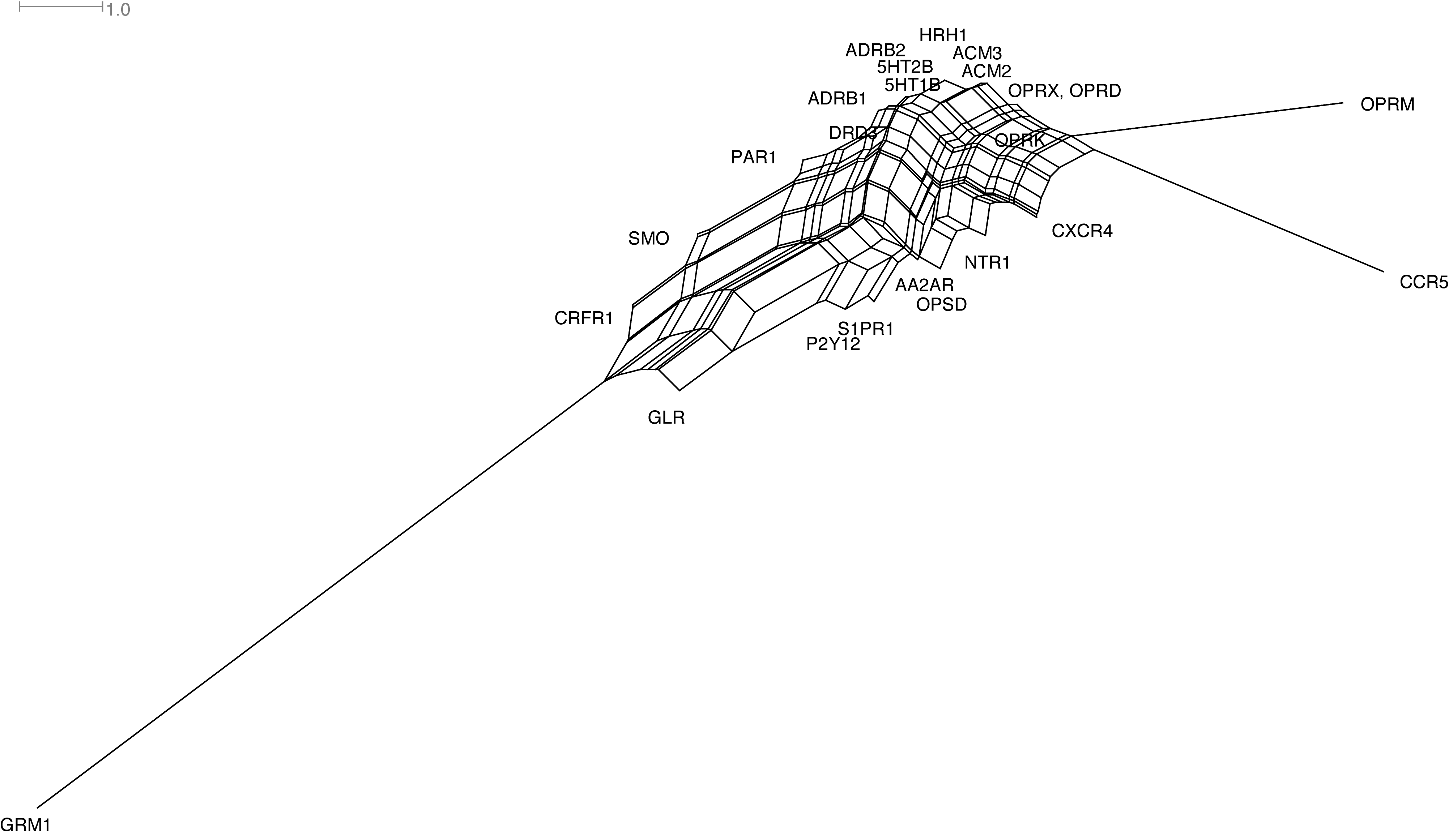

Supplement: S5 Fig — The network shows a considerable spreading of internal nodes and no clear singular edges for proteins, showing no clear tree-like evolution of the respective proteins. (TIF) [file pone.0123533.s005.tif]

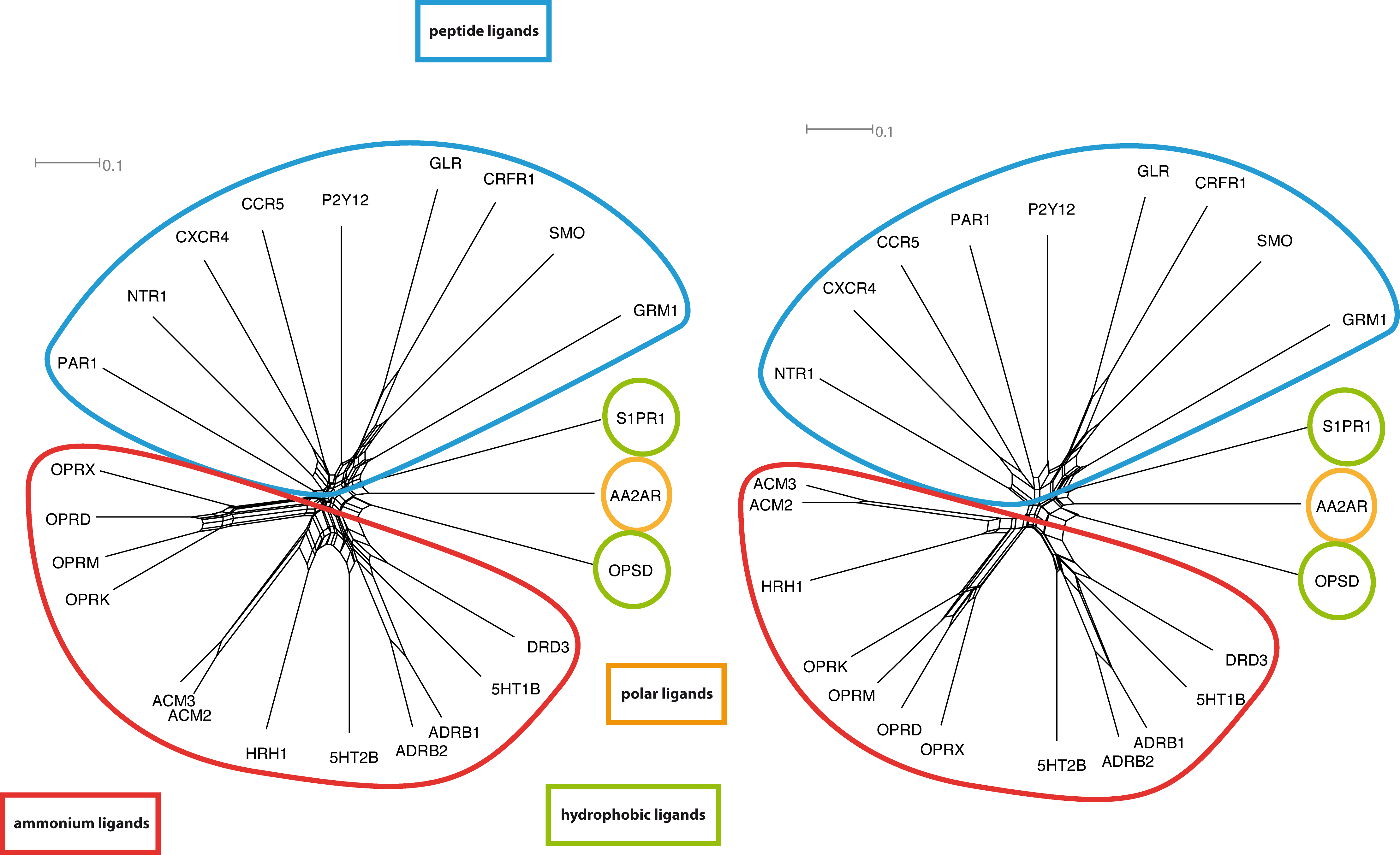

Supplement: S6 Fig — Both removal of Asp3.32 and Glu3.28 / 3.29 is not altering the network topology. (TIF) [file pone.0123533.s006.tif]

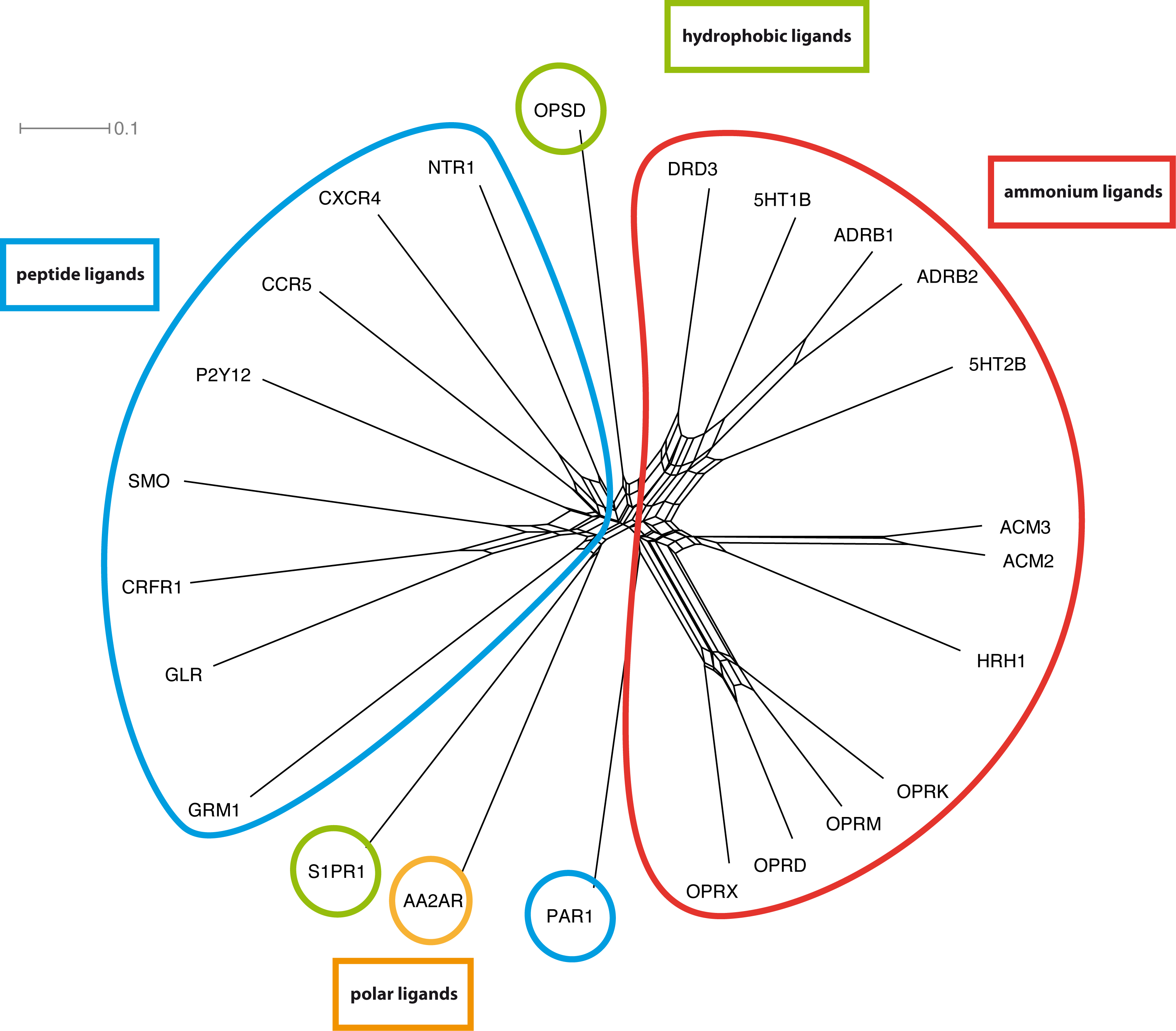

Supplement: S7 Fig — Removing the amino acids involved in both ligand and sodium binding site does not alter the overall network topology. (TIF) [file pone.0123533.s007.tif]
