# Supplementary material for: Sequence, Structure and Ligand Binding Evolution of Rhodopsin-Like G Protein-Coupled Receptors: A Crystal Structure-Based Phylogenetic Analysis
Source: PLoS One. 2015 Apr 16;10(4):e0123533. doi: 10.1371/journal.pone.0123533 (PMC4399913; doi:10.1371/journal.pone.0123533)
Supplement: S1 File — (DOCX) [file pone.0123533.s008.docx]

>AA2ARRHELIX/1-286

--IMGSSVYITVELAIAVLAILGNVLVCWAVWL-------------------------------NSNLQNV-

--------TNYFVVSLAAADIAVGVLAIPFAITIST---------------------GFCAA---------C

HGCLFIACFVLVLTQSSIFSLLAIAIDRYIAIRI-----PLRYNGLVTG-------TRAKGIIAICWVLSFA

IGLTPMLG-----WNNCGQPKEGKNHSQGCGEGQVACLFEDVVP------------MNYMVYFNFFACVLVP

LLLMLGVYLRIFLAARRQ------------------------------------------------------

------------------------------------------------------------------------

--------------------------------------------LKQMESQPLPGERAR-------------

---------------------------------------STLQKEVHAAKSLAIIVGLFALCWLPLHIINCF

TFFC----------------------------------PDCSHAPLWL-----------MYLAIVLSHTNSV

VNPFIY-----

>ADRB2RHELIX/1-298

---VWVVGMGIVMSLIVLAIVFGNVLVITAIAK-------------------------------FERLQT--

-------VTNYFITSLACADLVMGLAVVPFGAAHILM-------------------KMWTFGN---------

FWCEFWTSIDVLCVTASIETLCVIAVDRYFAIT-----SPFKYQSLLT-------KNKARVIILMVWIVSGL

TSFLPIQM---------HWYRATHQEAINCYANETCCDFFTN---------------QAYAIASSIVSFYVP

LVIMVFVYSRVFQEAKRQ------------------------------------------------------

------------------------------------------------------------------------

--------------------------------LQKIDKSEGRFHVQNLSQVEQDGRTGH-------------

-------------GLRRSSKFCL--------------------KEHKALKTLGIIMGTFTLCWLPFFIVNIV

HVI-----------------------------------QDNLIR------------KEVYILLNWIGYVNSG

FNPLIYCR---

>ADRB1RHELIX/1-324

---QWTAGMGLLMALIVLLIVAGNVLVIVAIAK-------------------------------TPRLQT--

-------LTNLFIMSLASADLVMGLLVVPFGATIVVW-------------------GRWEYGS---------

FFCELWTSVDVLCVTASIETLCVIALDRYLAIT-----SPFRYQSLLT-------RARARGLVCTVWAISAL

VSFLPILM---------HWWRAESDEARRCYNDPKCCDFVTN---------------RAYAIASSVVSFYVP

LCIMAFVYLRVFREAQKQ------------------------------------------------------

------------------------------------------------------------------------

-----VKKIDSCERRFLGGPARPPSPSPSPVPAPAPPPGPPRPAAAAATAPLANGRAGK-------------

---------------RRPSRLVAL-------------------REQKALKTLGIIMGVFTLCWLPFFLANVV

KAF-----------------------------------HRELVP------------DRLFVFFNWLGYANSA

FNPIIYCR---

>CXCR4RHELIX/1-271

NANFNKIFLPTIYSIIFLTGIVGNGLVILVM-------------------------------GYQKKLR---

------SMTDKYRLHLSVADLLFVITLPFWAVDAVA---------------------NWY---------FGN

FLCKAVHVIYTVNLYSSVLILAFISLDRYLAI-----VHATNSQRPRK-------LLAEKVVYVGVWIPALL

LTIPDFIF-----------------ANVSEADDRYICDRFYP----------NDLWVVVFQFQHIMVGLILP

GIVILSCYCIIISKLSH-------------------------------------------------------

------------------------------------------------------------------------

----------------------------------------------------SKGHQKR-------------

----------------------------------------------KALKTTVILILAFFACWLPYYIGISI

DSFIL----------------------------LEIIKQGCE---------FENTVHKWISITEALAFFHCC

LNPILYA----

>CCR5RHELIX/1-274

VKQIAARLLPPLYSLVFIFGFVGNMLVILIL-------------------------------INCKRLK---

------SMTDIYLLNLAISDLFFLLTVPFWAHYAAA---------------------QWD---------FGN

TMCQLLTGLYFIGFFSGIFFIILLTIDRYLAV-----VHAVFALKART-------VTFGVVTSVITWVVAVF

ASLPGIIF-----------------TRSQKEGLHYTCSSHFPYSQ-------YQFWKNFQTLKIVILGLVLP

LLVMVICYSGILKTLLR-------------------------------------------------------

------------------------------------------------------------------------

---------------------------------------------------CRNEKKRH-------------

----------------------------------------------RAVRLIFTIMIVYFLFWAPYNIVLLL

NTFQE-----------------------------FFGLNNCS---------SSNRLDQAMQVTETLGMTHCC

INPIIYA----

>DRD3RHELIX/1-350

----------LSYCALILAIVFGNGLVCMAVLKE-------------------------------RALQ---

------TTTNYLVVSLAVADLLVATLVMPWVVYLEVT------------------GGVWNF---------SR

ICCDVFVTLDVMMCTASILNLCAISIDRYTAVV-----MPVHYQHGTGQS----SCRRVALMITAVWVLAFA

VSCPLLF-------------------GFNTTGDPTVCSIS----------------NPDFVIYSSVVSFYLP

FGVTVLVYARIYVVLKQ-------------------------------------------------------

----------------------------------------------------------------------RR

RKRILTRQNSQCNSVRPGFPQQTLSPDPAHLELKRYYSICQDTALGGPGFQERGGELKREEKTRNSLSPTIA

PKLSLEVRKLSNGRLSTSLKLGPLQPRGV------------PLREKKATQMVAIVLGAFIVCWLPFFLTHVL

NTH----------------------------------CQTCHV------------SPELYSATTWLGYVNSA

LNPVIY-----

>HRH1RHELIX/1-442

------MPLVVVLSTICLVTVGLNLLVLYAVRSE-------------------------------RKLH---

------TVGNLYIVSLSVADLIVGAVVMPMNILYLLM-------------------SKWS---------LGR

PLCLFWLSMDYVASTASIFSVFILCIDRYRSVQQ-----PLRYLKYR-------TKTRASATILGAWFLSFL

WVIPILG--------------WNHFMQQTSVRREDKCETDFYDV------------VTWFKVMTAIINFYLP

TLLMLWFYAKIYKAV---------------------------------------RQHCQHRELINRSLPSFS

EIKLRPENPKGDAKKPGKESPWEVLKRKPKDAGGGSVLKSPSQTPKEMKSPVVFSQEDDREVDKLYCFPLDI

VHMQAAAEGSSRDYVAVNRSHGQLKTDEQGLNTHGASEISEDQMLGDSQSFSRTDSDTTTETAPGKGKLRSG

SNTGLDYIKFTWKRLRSHSRQYVSGLH--------------MNRERKAAKQLGFIMAAFILCWIPYFIFFMV

IAFCK---------------------------------NCCNEH--------------LHMFTIWLGYINST

LNPLIY-----

>ACM3RHELIX/1-478

----QVVFIAFLTGILALVTIIGNILVIVSFKV-------------------------------NKQLKTV-

--------NNYFLLSLACADLIIGVISMNLFTTYIIM-------------------NRWAL---------GN

LACDLWLAIDYVASNASVMNLLVISFDRYFSITR-----PLTYRAKRT-------TKRAGVMIGLAWVISFV

LWAPAILFWQYF--------------VGKRTVPPGECFIQFLSE-------------PTITFGTAIAAFYMP

VTIMTILYWRIYKETE-----KRTKELAGLQASGTEAETENFVHPTGSSRSCSSYELQQQSMKRSNRRKYGR

CHFWFTTKSWKPSSEQMDQDHSSSDSWNNNDAAASLENSASSDEEDIGSETRAIYSIVLKLPGHSTILNSTK

LPSSDNLQVPEEELGMVDLERKADKLQAQKSVDDGGSFPKSFSKLPIQLESAVDTAKTSDVNSSVGKSTATL

PLSFKEATLAKRFALKTRSQITKRKRMSLVKE-------------KKAAQTLSAILLAFIITWTPYNIMVLV

NTFC----------------------------------DSCI-------------PKTFWNLGYWLCYINST

VNPVCY-----

>ACM2RHELIX/1-419

----EVVFIVLVAGSLSLVTIIGNILVMVSIKV-------------------------------NRHLQTV-

--------NNYFLFSLACADLIIGVFSMNLYTLYTVI-------------------GYWPL---------GP

VVCDLWLALDYVVSNASVMNLLIISFDRYFCVTK-----PLTYPVKRT-------TKMAGMMIAAAWVLSFI

LWAPAILFWQFI--------------VGVRTVEDGECYIQFFSN-------------AAVTFGTAIAAFYLP

VIIMTVLYWHISRASK-------------------------------------------------SRIKKDK

KEPVANQDPVSPSLVQGRIVKPNNNNMPSSDDGLEHNKIQNGKAPRDPVTENCVQGEEKESSNDSTSVSAVA

SNMRDDEITQDENTVSTSLGHSKDENSKQTCIRIGTKTPKSDSCTPTNTTVEVVGSSGQ------------N

GDEKQNIVARKIVKMTKQPAKKKPPPSRE----------------KKVTRTILAILLAFIITWAPYNVMVLI

NTFC----------------------------------APCI-------------PNTVWTIGYWLCYINST

INPACY-----

>NTR1RHELIX/1-310

--IYSKVLVTAVYLALFVVGTVGNTVTAFTL-------------------------------ARKKSLQSL-

-----QSTVHYHLGSLALSDLLTLLLAMPVELYNF-----------------IWVHHPWAFGD---------

AGCRGYYFLRDACTYATALNVASLSVERYLAICH-----PFKAKTLMSRSR-------TKKFISAIWLASAL

LAVPMLFT-----------MGEQNRSADGQHAGGLVCTPTIH-----------TATVKVVIQVNTFMSFIFP

MVVISVLNTIIANKLTV-------------------------------------------------------

------------------------------------------------------------------------

---------------------------------------MVRQAAEQGQVCTVGGEHST-------------

------------FSMAIEPGRV--------------------QALRHGVRVLRAVVIAFVVCWLPYHVRRLM

FCY----------------------------------ISDEQWTPF-----LYDFYHYFYMVTNALFYVSST

INPILYNLVSA

>OPRDRHELIX/1-273

--LALAIAITALYSAVCAVGLLGNVLVMFGIVR-------------------------------YTKMKTAT

N---------IYIFNLALADALATSTLPFQSAKYLME--------------------TWP---------FGE

LLCKAVLSIDYYNMFTSIFTLTMMSVDRYIAV-----CHPVKALDFR-------TPAKAKLINICIWVLASG

VGVPIMVM-----------------AVTRPRDGAVVCMLQFPS--------PSWYWDTVTKICVFLFAFVVP

ILIITVCYGLMLLRLRSV------------------------------------------------------

------------------------------------------------------------------------

--------------------------------------------------RLLSGSKEK-------------

-----------------------------------------DRSLRRITRMVLVVVGAFVVCWAPIHIFVIV

WTLV----------------------------------DIDRRDPL---------VVAALHLCIALGYANSS

LNPVLY-----

>OPRKRHELIX/1-275

--PAIPVIITAVYSVVFVVGLVGNSLVMFVIIR-------------------------------YTKMKTAT

N---------IYIFNLALADALVTTTMPFQSTVYLMN--------------------SWP---------FGD

VLCKIVISIDYYNMFTSIFTLTMMSVDRYIAV-----CHPVKALDFR-------TPLKAKIINICIWLLSSS

VGISAIVL---------------GGTKVREDVDVIECSLQFPDD-------DYSWWDLFMKICVFIFAFVIP

VLIIIVCYTLMILRLKSV------------------------------------------------------

------------------------------------------------------------------------

--------------------------------------------------RLLSGSREK-------------

-----------------------------------------DRNLRRITRLVLVVVAVFVVCWTPIHIFILV

EALG----------------------------------STSHSTA----------ALSSYYFCIALGYTNSS

LNPILY-----

>OPRMRHELIX/1-272

--MITAITIMALYSIVCVVGLFGNFLVMYVIVR-------------------------------YTKMKTAT

N---------IYIFNLALADALATSTLPFQSVNYLMG--------------------TWP---------FGT

ILCKIVISIDYYNMFTSIFTLCTMSVDRYIAV-----CHPVKALDFR-------TPRNAKIINVCNWILSSA

IGLPVMFM-----------------ATTKYRQGSIDCTLTFSH--------PTWYWENLLKICVFIFAFIMP

VLIITVCYGLMILRLKSV------------------------------------------------------

------------------------------------------------------------------------

--------------------------------------------------RMLSGSKEK-------------

-----------------------------------------DRNLRRITRMVLVVVAVFIVCWTPIHIYVII

KALV--------------------------------TIPETTF------------QTVSWHFCIALGYTNSC

LNPVLY-----

>OPRXRHELIX/1-272

--LGLKVTIVGLYLAVCVGGLLGNCLVMYVILR-------------------------------HTKMKTAT

N---------IYIFNLALADTLVLLTLPFQGTDILLG--------------------FWP---------FGN

ALCKTVIAIDYYNMFTSTFTLTAMSVDRYVAI-----CHPIRALDVR-------TSSKAQAVNVAIWALASV

VGVPVAIM-----------------GSAQVEDEEIECLVEIPT--------PQDYWGPVFAICIFLFSFIVP

VLVISVCYSLMIRRLRGV------------------------------------------------------

------------------------------------------------------------------------

--------------------------------------------------RLLSGSREK-------------

-----------------------------------------DRNLRRITRLVLVVVAVFVGCWTPVQVFVLA

QGLG--------------------------------VQPSSET------------AVAILRFCTALGYVNSC

LNPILY-----

>OPSDRHELIX/1-273

---WQFSMLAAYMFLLIVLGFPINFLTLYVTVQH-------------------------------KKLR---

------TPLNYILLNLAVADLFMVLGGFTSTLYTSLHG-------------------YFV---------FGP

TGCNLEGFFATLGGEIALWSLVVLAIERYVVVC-----KPMSNFRF--------GENHAIMGVAFTWVMALA

CAAPPLA----------------GWSRYIPEGLQCSCGIDYYTLKPEV-------NNESFVIYMFVVHFTIP

MIIIFFCYG---------------------------------------------------------------

------------------------------------------------------------------------

-------------------------------------------QLVFTVKEAAAQQQES-------------

--------------------------------------ATTQKAEKEVTRMVIIMVIAFLICWVPYASVAFY

IFTH----------------------------------QGSNF------------GPIFMTIPAFFAKSAAI

YNPVIYI----

>PAR1RHELIX/1-276

--SWLTLFVPSVYTGVFVVSLPLNIMAIVVFI-------------------------------LKMKVKKP-

--------AVVYMLHLATADVLFVSVLPFKISYYFS-------------------GSDWQF---------GS

ELCRFVTAAFYCNMYASILLMTVISIDRFLAVVY-----PMQSLSWR-------TLGRASFTCLAIWALAIA

GVVPLLL---------------KEQTIQVPGLNITTCHDVLNETLL-------EGYYAYYFSAFSAVFFFVP

LIISTVCYVSIIRCL---------------------------------------------------------

------------------------------------------------------------------------

-----------------------------------------------------SSSAVA-------------

----------------------------------------NRSKKSRALFLSAAVFCIFIICFGPTNVLLIA

HYSF--------------------------------LSHTST----------TEAAYFAYLLCVCVSSISCC

IDPLIYYYA--

>5HT2BRHELIX/1-333

--GNKLHWAALLILMVIIPTIGGNTLVILAVSL-------------------------------EKKLQY--

-------ATNYFLMSLAVADLLVGLFVMPIALLTIMF------------------EAMWPLP---------L

VLCPAWLFLDVLFSTASIMHLCAISVDRYIAIK-----KPIQANQYNS-------RATAFIKITVVWLISIG

IAI---------------PVPIKGIETDVDNPNNITCVLTKERF-------------GDFMLFGSLAAFFTP

LAIMIVTYFLTIHALQK-------------------------------------------------------

------------------------------------------------------------------------

---------------KAYLVKNKPPQRLTWLTVSTVFQRDETPCSSPEKVAMLDGSRKD----------KAL

PNSGDETLMRRTSTIGKKSVQTISNEQ-------------------RASKVLGIVFFLFLLMWCPFFITNIT

L----------------------------------VLCDSCNQTTL----------QMLLEIFVWIGYVSSG

VNPLVYTLF--

>5HT1BRHELIX/1-327

--LPWKVLLVMLLALITLATTLSNAFVIATVYR-------------------------------TRKLHT--

-------PANYLIASLAVTDLLVSILVMPISTMYTVT-------------------GRWTLG---------Q

VVCDFWLSSDITCCTASILHLCVIALDRYWAIT-----DAVEYSAKRT-------PKRAAVMIALVWVFSIS

ISL-----------------PPFFWRQAKAEEEVSECVVNTDH--------------ILYTVYSTVGAFYFP

TLLLIALYGRIYVEARS-------------------------------------------------------

----------------------------------------------------------RILKQTPNRTGKRL

TRAQLITDSPGSTSSVTSINSRVPDVPSESGSPVYVNQVKVRVSDALLEKKKLMAARER-------------

----------------------------------------------KATKTLGIILGAFIVCWLPFFIISLV

M-------------------------------------PICKDACWFH--------LAIFDFFTWLGYLNSL

INPIIYTMS--

>S1PR1RHELIX/1-269

---NSIKLTSVVFILICCFIILENIFVLLTIWKT-------------------------------KKFH---

------RPMYYFIGNLALSDLLAGVAYTANLLLSGATTY------------------KL-----------TP

AQWFLREGSMFVALSASVFSLLAIAIERYITML------KMKLHNGSN-------NFRLFLLISACWVISLI

LGGL-------------------PIMGWNCISALSSCSTVLPLY-------------HKHYILFCTTVFTLL

LLSIVILYCRIYSLVRTRS-----------------------------------------------------

------------------------------------------------------------------------

--------------------------------------------RRLTFRKNISKASRS-------------

-----------------------------------------SEKSLALLKTVIIVLSVFIACWAPLFILLLL

DV--------------------------------GCKVKTCD-------------ILFRAEYFLVLAVLNSG

TNPIIY-----

>P2Y12RHELIX/1-281

-YKITQVLFPLLYTVLFFVGLITNGLAMRIFFQ--------------------------------IRSKS--

-------NFIIFLKNTVISDLLMILTFPFKILSD----------------------AKLGTGPLR------T

FVCQVTSVIFYFTMYISISFLGLITIDRYQKT-----TRPFKTSNPKN-------LLGAKILSVVIWAFMFL

LSLPNMI----------------LTNRQPRDKNVKKCSFLK---------SEFGLVWHEIVNYICQVIFWIN

FLIVIVCYTLITKEL---------------------------------------------------------

------------------------------------------------------------------------

----------------------------------------YRSYVRTRGVGKVPRKKVN-------------

-------------------------------------------------VKVFIIIAVFFICFVPFHFARIP

YT-------------------------------------LSQTRDVFD-CTAENTLFYVKESTLWLTSLNAC

LDPFIYFFL--

>SMORHELIX/1-313

-----EAEHQDMHSYIAAFGAVTGLCTLFTLATFV---------------------------ADWRNSNRY-

--------PAVILFYVNACFFVGSIGWLAQF------------MDGARREIVCRADGTMRLGEPTSNE----

LSCVIIFVIVYYALMAGVVWFVVLTYAWHTSFKALG-TTTYQPLSGKTSY-----------FHLLTWSLPFV

LTVAILAV-----------------AQVDGDSVSGICFVGYKN----------YRYRAGFVLAPIGLVLIVG

GYFLIRGVMTLFSIKSN-------------------------------------------------------

------------------------------------------------------------------------

-------------------------------------------------HPGLLSEKAA-------------

----------------------------------------------SKINETMLRLGIFGFLAFGFVLITFS

CHFYDFFNQA--EWERSFRDYVLCQANVTIGLPTKQPIPDCEIKNRPS--------LLVEKINLFAMFGTGI

AMSTWV-----

>GLRRHELIX/1-267

----MYSSFQVMYTVGYSLSLGALLLALAIL-------------------------------GGLSKLHCTR

NA---------IHANLFASFVLKASSVLVIDGLL--------RTRYSQKIGDDLSVSTWLSDGAV-------

AGCRVAAVFMQYGIVANYCWLLVEGLYLHNLLGL----ATLPERSF------------FSLYLGIGWGAPML

FVVPWAVVKCLF--------------------ENVQCWTSNDN-----------MGFWWILRFPVFLAILIN

FFIFVRIVQLLVAKL---------------------------------------------------------

------------------------------------------------------------------------

--------------------------------------------------RARQMHHTD-------------

---------------------------------------------------YKFRLAKSTLTLIPLLGVHEV

VFA-----------------------------------FVTDEHAQGT------LRSAKLFFDLFLSSFQGL

LVAVLYCFL--

>CRFR1RHELIX/1-283

----HYHVAVIINYLGHCISLVALLVAFVLFLR--LRPGCTHWGDQADGALEVGAPWSGAPFQVRRSIRC--

-------LRNIIHWNLISAFILRNATWFVVQLTM----------------------SPEVHQSNV-------

GWCRLVTAAYNYFHVTNFFWMFGEGCYLHTAIV-------LTYST---------DRLRKWMFICIGWGVPFP

IIVAWAIGKLYY--------------------DNEKCWFGKRPGVYT----------DYIYQGPMILVLLIN

FIFLFNIVRILMTK----------------------------------------------------------

------------------------------------------------------------------------

----------------------------------------------------LRASTT--------------

-----------------------------------------------SETIQYRKAVKATLVLLPLLGITYM

LFFV---------------------------------NPGED----------EVSRVVFIYFNSFLESFQGF

FVSVFYCFLNS

>GRM1RHELIX/1-243

--NIESIIAIAFSCLGILVTLFVTLIFVLY--------------------------------RDTPVVKSS-

--------SRELCYIILAGIFLGYVCPFTLIA--------------------------KP----------TT

TSCYLQRLLVGLSSAMCYSALVTKTNRIARIL-----AGSKKKICTRKPRFM-----SAWAQVIIASILISV

QLTLVVTLIIME-------PPMPILSYPSIKEVYLICNT-------------SNLGVVAPLGYNGLLIMSCT

YYAFKT------------------------------------------------------------------

------------------------------------------------------------------------

--------------------------------------------------------RNV-------------

--------------------------------------------------------KYIAFTMYTTCIIWLA

FVPIYFGS---------------------------------------N----YKIITTCFAVSLSVTVALGC

MFTPKMYIII-
