# Supplementary material for: Sequence, Structure and Ligand Binding Evolution of Rhodopsin-Like G Protein-Coupled Receptors: A Crystal Structure-Based Phylogenetic Analysis
Source: PLoS One. 2015 Apr 16;10(4):e0123533. doi: 10.1371/journal.pone.0123533 (PMC4399913; doi:10.1371/journal.pone.0123533)
Supplement: S2 File — (DOCX) [file pone.0123533.s009.docx]

Subgroups used for extraction of 7TM sequences:

Group 1:

>sp|Q9HAR2|LPHN3_HUMAN/1-1447 Latrophilin-3 OS=Homo sapiens GN=LPHN3 PE=1 SV=2

>sp|O94910|LPHN1_HUMAN/1-1474 Latrophilin-1 OS=Homo sapiens GN=LPHN1 PE=1 SV=1

>sp|O95490|LPHN2_HUMAN/1-1459 Latrophilin-2 OS=Homo sapiens GN=LPHN2 PE=1 SV=2

>sp|Q9BXB1|LGR4_HUMAN/1-951 Leucine-rich repeat-containing G-protein coupled receptor 4 OS=Homo sapiens GN=LGR4 PE=2 SV=2

>sp|Q9HBX8|LGR6_HUMAN/1-967 Leucine-rich repeat-containing G-protein coupled receptor 6 OS=Homo sapiens GN=LGR6 PE=1 SV=3

>sp|Q8IZP9|GPR64_HUMAN/1-1017 G-protein coupled receptor 64 OS=Homo sapiens GN=GPR64 PE=1 SV=2

>sp|Q8IWK6|GP125_HUMAN/1-1321 Probable G-protein coupled receptor 125 OS=Homo sapiens GN=GPR125 PE=1 SV=2

>sp|Q96PE1|GP124_HUMAN/1-1338 G-protein coupled receptor 124 OS=Homo sapiens GN=GPR124 PE=1 SV=2

>sp|O14514|BAI1_HUMAN/1-1584 Brain-specific angiogenesis inhibitor 1 OS=Homo sapiens GN=BAI1 PE=1 SV=1

>sp|O60242|BAI3_HUMAN/1-1522 Brain-specific angiogenesis inhibitor 3 OS=Homo sapiens GN=BAI3 PE=1 SV=2

>sp|O60241|BAI2_HUMAN/1-1585 Brain-specific angiogenesis inhibitor 2 OS=Homo sapiens GN=BAI2 PE=2 SV=2

Group 2:

>sp|Q9NPG1|FZD3_HUMAN/1-666 Frizzled-3 OS=Homo sapiens GN=FZD3 PE=1 SV=1

>sp|O60353|FZD6_HUMAN/1-706 Frizzled-6 OS=Homo sapiens GN=FZD6 PE=1 SV=2

>sp|Q9ULV1|FZD4_HUMAN/1-537 Frizzled-4 OS=Homo sapiens GN=FZD4 PE=1 SV=2

>sp|Q9ULW2|FZD10_HUMAN/1-581 Frizzled-10 OS=Homo sapiens GN=FZD10 PE=2 SV=1

>sp|O00144|FZD9_HUMAN/1-591 Frizzled-9 OS=Homo sapiens GN=FZD9 PE=2 SV=1

>sp|Q14332|FZD2_HUMAN/1-565 Frizzled-2 OS=Homo sapiens GN=FZD2 PE=2 SV=1

>sp|Q9UP38|FZD1_HUMAN/1-647 Frizzled-1 OS=Homo sapiens GN=FZD1 PE=1 SV=2

>sp|O75084|FZD7_HUMAN/1-574 Frizzled-7 OS=Homo sapiens GN=FZD7 PE=2 SV=2

>sp|Q13467|FZD5_HUMAN/1-585 Frizzled-5 OS=Homo sapiens GN=FZD5 PE=2 SV=2

>sp|Q9H461|FZD8_HUMAN/1-694 Frizzled-8 OS=Homo sapiens GN=FZD8 PE=1 SV=1

Group 3:

>sp|O75899|GABR2_HUMAN/1-941 Gamma-aminobutyric acid type B receptor subunit 2 OS=Homo sapiens GN=GABBR2 PE=1 SV=1

>sp|Q86SQ4|GP126_HUMAN/1-1221 G-protein coupled receptor 126 OS=Homo sapiens GN=GPR126 PE=1 SV=3

>sp|O75473|LGR5_HUMAN/1-907 Leucine-rich repeat-containing G-protein coupled receptor 5 OS=Homo sapiens GN=LGR5 PE=2 SV=1

>sp|Q5T601|GP110_HUMAN/1-910 Probable G-protein coupled receptor 110 OS=Homo sapiens GN=GPR110 PE=2 SV=2

>sp|Q8IZF2|GP116_HUMAN/1-1346 Probable G-protein coupled receptor 116 OS=Homo sapiens GN=GPR116 PE=1 SV=3

>sp|Q13255|GRM1_HUMAN/1-1194 Metabotropic glutamate receptor 1 OS=Homo sapiens GN=GRM1 PE=1 SV=3

>sp|P41594|GRM5_HUMAN/1-1212 Metabotropic glutamate receptor 5 OS=Homo sapiens GN=GRM5 PE=1 SV=2

>sp|O15303|GRM6_HUMAN/1-877 Metabotropic glutamate receptor 6 OS=Homo sapiens GN=GRM6 PE=1 SV=2

>sp|Q14831|GRM7_HUMAN/1-915 Metabotropic glutamate receptor 7 OS=Homo sapiens GN=GRM7 PE=1 SV=1

>sp|Q14833|GRM4_HUMAN/1-912 Metabotropic glutamate receptor 4 OS=Homo sapiens GN=GRM4 PE=1 SV=1

>sp|O00222|GRM8_HUMAN/1-908 Metabotropic glutamate receptor 8 OS=Homo sapiens GN=GRM8 PE=2 SV=2

>sp|Q14416|GRM2_HUMAN/1-872 Metabotropic glutamate receptor 2 OS=Homo sapiens GN=GRM2 PE=2 SV=2

>sp|Q14832|GRM3_HUMAN/1-879 Metabotropic glutamate receptor 3 OS=Homo sapiens GN=GRM3 PE=1 SV=2

>sp|Q7Z7M1|GP144_HUMAN/1-963 Probable G-protein coupled receptor 144 OS=Homo sapiens GN=GPR144 PE=2 SV=1

>sp|Q9UBS5|GABR1_HUMAN/1-961 Gamma-aminobutyric acid type B receptor subunit 1 OS=Homo sapiens GN=GABBR1 PE=1 SV=1

>sp|P41180|CASR_HUMAN/1-1078 Extracellular calcium-sensing receptor OS=Homo sapiens GN=CASR PE=1 SV=2

>sp|Q5T848|GP158_HUMAN/1-1215 Probable G-protein coupled receptor 158 OS=Homo sapiens GN=GPR158 PE=1 SV=1

>sp|Q5T6X5|GPC6A_HUMAN/1-926 G-protein coupled receptor family C group 6 member A OS=Homo sapiens GN=GPRC6A PE=1 SV=1

>sp|Q14246|EMR1_HUMAN/1-886 EGF-like module-containing mucin-like hormone receptor-like 1 OS=Homo sapiens GN=EMR1 PE=2 SV=3

Group 4a:

>sp|Q86V85|GP180_HUMAN/1-440 Integral membrane protein GPR180 OS=Homo sapiens GN=GPR180 PE=2 SV=1

>sp|Q8IZF4|GP114_HUMAN/1-528 Probable G-protein coupled receptor 114 OS=Homo sapiens GN=GPR114 PE=2 SV=3

>sp|Q86Y34|GPR97_HUMAN/1-549 Probable G-protein coupled receptor 97 OS=Homo sapiens GN=GPR97 PE=1 SV=1

>sp|Q8IZF7|GP111_HUMAN/1-708 Probable G-protein coupled receptor 111 OS=Homo sapiens GN=GPR111 PE=2 SV=1

>sp|Q8IZF3|GP115_HUMAN/1-695 Probable G-protein coupled receptor 115 OS=Homo sapiens GN=GPR115 PE=2 SV=3

>sp|Q9NQ84|GPC5C_HUMAN/1-441 G-protein coupled receptor family C group 5 member C OS=Homo sapiens GN=GPRC5C PE=1 SV=2

>sp|Q7RTX0|TS1R3_HUMAN/1-852 Taste receptor type 1 member 3 OS=Homo sapiens GN=TAS1R3 PE=1 SV=2

>sp|Q99835|SMO_HUMAN/1-787 Smoothened homolog OS=Homo sapiens GN=SMO PE=1 SV=1

>sp|Q7RTX1|TS1R1_HUMAN/1-841 Taste receptor type 1 member 1 OS=Homo sapiens GN=TAS1R1 PE=2 SV=1

>sp|Q96K78|GP128_HUMAN/1-797 Probable G-protein coupled receptor 128 OS=Homo sapiens GN=GPR128 PE=1 SV=2

>sp|Q86SQ3|EMR4_HUMAN/1-457 Putative EGF-like module-containing mucin-like hormone receptor-like 4 OS=Homo sapiens GN=EMR4P PE=5 SV=1

>sp|Q9BY15|EMR3_HUMAN/1-652 EGF-like module-containing mucin-like hormone receptor-like 3 OS=Homo sapiens GN=EMR3 PE=1 SV=2

>sp|Q8NFN8|GP156_HUMAN/1-814 Probable G-protein coupled receptor 156 OS=Homo sapiens GN=GPR156 PE=2 SV=2

>sp|Q8IZF5|GP113_HUMAN/1-1079 Probable G-protein coupled receptor 113 OS=Homo sapiens GN=GPR113 PE=2 SV=1

>sp|Q86SP6|GP149_HUMAN/1-731 Probable G-protein coupled receptor 149 OS=Homo sapiens GN=GPR149 PE=2 SV=2

Group 4b:

>sp|P30988|CALCR_HUMAN/1-490 Calcitonin receptor OS=Homo sapiens GN=CALCR PE=1 SV=1

>sp|Q16602|CALRL_HUMAN/1-461 Calcitonin gene-related peptide type 1 receptor OS=Homo sapiens GN=CALCRL PE=1 SV=2

>sp|P34998|CRFR1_HUMAN/1-444 Corticotropin-releasing factor receptor 1 OS=Homo sapiens GN=CRHR1 PE=1 SV=1

>sp|Q13324|CRFR2_HUMAN/1-411 Corticotropin-releasing factor receptor 2 OS=Homo sapiens GN=CRHR2 PE=1 SV=2

>sp|Q02643|GHRHR_HUMAN/1-423 Growth hormone-releasing hormone receptor OS=Homo sapiens GN=GHRHR PE=1 SV=2

>sp|O95838|GLP2R_HUMAN/1-553 Glucagon-like peptide 2 receptor OS=Homo sapiens GN=GLP2R PE=1 SV=1

>sp|P43220|GLP1R_HUMAN/1-463 Glucagon-like peptide 1 receptor OS=Homo sapiens GN=GLP1R PE=1 SV=2

>sp|P48546|GIPR_HUMAN/1-466 Gastric inhibitory polypeptide receptor OS=Homo sapiens GN=GIPR PE=1 SV=1

>sp|P47871|GLR_HUMAN/1-477 Glucagon receptor OS=Homo sapiens GN=GCGR PE=1 SV=1

>sp|Q03431|PTH1R_HUMAN/1-593 Parathyroid hormone/parathyroid hormone-related peptide receptor OS=Homo sapiens GN=PTH1R PE=1 SV=1

>sp|P49190|PTH2R_HUMAN/1-550 Parathyroid hormone 2 receptor OS=Homo sapiens GN=PTH2R PE=1 SV=1

>sp|P47872|SCTR_HUMAN/1-440 Secretin receptor OS=Homo sapiens GN=SCTR PE=2 SV=2

>sp|P32241|VIPR1_HUMAN/1-457 Vasoactive intestinal polypeptide receptor 1 OS=Homo sapiens GN=VIPR1 PE=1 SV=1

>sp|P41587|VIPR2_HUMAN/1-438 Vasoactive intestinal polypeptide receptor 2 OS=Homo sapiens GN=VIPR2 PE=1 SV=2

>sp|P41586|PACR_HUMAN/1-468 Pituitary adenylate cyclase-activating polypeptide type I receptor OS=Homo sapiens GN=ADCYAP1R1 PE=1 SV=1

Group 5a:

>sp|P59533|T2R38_HUMAN/1-333 Taste receptor type 2 member 38 OS=Homo sapiens GN=TAS2R38 PE=2 SV=3

>sp|Q9NYV7|T2R16_HUMAN/1-291 Taste receptor type 2 member 16 OS=Homo sapiens GN=TAS2R16 PE=1 SV=1

>sp|P59534|T2R39_HUMAN/1-338 Taste receptor type 2 member 39 OS=Homo sapiens GN=TAS2R39 PE=2 SV=3

>sp|P59535|T2R40_HUMAN/1-323 Taste receptor type 2 member 40 OS=Homo sapiens GN=TAS2R40 PE=2 SV=1

>sp|P59551|T2R60_HUMAN/1-318 Taste receptor type 2 member 60 OS=Homo sapiens GN=TAS2R60 PE=1 SV=1

>sp|P59536|T2R41_HUMAN/1-307 Taste receptor type 2 member 41 OS=Homo sapiens GN=TAS2R41 PE=2 SV=2

>sp|Q9NYW7|TA2R1_HUMAN/1-299 Taste receptor type 2 member 1 OS=Homo sapiens GN=TAS2R1 PE=1 SV=1

>sp|P59544|T2R50_HUMAN/1-299 Taste receptor type 2 member 50 OS=Homo sapiens GN=TAS2R50 PE=2 SV=2

>sp|P59541|T2R30_HUMAN/1-319 Taste receptor type 2 member 30 OS=Homo sapiens GN=TAS2R30 PE=2 SV=2

>sp|P59539|T2R45_HUMAN/1-299 Taste receptor type 2 member 45 OS=Homo sapiens GN=TAS2R45 PE=2 SV=1

>sp|P59540|T2R46_HUMAN/1-309 Taste receptor type 2 member 46 OS=Homo sapiens GN=TAS2R46 PE=2 SV=2

>sp|P59538|T2R31_HUMAN/1-309 Taste receptor type 2 member 31 OS=Homo sapiens GN=TAS2R31 PE=2 SV=2

>sp|P59537|T2R43_HUMAN/1-309 Taste receptor type 2 member 43 OS=Homo sapiens GN=TAS2R43 PE=2 SV=2

>sp|P59542|T2R19_HUMAN/1-299 Taste receptor type 2 member 19 OS=Homo sapiens GN=TAS2R19 PE=1 SV=1

>sp|P59543|T2R20_HUMAN/1-309 Taste receptor type 2 member 20 OS=Homo sapiens GN=TAS2R20 PE=2 SV=2

>sp|Q9NYV9|T2R13_HUMAN/1-303 Taste receptor type 2 member 13 OS=Homo sapiens GN=TAS2R13 PE=1 SV=1

>sp|Q9NYV8|T2R14_HUMAN/1-317 Taste receptor type 2 member 14 OS=Homo sapiens GN=TAS2R14 PE=1 SV=1

>sp|Q9NYW6|TA2R3_HUMAN/1-316 Taste receptor type 2 member 3 OS=Homo sapiens GN=TAS2R3 PE=1 SV=3

>sp|Q9NYW0|T2R10_HUMAN/1-307 Taste receptor type 2 member 10 OS=Homo sapiens GN=TAS2R10 PE=1 SV=3

>sp|Q7RTR8|T2R42_HUMAN/1-314 Taste receptor type 2 member 42 OS=Homo sapiens GN=TAS2R42 PE=2 SV=1

>sp|P59531|T2R12_HUMAN/1-264 Putative taste receptor type 2 member 12 OS=Homo sapiens GN=TAS2R12 PE=5 SV=1

>sp|Q9NYW2|TA2R8_HUMAN/1-309 Taste receptor type 2 member 8 OS=Homo sapiens GN=TAS2R8 PE=1 SV=1

>sp|Q9NYW3|TA2R7_HUMAN/1-318 Taste receptor type 2 member 7 OS=Homo sapiens GN=TAS2R7 PE=1 SV=1

>sp|Q9NYW1|TA2R9_HUMAN/1-312 Taste receptor type 2 member 9 OS=Homo sapiens GN=TAS2R9 PE=1 SV=1

>sp|Q9NYW5|TA2R4_HUMAN/1-299 Taste receptor type 2 member 4 OS=Homo sapiens GN=TAS2R4 PE=1 SV=1

>sp|Q9NYW4|TA2R5_HUMAN/1-299 Taste receptor type 2 member 5 OS=Homo sapiens GN=TAS2R5 PE=1 SV=1

Group 5b:

>sp|Q86SQ6|GP123_HUMAN/1-560 Probable G-protein coupled receptor 123 OS=Homo sapiens GN=GPR123 PE=2 SV=3

>sp|Q9HBW9|ELTD1_HUMAN/1-690 EGF, latrophilin and seven transmembrane domain-containing protein 1 OS=Homo sapiens GN=ELTD1 PE=1 SV=3

>sp|Q9Y653|GPR56_HUMAN/1-693 G-protein coupled receptor 56 OS=Homo sapiens GN=GPR56 PE=1 SV=2

>sp|Q8TE23|TS1R2_HUMAN/1-839 Taste receptor type 1 member 2 OS=Homo sapiens GN=TAS1R2 PE=2 SV=2

>sp|Q6QNK2|GP133_HUMAN/1-874 Probable G-protein coupled receptor 133 OS=Homo sapiens GN=GPR133 PE=2 SV=1

Group 6a:

>sp|P48960|CD97_HUMAN/1-835 CD97 antigen OS=Homo sapiens GN=CD97 PE=1 SV=4

>sp|Q9UHX3|EMR2_HUMAN/1-823 EGF-like module-containing mucin-like hormone receptor-like 2 OS=Homo sapiens GN=EMR2 PE=1 SV=2

>sp|P16473|TSHR_HUMAN/1-764 Thyrotropin receptor OS=Homo sapiens GN=TSHR PE=1 SV=2

>sp|P23945|FSHR_HUMAN/1-695 Follicle-stimulating hormone receptor OS=Homo sapiens GN=FSHR PE=1 SV=3

>sp|P22888|LSHR_HUMAN/1-699 Lutropin-choriogonadotropic hormone receptor OS=Homo sapiens GN=LHCGR PE=1 SV=4

>sp|O60883|ETBR2_HUMAN/1-481 Endothelin B receptor-like protein 2 OS=Homo sapiens GN=GPR37L1 PE=1 SV=2

>sp|O15354|GPR37_HUMAN/1-613 Probable G-protein coupled receptor 37 OS=Homo sapiens GN=GPR37 PE=1 SV=2

>sp|Q9HBX9|RXFP1_HUMAN/1-757 Relaxin receptor 1 OS=Homo sapiens GN=RXFP1 PE=1 SV=2

>sp|Q8WXD0|RXFP2_HUMAN/1-754 Relaxin receptor 2 OS=Homo sapiens GN=RXFP2 PE=1 SV=1

>sp|Q7Z601|GP142_HUMAN/1-462 Probable G-protein coupled receptor 142 OS=Homo sapiens GN=GPR142 PE=2 SV=1

Group 6b:

>sp|Q9GZP7|VN1R1_HUMAN/1-353 Vomeronasal type-1 receptor 1 OS=Homo sapiens GN=VN1R1 PE=2 SV=1

>sp|Q9BXE9|VN1R3_HUMAN/1-311 Vomeronasal type-1 receptor 3 OS=Homo sapiens GN=VN1R3 PE=2 SV=1

>sp|Q7Z5H5|VN1R4_HUMAN/1-301 Vomeronasal type-1 receptor 4 OS=Homo sapiens GN=VN1R4 PE=2 SV=2

>sp|Q8NFZ6|VN1R2_HUMAN/1-395 Vomeronasal type-1 receptor 2 OS=Homo sapiens GN=VN1R2 PE=2 SV=2

Group 6c:

>sp|Q01726|MSHR_HUMAN/1-317 Melanocyte-stimulating hormone receptor OS=Homo sapiens GN=MC1R PE=1 SV=2

>sp|Q01718|ACTHR_HUMAN/1-297 Adrenocorticotropic hormone receptor OS=Homo sapiens GN=MC2R PE=1 SV=1

>sp|P32245|MC4R_HUMAN/1-332 Melanocortin receptor 4 OS=Homo sapiens GN=MC4R PE=1 SV=2

>sp|P41968|MC3R_HUMAN/1-360 Melanocortin receptor 3 OS=Homo sapiens GN=MC3R PE=1 SV=2

>sp|P33032|MC5R_HUMAN/1-325 Melanocortin receptor 5 OS=Homo sapiens GN=MC5R PE=1 SV=3

Group 7:

>sp|A6NND4|O2AT4_HUMAN/1-320 Olfactory receptor 2AT4 OS=Homo sapiens GN=OR2AT4 PE=2 SV=1

>sp|Q8NHC7|O14CZ_HUMAN/1-312 Olfactory receptor 14C36 OS=Homo sapiens GN=OR14C36 PE=2 SV=1

>sp|Q8NGZ2|O14K1_HUMAN/1-314 Olfactory receptor 14K1 OS=Homo sapiens GN=OR14K1 PE=2 SV=2

>sp|Q9UGF5|O14J1_HUMAN/1-321 Olfactory receptor 14J1 OS=Homo sapiens GN=OR14J1 PE=2 SV=1

>sp|Q8NHC6|O14L1_HUMAN/1-308 Putative olfactory receptor 14L1 OS=Homo sapiens GN=OR14L1P PE=5 SV=1

>sp|A6ND48|O14I1_HUMAN/1-311 Olfactory receptor 14I1 OS=Homo sapiens GN=OR14I1 PE=2 SV=1

>sp|Q96R54|O14A2_HUMAN/1-314 Olfactory receptor 14A2 OS=Homo sapiens GN=OR14A2 PE=2 SV=2

>sp|Q8NHC5|O14AG_HUMAN/1-309 Olfactory receptor 14A16 OS=Homo sapiens GN=OR14A16 PE=2 SV=1

>sp|A6NFC9|OR2W5_HUMAN/1-320 Putative olfactory receptor 2W5 OS=Homo sapiens GN=OR2W5 PE=5 SV=1

>sp|P58182|O12D2_HUMAN/1-307 Olfactory receptor 12D2 OS=Homo sapiens GN=OR12D2 PE=2 SV=2

>sp|Q9UGF7|O12D3_HUMAN/1-316 Olfactory receptor 12D3 OS=Homo sapiens GN=OR12D3 PE=2 SV=1

>sp|Q8N148|OR6V1_HUMAN/1-313 Olfactory receptor 6V1 OS=Homo sapiens GN=OR6V1 PE=2 SV=1

>sp|Q9GZK7|O11A1_HUMAN/1-315 Olfactory receptor 11A1 OS=Homo sapiens GN=OR11A1 PE=2 SV=1

>sp|Q8NGU1|OR9A1_HUMAN/1-263 Putative olfactory receptor 9A1 OS=Homo sapiens GN=OR9A1P PE=5 SV=2

>sp|Q8NGT5|OR9A2_HUMAN/1-310 Olfactory receptor 9A2 OS=Homo sapiens GN=OR9A2 PE=2 SV=1

>sp|Q8NGU2|OR9A4_HUMAN/1-314 Olfactory receptor 9A4 OS=Homo sapiens GN=OR9A4 PE=2 SV=1

>sp|Q6IEV9|OR4CB_HUMAN/1-310 Olfactory receptor 4C11 OS=Homo sapiens GN=OR4C11 PE=2 SV=1

>sp|Q8NGL9|OR4CG_HUMAN/1-310 Olfactory receptor 4C16 OS=Homo sapiens GN=OR4C16 PE=2 SV=2

>sp|Q8NGL6|O4A15_HUMAN/1-344 Olfactory receptor 4A15 OS=Homo sapiens GN=OR4A15 PE=2 SV=3

>sp|Q6IF82|O4A47_HUMAN/1-309 Olfactory receptor 4A47 OS=Homo sapiens GN=OR4A47 PE=2 SV=2

>sp|Q8NGN8|OR4A4_HUMAN/1-299 Putative olfactory receptor 4A4 OS=Homo sapiens GN=OR4A4P PE=5 SV=1

>sp|Q8NH70|O4A16_HUMAN/1-328 Olfactory receptor 4A16 OS=Homo sapiens GN=OR4A16 PE=2 SV=1

>sp|Q8NH83|OR4A5_HUMAN/1-315 Olfactory receptor 4A5 OS=Homo sapiens GN=OR4A5 PE=2 SV=4

>sp|P0C604|OR4A8_HUMAN/1-315 Putative olfactory receptor 4A8 OS=Homo sapiens GN=OR4A8P PE=5 SV=1

>sp|A6NMZ5|O4C45_HUMAN/1-311 Olfactory receptor 4C45 OS=Homo sapiens GN=OR4C45 PE=2 SV=1

>sp|Q8NH72|OR4C6_HUMAN/1-309 Olfactory receptor 4C6 OS=Homo sapiens GN=OR4C6 PE=2 SV=1

>sp|Q8NH37|OR4C3_HUMAN/1-302 Olfactory receptor 4C3 OS=Homo sapiens GN=OR4C3 PE=2 SV=2

>sp|Q8NGB2|OR4C5_HUMAN/1-326 Olfactory receptor 4C5 OS=Homo sapiens GN=OR4C5 PE=2 SV=1

>sp|Q8NGM1|OR4CF_HUMAN/1-316 Olfactory receptor 4C15 OS=Homo sapiens GN=OR4C15 PE=2 SV=1

>sp|Q96R67|OR4CC_HUMAN/1-309 Olfactory receptor 4C12 OS=Homo sapiens GN=OR4C12 PE=2 SV=2

>sp|A6NHA9|O4C46_HUMAN/1-309 Olfactory receptor 4C46 OS=Homo sapiens GN=OR4C46 PE=2 SV=1

>sp|Q8NGP0|OR4CD_HUMAN/1-309 Olfactory receptor 4C13 OS=Homo sapiens GN=OR4C13 PE=2 SV=2

>sp|Q8NGB4|OR4S1_HUMAN/1-309 Olfactory receptor 4S1 OS=Homo sapiens GN=OR4S1 PE=2 SV=1

>sp|Q8NGL7|OR4P4_HUMAN/1-312 Olfactory receptor 4P4 OS=Homo sapiens GN=OR4P4 PE=2 SV=1

>sp|Q8NH49|OR4X1_HUMAN/1-305 Olfactory receptor 4X1 OS=Homo sapiens GN=OR4X1 PE=2 SV=1

>sp|Q8NGF9|OR4X2_HUMAN/1-303 Olfactory receptor 4X2 OS=Homo sapiens GN=OR4X2 PE=2 SV=1

>sp|Q8NGF8|OR4B1_HUMAN/1-309 Olfactory receptor 4B1 OS=Homo sapiens GN=OR4B1 PE=2 SV=1

>sp|Q8NH73|OR4S2_HUMAN/1-311 Olfactory receptor 4S2 OS=Homo sapiens GN=OR4S2 PE=1 SV=2

>sp|Q32VQ0|OR4FL_HUMAN/1-273 Putative olfactory receptor GPCRLTM7 OS=Homo sapiens PE=5 SV=1

>sp|Q8NGB9|OR4F6_HUMAN/1-312 Olfactory receptor 4F6 OS=Homo sapiens GN=OR4F6 PE=2 SV=1

>sp|Q8NGB8|O4F15_HUMAN/1-312 Olfactory receptor 4F15 OS=Homo sapiens GN=OR4F15 PE=2 SV=1

>sp|O95013|O4F21_HUMAN/1-312 Olfactory receptor 4F21 OS=Homo sapiens GN=OR4F21 PE=2 SV=2

>sp|Q6IEY1|OR4F3_HUMAN/1-312 Olfactory receptor 4F3/4F16/4F29 OS=Homo sapiens GN=OR4F3 PE=2 SV=1

>sp|Q96R69|OR4F4_HUMAN/1-305 Olfactory receptor 4F4 OS=Homo sapiens GN=OR4F4 PE=2 SV=2

>sp|Q8NH21|OR4F5_HUMAN/1-305 Olfactory receptor 4F5 OS=Homo sapiens GN=OR4F5 PE=2 SV=1

>sp|Q8NGA8|O4F17_HUMAN/1-305 Olfactory receptor 4F17 OS=Homo sapiens GN=OR4F17 PE=2 SV=1

>sp|Q8NGC6|OR4KH_HUMAN/1-315 Olfactory receptor 4K17 OS=Homo sapiens GN=OR4K17 PE=2 SV=3

>sp|Q8NH43|OR4L1_HUMAN/1-312 Olfactory receptor 4L1 OS=Homo sapiens GN=OR4L1 PE=2 SV=1

>sp|Q8NGD4|OR4K1_HUMAN/1-311 Olfactory receptor 4K1 OS=Homo sapiens GN=OR4K1 PE=2 SV=1

>sp|Q8NGD2|OR4K2_HUMAN/1-314 Olfactory receptor 4K2 OS=Homo sapiens GN=OR4K2 PE=2 SV=1

>sp|Q96R72|OR4K3_HUMAN/1-315 Olfactory receptor 4K3 OS=Homo sapiens GN=OR4K3 PE=3 SV=3

>sp|Q8NH42|OR4KD_HUMAN/1-304 Olfactory receptor 4K13 OS=Homo sapiens GN=OR4K13 PE=2 SV=1

>sp|Q8NGD5|OR4KE_HUMAN/1-310 Olfactory receptor 4K14 OS=Homo sapiens GN=OR4K14 PE=2 SV=1

>sp|Q8NGD3|OR4K5_HUMAN/1-323 Olfactory receptor 4K5 OS=Homo sapiens GN=OR4K5 PE=2 SV=1

>sp|Q8NH41|OR4KF_HUMAN/1-348 Olfactory receptor 4K15 OS=Homo sapiens GN=OR4K15 PE=2 SV=2

>sp|Q8NGC2|OR4E2_HUMAN/1-313 Olfactory receptor 4E2 OS=Homo sapiens GN=OR4E2 PE=2 SV=1

>sp|P0C645|OR4E1_HUMAN/1-316 Olfactory receptor 4E1 OS=Homo sapiens GN=OR4E1 PE=3 SV=1

>sp|Q8NGD0|OR4M1_HUMAN/1-313 Olfactory receptor 4M1 OS=Homo sapiens GN=OR4M1 PE=2 SV=1

>sp|Q8NGB6|OR4M2_HUMAN/1-313 Olfactory receptor 4M2 OS=Homo sapiens GN=OR4M2 PE=2 SV=2

>sp|Q8IXE1|OR4N5_HUMAN/1-308 Olfactory receptor 4N5 OS=Homo sapiens GN=OR4N5 PE=2 SV=1

>sp|Q8NGD1|OR4N2_HUMAN/1-307 Olfactory receptor 4N2 OS=Homo sapiens GN=OR4N2 PE=2 SV=1

>sp|Q8N0Y3|OR4N4_HUMAN/1-316 Olfactory receptor 4N4 OS=Homo sapiens GN=OR4N4 PE=2 SV=2

>sp|Q8NGN0|OR4D5_HUMAN/1-318 Olfactory receptor 4D5 OS=Homo sapiens GN=OR4D5 PE=2 SV=1

>sp|Q15615|OR4D1_HUMAN/1-310 Olfactory receptor 4D1 OS=Homo sapiens GN=OR4D1 PE=2 SV=3

>sp|P58180|OR4D2_HUMAN/1-307 Olfactory receptor 4D2 OS=Homo sapiens GN=OR4D2 PE=2 SV=1

>sp|Q8NGJ1|OR4D6_HUMAN/1-314 Olfactory receptor 4D6 OS=Homo sapiens GN=OR4D6 PE=2 SV=1

>sp|Q8NGI6|OR4DA_HUMAN/1-311 Olfactory receptor 4D10 OS=Homo sapiens GN=OR4D10 PE=2 SV=1

>sp|Q8NGE8|OR4D9_HUMAN/1-314 Olfactory receptor 4D9 OS=Homo sapiens GN=OR4D9 PE=2 SV=3

>sp|Q8NGI4|OR4DB_HUMAN/1-311 Olfactory receptor 4D11 OS=Homo sapiens GN=OR4D11 PE=2 SV=1

>sp|Q8NH05|OR4Q3_HUMAN/1-313 Olfactory receptor 4Q3 OS=Homo sapiens GN=OR4Q3 PE=2 SV=1

>sp|P0C623|OR4Q2_HUMAN/1-313 Olfactory receptor 4Q2 OS=Homo sapiens GN=OR4Q2 PE=3 SV=1

>sp|Q8NH80|O10D3_HUMAN/1-312 Putative olfactory receptor 10D3 OS=Homo sapiens GN=OR10D3 PE=5 SV=1

>sp|Q8NGN7|O10D4_HUMAN/1-298 Putative olfactory receptor 10D4 OS=Homo sapiens GN=OR10D4P PE=5 SV=1

>sp|Q8NGN2|O10S1_HUMAN/1-331 Olfactory receptor 10S1 OS=Homo sapiens GN=OR10S1 PE=2 SV=2

>sp|Q8NGN5|O10G8_HUMAN/1-311 Olfactory receptor 10G8 OS=Homo sapiens GN=OR10G8 PE=2 SV=1

>sp|Q8NGN6|O10G7_HUMAN/1-311 Olfactory receptor 10G7 OS=Homo sapiens GN=OR10G7 PE=2 SV=1

>sp|Q8NGN3|O10G4_HUMAN/1-311 Olfactory receptor 10G4 OS=Homo sapiens GN=OR10G4 PE=2 SV=1

>sp|Q8NGN4|O10G9_HUMAN/1-311 Olfactory receptor 10G9 OS=Homo sapiens GN=OR10G9 PE=2 SV=1

>sp|Q8NH81|O10G6_HUMAN/1-332 Olfactory receptor 10G6 OS=Homo sapiens GN=OR10G6 PE=2 SV=1

>sp|Q8NGC3|O10G2_HUMAN/1-310 Olfactory receptor 10G2 OS=Homo sapiens GN=OR10G2 PE=2 SV=1

>sp|Q8NGC4|O10G3_HUMAN/1-313 Olfactory receptor 10G3 OS=Homo sapiens GN=OR10G3 PE=2 SV=1

>sp|Q8NH79|OR6X1_HUMAN/1-312 Olfactory receptor 6X1 OS=Homo sapiens GN=OR6X1 PE=2 SV=1

>sp|Q8NGQ2|OR6Q1_HUMAN/1-317 Olfactory receptor 6Q1 OS=Homo sapiens GN=OR6Q1 PE=2 SV=2

>sp|A6NDL8|O6C68_HUMAN/1-312 Olfactory receptor 6C68 OS=Homo sapiens GN=OR6C68 PE=2 SV=2

>sp|Q9NZP2|OR6C2_HUMAN/1-312 Olfactory receptor 6C2 OS=Homo sapiens GN=OR6C2 PE=2 SV=2

>sp|Q8NGE2|O2AP1_HUMAN/1-309 Olfactory receptor 2AP1 OS=Homo sapiens GN=OR2AP1 PE=2 SV=1

>sp|Q8NGE1|OR6C4_HUMAN/1-309 Olfactory receptor 6C4 OS=Homo sapiens GN=OR6C4 PE=2 SV=1

>sp|A6NIJ9|O6C70_HUMAN/1-312 Olfactory receptor 6C70 OS=Homo sapiens GN=OR6C70 PE=2 SV=1

>sp|A6NF89|OR6C6_HUMAN/1-314 Olfactory receptor 6C6 OS=Homo sapiens GN=OR6C6 PE=2 SV=1

>sp|Q96RD1|OR6C1_HUMAN/1-312 Olfactory receptor 6C1 OS=Homo sapiens GN=OR6C1 PE=2 SV=2

>sp|Q9NZP0|OR6C3_HUMAN/1-311 Olfactory receptor 6C3 OS=Homo sapiens GN=OR6C3 PE=2 SV=2

>sp|A6NJZ3|O6C65_HUMAN/1-312 Olfactory receptor 6C65 OS=Homo sapiens GN=OR6C65 PE=2 SV=1

>sp|A6NM76|O6C76_HUMAN/1-312 Olfactory receptor 6C76 OS=Homo sapiens GN=OR6C76 PE=2 SV=1

>sp|A6NCV1|O6C74_HUMAN/1-312 Olfactory receptor 6C74 OS=Homo sapiens GN=OR6C74 PE=2 SV=1

>sp|A6NL08|O6C75_HUMAN/1-312 Olfactory receptor 6C75 OS=Homo sapiens GN=OR6C75 PE=2 SV=1

>sp|Q6IFH4|OR6B2_HUMAN/1-312 Olfactory receptor 6B2 OS=Homo sapiens GN=OR6B2 PE=2 SV=2

>sp|Q8NGW1|OR6B3_HUMAN/1-331 Olfactory receptor 6B3 OS=Homo sapiens GN=OR6B3 PE=2 SV=1

>sp|O95007|OR6B1_HUMAN/1-311 Olfactory receptor 6B1 OS=Homo sapiens GN=OR6B1 PE=2 SV=1

>sp|O95222|OR6A2_HUMAN/1-327 Olfactory receptor 6A2 OS=Homo sapiens GN=OR6A2 PE=2 SV=2

>sp|Q8NGX8|OR6Y1_HUMAN/1-325 Olfactory receptor 6Y1 OS=Homo sapiens GN=OR6Y1 PE=2 SV=1

>sp|Q8NGX9|OR6P1_HUMAN/1-317 Olfactory receptor 6P1 OS=Homo sapiens GN=OR6P1 PE=2 SV=1

>sp|Q8NH40|OR6S1_HUMAN/1-331 Olfactory receptor 6S1 OS=Homo sapiens GN=OR6S1 PE=2 SV=2

>sp|Q8NGN1|OR6T1_HUMAN/1-323 Olfactory receptor 6T1 OS=Homo sapiens GN=OR6T1 PE=2 SV=1

>sp|Q8NGC5|OR6J1_HUMAN/1-347 Olfactory receptor 6J1 OS=Homo sapiens GN=OR6J1 PE=2 SV=1

>sp|Q8NGM8|OR6M1_HUMAN/1-313 Olfactory receptor 6M1 OS=Homo sapiens GN=OR6M1 PE=2 SV=1

>sp|Q8NGZ6|OR6F1_HUMAN/1-308 Olfactory receptor 6F1 OS=Homo sapiens GN=OR6F1 PE=2 SV=1

>sp|Q8NGY5|OR6N1_HUMAN/1-312 Olfactory receptor 6N1 OS=Homo sapiens GN=OR6N1 PE=2 SV=1

>sp|Q8NGY6|OR6N2_HUMAN/1-317 Olfactory receptor 6N2 OS=Homo sapiens GN=OR6N2 PE=1 SV=1

>sp|Q8NGY2|OR6K2_HUMAN/1-324 Olfactory receptor 6K2 OS=Homo sapiens GN=OR6K2 PE=2 SV=1

>sp|Q8NGY3|OR6K3_HUMAN/1-331 Olfactory receptor 6K3 OS=Homo sapiens GN=OR6K3 PE=2 SV=2

>sp|Q8NGW6|OR6K6_HUMAN/1-343 Olfactory receptor 6K6 OS=Homo sapiens GN=OR6K6 PE=2 SV=2

>sp|Q8NGX0|O11L1_HUMAN/1-322 Olfactory receptor 11L1 OS=Homo sapiens GN=OR11L1 PE=2 SV=1

>sp|Q8NGC1|O11G2_HUMAN/1-345 Olfactory receptor 11G2 OS=Homo sapiens GN=OR11G2 PE=2 SV=2

>sp|Q8NH07|O11H2_HUMAN/1-326 Olfactory receptor 11H2 OS=Homo sapiens GN=OR11H2 PE=3 SV=1

>sp|Q8NG94|O11H1_HUMAN/1-326 Olfactory receptor 11H1 OS=Homo sapiens GN=OR11H1 PE=2 SV=3

>sp|B2RN74|O11HC_HUMAN/1-326 Olfactory receptor 11H12 OS=Homo sapiens GN=OR11H12 PE=2 SV=1

>sp|Q8NGC7|O11H6_HUMAN/1-330 Olfactory receptor 11H6 OS=Homo sapiens GN=OR11H6 PE=2 SV=1

>sp|Q8NGC9|O11H4_HUMAN/1-324 Olfactory receptor 11H4 OS=Homo sapiens GN=OR11H4 PE=2 SV=1

>sp|Q8NGC8|O11H7_HUMAN/1-314 Olfactory receptor 11H7 OS=Homo sapiens GN=OR11H7 PE=2 SV=2

>sp|Q8NGR1|O13A1_HUMAN/1-328 Olfactory receptor 13A1 OS=Homo sapiens GN=OR13A1 PE=2 SV=2

>sp|Q8NGZ3|O13G1_HUMAN/1-307 Olfactory receptor 13G1 OS=Homo sapiens GN=OR13G1 PE=2 SV=1

>sp|Q8NGE0|O10AD_HUMAN/1-317 Olfactory receptor 10AD1 OS=Homo sapiens GN=OR10AD1 PE=2 SV=1

>sp|Q8NH08|O10AC_HUMAN/1-325 Putative olfactory receptor 10AC1 OS=Homo sapiens GN=OR10AC1P PE=3 SV=2

>sp|Q9H205|O2AG1_HUMAN/1-316 Olfactory receptor 2AG1 OS=Homo sapiens GN=OR2AG1 PE=2 SV=2

>sp|A6NM03|O2AG2_HUMAN/1-316 Olfactory receptor 2AG2 OS=Homo sapiens GN=OR2AG2 PE=2 SV=1

>sp|Q8NHA4|O2AE1_HUMAN/1-323 Olfactory receptor 2AE1 OS=Homo sapiens GN=OR2AE1 PE=2 SV=1

>sp|Q8NG97|OR2Z1_HUMAN/1-314 Olfactory receptor 2Z1 OS=Homo sapiens GN=OR2Z1 PE=2 SV=1

>sp|Q8NH01|O2T11_HUMAN/1-316 Olfactory receptor 2T11 OS=Homo sapiens GN=OR2T11 PE=2 SV=1

>sp|Q8NGX2|O2T35_HUMAN/1-323 Olfactory receptor 2T35 OS=Homo sapiens GN=OR2T35 PE=2 SV=1

>sp|Q6IF00|OR2T2_HUMAN/1-324 Olfactory receptor 2T2 OS=Homo sapiens GN=OR2T2 PE=2 SV=1

>sp|Q8NH04|O2T27_HUMAN/1-317 Olfactory receptor 2T27 OS=Homo sapiens GN=OR2T27 PE=2 SV=1

>sp|P0C7T2|OR2T7_HUMAN/1-308 Olfactory receptor 2T7 OS=Homo sapiens GN=OR2T7 PE=3 SV=1

>sp|O43869|OR2T1_HUMAN/1-369 Olfactory receptor 2T1 OS=Homo sapiens GN=OR2T1 PE=2 SV=3

>sp|Q8NHC8|OR2T6_HUMAN/1-308 Olfactory receptor 2T6 OS=Homo sapiens GN=OR2T6 PE=2 SV=2

>sp|Q8NGX1|O2T34_HUMAN/1-318 Olfactory receptor 2T34 OS=Homo sapiens GN=OR2T34 PE=2 SV=1

>sp|Q8NH03|OR2T3_HUMAN/1-318 Olfactory receptor 2T3 OS=Homo sapiens GN=OR2T3 PE=2 SV=2

>sp|Q8NGZ9|O2T10_HUMAN/1-312 Olfactory receptor 2T10 OS=Homo sapiens GN=OR2T10 PE=2 SV=1

>sp|Q8NH00|OR2T4_HUMAN/1-348 Olfactory receptor 2T4 OS=Homo sapiens GN=OR2T4 PE=2 SV=2

>sp|Q8NH02|O2T29_HUMAN/1-315 Olfactory receptor 2T29 OS=Homo sapiens GN=OR2T29 PE=2 SV=2

>sp|Q6IEZ7|OR2T5_HUMAN/1-315 Olfactory receptor 2T5 OS=Homo sapiens GN=OR2T5 PE=2 SV=1

>sp|Q8NHB1|OR2V1_HUMAN/1-315 Olfactory receptor 2V1 OS=Homo sapiens GN=OR2V1 PE=3 SV=2

>sp|Q96R30|OR2V2_HUMAN/1-315 Olfactory receptor 2V2 OS=Homo sapiens GN=OR2V2 PE=2 SV=3

>sp|A6NH00|OR2T8_HUMAN/1-312 Olfactory receptor 2T8 OS=Homo sapiens GN=OR2T8 PE=2 SV=1

>sp|Q8NG77|O2T12_HUMAN/1-320 Olfactory receptor 2T12 OS=Homo sapiens GN=OR2T12 PE=2 SV=1

>sp|Q8NG76|O2T33_HUMAN/1-320 Olfactory receptor 2T33 OS=Homo sapiens GN=OR2T33 PE=2 SV=1

>sp|Q96R27|OR2M4_HUMAN/1-311 Olfactory receptor 2M4 OS=Homo sapiens GN=OR2M4 PE=2 SV=2

>sp|Q96R28|OR2M2_HUMAN/1-347 Olfactory receptor 2M2 OS=Homo sapiens GN=OR2M2 PE=2 SV=2

>sp|Q8NG81|OR2M7_HUMAN/1-312 Olfactory receptor 2M7 OS=Homo sapiens GN=OR2M7 PE=2 SV=1

>sp|Q8NG83|OR2M3_HUMAN/1-312 Olfactory receptor 2M3 OS=Homo sapiens GN=OR2M3 PE=2 SV=1

>sp|A3KFT3|OR2M5_HUMAN/1-312 Olfactory receptor 2M5 OS=Homo sapiens GN=OR2M5 PE=2 SV=1

>sp|Q8NGZ0|O2AJ1_HUMAN/1-328 Olfactory receptor 2AJ1 OS=Homo sapiens GN=OR2AJ1 PE=3 SV=1

>sp|Q8NG84|O2AK2_HUMAN/1-335 Olfactory receptor 2AK2 OS=Homo sapiens GN=OR2AK2 PE=2 SV=2

>sp|Q8N349|OR2LD_HUMAN/1-312 Olfactory receptor 2L13 OS=Homo sapiens GN=OR2L13 PE=2 SV=1

>sp|Q8NG85|OR2L3_HUMAN/1-312 Olfactory receptor 2L3 OS=Homo sapiens GN=OR2L3 PE=2 SV=1

>sp|Q8NGY9|OR2L8_HUMAN/1-312 Olfactory receptor 2L8 OS=Homo sapiens GN=OR2L8 PE=2 SV=1

>sp|Q8NH16|OR2L2_HUMAN/1-312 Olfactory receptor 2L2 OS=Homo sapiens GN=OR2L2 PE=2 SV=1

>sp|Q8NG80|OR2L5_HUMAN/1-312 Olfactory receptor 2L5 OS=Homo sapiens GN=OR2L5 PE=3 SV=1

>sp|Q8NH09|OR8S1_HUMAN/1-359 Olfactory receptor 8S1 OS=Homo sapiens GN=OR8S1 PE=2 SV=2

>sp|Q8NGR6|OR1B1_HUMAN/1-318 Olfactory receptor 1B1 OS=Homo sapiens GN=OR1B1 PE=2 SV=2

>sp|Q15612|OR1Q1_HUMAN/1-314 Olfactory receptor 1Q1 OS=Homo sapiens GN=OR1Q1 PE=2 SV=3

>sp|Q9P1Q5|OR1A1_HUMAN/1-309 Olfactory receptor 1A1 OS=Homo sapiens GN=OR1A1 PE=2 SV=2

>sp|Q9Y585|OR1A2_HUMAN/1-309 Olfactory receptor 1A2 OS=Homo sapiens GN=OR1A2 PE=2 SV=1

>sp|Q8NGR5|OR1L4_HUMAN/1-311 Olfactory receptor 1L4 OS=Homo sapiens GN=OR1L4 PE=2 SV=1

>sp|Q8NGR2|OR1L6_HUMAN/1-347 Olfactory receptor 1L6 OS=Homo sapiens GN=OR1L6 PE=2 SV=2

>sp|Q8NGR8|OR1L8_HUMAN/1-309 Olfactory receptor 1L8 OS=Homo sapiens GN=OR1L8 PE=2 SV=1

>sp|Q8NH94|OR1L1_HUMAN/1-360 Olfactory receptor 1L1 OS=Homo sapiens GN=OR1L1 PE=2 SV=3

>sp|Q8NH93|OR1L3_HUMAN/1-324 Olfactory receptor 1L3 OS=Homo sapiens GN=OR1L3 PE=2 SV=1

>sp|P34982|OR1D2_HUMAN/1-312 Olfactory receptor 1D2 OS=Homo sapiens GN=OR1D2 PE=1 SV=2

>sp|P47884|OR1D4_HUMAN/1-311 Olfactory receptor 1D4 OS=Homo sapiens GN=OR1D4 PE=2 SV=3

>sp|P58170|OR1D5_HUMAN/1-312 Olfactory receptor 1D5 OS=Homo sapiens GN=OR1D5 PE=2 SV=1

>sp|Q8NGR3|OR1K1_HUMAN/1-316 Olfactory receptor 1K1 OS=Homo sapiens GN=OR1K1 PE=1 SV=1

>sp|O60431|OR1I1_HUMAN/1-355 Olfactory receptor 1I1 OS=Homo sapiens GN=OR1I1 PE=2 SV=1

>sp|Q8NGA1|OR1M1_HUMAN/1-313 Olfactory receptor 1M1 OS=Homo sapiens GN=OR1M1 PE=2 SV=1

>sp|P47890|OR1G1_HUMAN/1-313 Olfactory receptor 1G1 OS=Homo sapiens GN=OR1G1 PE=2 SV=2

>sp|Q6IFN5|O7E24_HUMAN/1-339 Olfactory receptor 7E24 OS=Homo sapiens GN=OR7E24 PE=2 SV=1

>sp|Q8NG98|OR7D4_HUMAN/1-312 Olfactory receptor 7D4 OS=Homo sapiens GN=OR7D4 PE=2 SV=1

>sp|Q96RA2|OR7D2_HUMAN/1-312 Olfactory receptor 7D2 OS=Homo sapiens GN=OR7D2 PE=2 SV=2

>sp|Q15622|OR7A5_HUMAN/1-319 Olfactory receptor 7A5 OS=Homo sapiens GN=OR7A5 PE=2 SV=2

>sp|Q8NGA2|OR7A2_HUMAN/1-310 Putative olfactory receptor 7A2 OS=Homo sapiens GN=OR7A2P PE=5 SV=1

>sp|O76100|OR7AA_HUMAN/1-309 Olfactory receptor 7A10 OS=Homo sapiens GN=OR7A10 PE=2 SV=1

>sp|O14581|OR7AH_HUMAN/1-309 Olfactory receptor 7A17 OS=Homo sapiens GN=OR7A17 PE=2 SV=1

>sp|O76099|OR7C1_HUMAN/1-320 Olfactory receptor 7C1 OS=Homo sapiens GN=OR7C1 PE=2 SV=1

>sp|O60412|OR7C2_HUMAN/1-319 Olfactory receptor 7C2 OS=Homo sapiens GN=OR7C2 PE=2 SV=4

>sp|Q8NG95|OR7G3_HUMAN/1-312 Olfactory receptor 7G3 OS=Homo sapiens GN=OR7G3 PE=2 SV=1

>sp|Q8NGA0|OR7G1_HUMAN/1-311 Olfactory receptor 7G1 OS=Homo sapiens GN=OR7G1 PE=2 SV=2

>sp|Q8NG99|OR7G2_HUMAN/1-324 Olfactory receptor 7G2 OS=Homo sapiens GN=OR7G2 PE=2 SV=1

>sp|Q8NH92|OR1S1_HUMAN/1-325 Olfactory receptor 1S1 OS=Homo sapiens GN=OR1S1 PE=2 SV=2

>sp|Q8NGQ3|OR1S2_HUMAN/1-325 Olfactory receptor 1S2 OS=Homo sapiens GN=OR1S2 PE=2 SV=2

>sp|Q15619|OR1C1_HUMAN/1-314 Olfactory receptor 1C1 OS=Homo sapiens GN=OR1C1 PE=1 SV=4

>sp|Q8WZA6|OR1E3_HUMAN/1-343 Olfactory receptor 1E3 OS=Homo sapiens GN=OR1E3 PE=3 SV=2

>sp|P30953|OR1E1_HUMAN/1-314 Olfactory receptor 1E1 OS=Homo sapiens GN=OR1E1 PE=2 SV=1

>sp|P47887|OR1E2_HUMAN/1-323 Olfactory receptor 1E2 OS=Homo sapiens GN=OR1E2 PE=2 SV=2

>sp|Q8NGS3|OR1J1_HUMAN/1-322 Olfactory receptor 1J1 OS=Homo sapiens GN=OR1J1 PE=2 SV=1

>sp|Q8NGS1|OR1J4_HUMAN/1-313 Olfactory receptor 1J4 OS=Homo sapiens GN=OR1J4 PE=2 SV=1

>sp|Q8NGS2|OR1J2_HUMAN/1-313 Olfactory receptor 1J2 OS=Homo sapiens GN=OR1J2 PE=2 SV=1

>sp|Q8NHA8|OR1FC_HUMAN/1-337 Olfactory receptor 1F12 OS=Homo sapiens GN=OR1F12 PE=3 SV=1

>sp|O43749|OR1F1_HUMAN/1-312 Olfactory receptor 1F1 OS=Homo sapiens GN=OR1F1 PE=2 SV=1

>sp|Q96R84|OR1F2_HUMAN/1-312 Putative olfactory receptor 1F2 OS=Homo sapiens GN=OR1F2P PE=5 SV=2

>sp|Q8NH06|OR1P1_HUMAN/1-330 Olfactory receptor 1P1 OS=Homo sapiens GN=OR1P1 PE=2 SV=2

>sp|Q8NGS0|OR1N1_HUMAN/1-311 Olfactory receptor 1N1 OS=Homo sapiens GN=OR1N1 PE=2 SV=1

>sp|Q8NGR9|OR1N2_HUMAN/1-330 Olfactory receptor 1N2 OS=Homo sapiens GN=OR1N2 PE=2 SV=2

>sp|P47883|OR3A4_HUMAN/1-348 Putative olfactory receptor 3A4 OS=Homo sapiens GN=OR3A4P PE=5 SV=4

>sp|P47881|OR3A1_HUMAN/1-315 Olfactory receptor 3A1 OS=Homo sapiens GN=OR3A1 PE=2 SV=2

>sp|P47893|OR3A2_HUMAN/1-321 Olfactory receptor 3A2 OS=Homo sapiens GN=OR3A2 PE=2 SV=3

>sp|P47888|OR3A3_HUMAN/1-321 Olfactory receptor 3A3 OS=Homo sapiens GN=OR3A3 PE=2 SV=3

>sp|Q8NGE7|OR9K2_HUMAN/1-335 Olfactory receptor 9K2 OS=Homo sapiens GN=OR9K2 PE=2 SV=2

>sp|A6NET4|OR5K3_HUMAN/1-321 Olfactory receptor 5K3 OS=Homo sapiens GN=OR5K3 PE=2 SV=1

>sp|A6NMS3|OR5K4_HUMAN/1-321 Olfactory receptor 5K4 OS=Homo sapiens GN=OR5K4 PE=2 SV=1

>sp|Q8NHB7|OR5K1_HUMAN/1-308 Olfactory receptor 5K1 OS=Homo sapiens GN=OR5K1 PE=2 SV=2

>sp|Q8NHB8|OR5K2_HUMAN/1-316 Olfactory receptor 5K2 OS=Homo sapiens GN=OR5K2 PE=2 SV=3

>sp|Q8NGV7|OR5H2_HUMAN/1-314 Olfactory receptor 5H2 OS=Homo sapiens GN=OR5H2 PE=2 SV=3

>sp|Q8NGV6|OR5H6_HUMAN/1-325 Olfactory receptor 5H6 OS=Homo sapiens GN=OR5H6 PE=2 SV=2

>sp|A6NHG9|O5H14_HUMAN/1-310 Olfactory receptor 5H14 OS=Homo sapiens GN=OR5H14 PE=2 SV=1

>sp|A6NDH6|O5H15_HUMAN/1-313 Olfactory receptor 5H15 OS=Homo sapiens GN=OR5H15 PE=2 SV=1

>sp|A6NKK0|OR5H1_HUMAN/1-313 Olfactory receptor 5H1 OS=Homo sapiens GN=OR5H1 PE=2 SV=1

>sp|P0C628|O5AC1_HUMAN/1-307 Olfactory receptor 5AC1 OS=Homo sapiens GN=OR5AC1 PE=3 SV=1

>sp|Q9NZP5|O5AC2_HUMAN/1-309 Olfactory receptor 5AC2 OS=Homo sapiens GN=OR5AC2 PE=2 SV=2

>sp|Q8WZ92|OR5P2_HUMAN/1-322 Olfactory receptor 5P2 OS=Homo sapiens GN=OR5P2 PE=2 SV=1

>sp|Q8WZ94|OR5P3_HUMAN/1-311 Olfactory receptor 5P3 OS=Homo sapiens GN=OR5P3 PE=2 SV=1

>sp|Q8NH90|O5AK2_HUMAN/1-309 Olfactory receptor 5AK2 OS=Homo sapiens GN=OR5AK2 PE=2 SV=1

>sp|Q8NH89|O5AK3_HUMAN/1-298 Putative olfactory receptor 5AK3 OS=Homo sapiens GN=OR5AK3P PE=5 SV=1

>sp|Q8NGQ1|OR9G4_HUMAN/1-327 Olfactory receptor 9G4 OS=Homo sapiens GN=OR9G4 PE=2 SV=2

>sp|Q8NH87|OR9G1_HUMAN/1-305 Olfactory receptor 9G1 OS=Homo sapiens GN=OR9G1 PE=2 SV=1

>sp|P0C7N8|OR9G9_HUMAN/1-305 Olfactory receptor 9G9 OS=Homo sapiens GN=OR9G9 PE=3 SV=1

>sp|P0C626|OR5G3_HUMAN/1-314 Olfactory receptor 5G3 OS=Homo sapiens GN=OR5G3 PE=3 SV=1

>sp|Q8NGI8|O5AN1_HUMAN/1-311 Olfactory receptor 5AN1 OS=Homo sapiens GN=OR5AN1 PE=2 SV=1

>sp|Q8NGJ0|OR5A1_HUMAN/1-315 Olfactory receptor 5A1 OS=Homo sapiens GN=OR5A1 PE=2 SV=1

>sp|Q8NGI9|OR5A2_HUMAN/1-324 Olfactory receptor 5A2 OS=Homo sapiens GN=OR5A2 PE=2 SV=1

>sp|O95221|OR5F1_HUMAN/1-314 Olfactory receptor 5F1 OS=Homo sapiens GN=OR5F1 PE=2 SV=2

>sp|Q8NGQ6|OR9I1_HUMAN/1-314 Olfactory receptor 9I1 OS=Homo sapiens GN=OR9I1 PE=2 SV=1

>sp|Q8NGQ5|OR9Q1_HUMAN/1-310 Olfactory receptor 9Q1 OS=Homo sapiens GN=OR9Q1 PE=2 SV=1

>sp|Q8NGE9|OR9Q2_HUMAN/1-314 Olfactory receptor 9Q2 OS=Homo sapiens GN=OR9Q2 PE=2 SV=1

>sp|Q8NGC0|O5AU1_HUMAN/1-362 Olfactory receptor 5AU1 OS=Homo sapiens GN=OR5AU1 PE=2 SV=2

>sp|Q8N0Y5|OR8I2_HUMAN/1-310 Olfactory receptor 8I2 OS=Homo sapiens GN=OR8I2 PE=2 SV=1

>sp|Q8NGG4|OR8H1_HUMAN/1-311 Olfactory receptor 8H1 OS=Homo sapiens GN=OR8H1 PE=2 SV=1

>sp|Q8N162|OR8H2_HUMAN/1-312 Olfactory receptor 8H2 OS=Homo sapiens GN=OR8H2 PE=2 SV=1

>sp|Q8N146|OR8H3_HUMAN/1-312 Olfactory receptor 8H3 OS=Homo sapiens GN=OR8H3 PE=2 SV=1

>sp|Q8NH18|OR5J2_HUMAN/1-312 Olfactory receptor 5J2 OS=Homo sapiens GN=OR5J2 PE=2 SV=1

>sp|Q8N127|O5AS1_HUMAN/1-324 Olfactory receptor 5AS1 OS=Homo sapiens GN=OR5AS1 PE=2 SV=1

>sp|Q13606|OR5I1_HUMAN/1-314 Olfactory receptor 5I1 OS=Homo sapiens GN=OR5I1 PE=2 SV=1

>sp|Q8NGL2|OR5L1_HUMAN/1-311 Olfactory receptor 5L1 OS=Homo sapiens GN=OR5L1 PE=2 SV=1

>sp|Q8NGL0|OR5L2_HUMAN/1-311 Olfactory receptor 5L2 OS=Homo sapiens GN=OR5L2 PE=2 SV=1

>sp|Q8NGL4|OR5DD_HUMAN/1-314 Olfactory receptor 5D13 OS=Homo sapiens GN=OR5D13 PE=2 SV=2

>sp|Q8NGL3|OR5DE_HUMAN/1-314 Olfactory receptor 5D14 OS=Homo sapiens GN=OR5D14 PE=2 SV=1

>sp|Q8NGK9|OR5DG_HUMAN/1-328 Olfactory receptor 5D16 OS=Homo sapiens GN=OR5D16 PE=2 SV=1

>sp|Q8NGL1|OR5DI_HUMAN/1-313 Olfactory receptor 5D18 OS=Homo sapiens GN=OR5D18 PE=2 SV=1

>sp|Q15614|OR8G2_HUMAN/1-310 Olfactory receptor 8G2 OS=Homo sapiens GN=OR8G2 PE=2 SV=2

>sp|Q15617|OR8G1_HUMAN/1-311 Olfactory receptor 8G1 OS=Homo sapiens GN=OR8G1 PE=2 SV=2

>sp|Q8NG78|OR8G5_HUMAN/1-311 Olfactory receptor 8G5 OS=Homo sapiens GN=OR8G5 PE=3 SV=1

>sp|Q96RC9|OR8B4_HUMAN/1-309 Olfactory receptor 8B4 OS=Homo sapiens GN=OR8B4 PE=2 SV=2

>sp|Q96RD0|OR8B2_HUMAN/1-313 Olfactory receptor 8B2 OS=Homo sapiens GN=OR8B2 PE=2 SV=3

>sp|Q8NGG8|OR8B3_HUMAN/1-313 Olfactory receptor 8B3 OS=Homo sapiens GN=OR8B3 PE=2 SV=3

>sp|Q15620|OR8B8_HUMAN/1-311 Olfactory receptor 8B8 OS=Homo sapiens GN=OR8B8 PE=2 SV=2

>sp|Q8NGG6|OR8BC_HUMAN/1-310 Olfactory receptor 8B12 OS=Homo sapiens GN=OR8B12 PE=2 SV=1

>sp|Q8NGG7|OR8A1_HUMAN/1-326 Olfactory receptor 8A1 OS=Homo sapiens GN=OR8A1 PE=2 SV=2

>sp|Q8NGM9|OR8D4_HUMAN/1-314 Olfactory receptor 8D4 OS=Homo sapiens GN=OR8D4 PE=2 SV=1

>sp|Q9GZM6|OR8D2_HUMAN/1-311 Olfactory receptor 8D2 OS=Homo sapiens GN=OR8D2 PE=2 SV=1

>sp|Q8WZ84|OR8D1_HUMAN/1-308 Olfactory receptor 8D1 OS=Homo sapiens GN=OR8D1 PE=2 SV=1

>sp|Q8NGP6|OR5M8_HUMAN/1-311 Olfactory receptor 5M8 OS=Homo sapiens GN=OR5M8 PE=2 SV=1

>sp|Q8NGP4|OR5M3_HUMAN/1-307 Olfactory receptor 5M3 OS=Homo sapiens GN=OR5M3 PE=2 SV=2

>sp|Q8NGP3|OR5M9_HUMAN/1-310 Olfactory receptor 5M9 OS=Homo sapiens GN=OR5M9 PE=2 SV=1

>sp|Q96RB7|OR5MB_HUMAN/1-305 Olfactory receptor 5M11 OS=Homo sapiens GN=OR5M11 PE=2 SV=2

>sp|Q8NGP8|OR5M1_HUMAN/1-315 Olfactory receptor 5M1 OS=Homo sapiens GN=OR5M1 PE=2 SV=1

>sp|Q6IEU7|OR5MA_HUMAN/1-315 Olfactory receptor 5M10 OS=Homo sapiens GN=OR5M10 PE=2 SV=1

>sp|P0C617|O5AL1_HUMAN/1-328 Olfactory receptor 5AL1 OS=Homo sapiens GN=OR5AL1 PE=3 SV=1

>sp|Q8NH85|OR5R1_HUMAN/1-324 Olfactory receptor 5R1 OS=Homo sapiens GN=OR5R1 PE=2 SV=1

>sp|P0C7N5|OR8U9_HUMAN/1-309 Olfactory receptor 8U9 OS=Homo sapiens GN=OR8U9 PE=3 SV=1

>sp|Q8NH10|OR8U1_HUMAN/1-309 Olfactory receptor 8U1 OS=Homo sapiens GN=OR8U1 PE=2 SV=1

>sp|P0C7N1|OR8U8_HUMAN/1-319 Olfactory receptor 8U8 OS=Homo sapiens GN=OR8U8 PE=2 SV=1

>sp|Q8NGG1|OR8J2_HUMAN/1-315 Olfactory receptor 8J2 OS=Homo sapiens GN=OR8J2 PE=3 SV=2

>sp|Q8NGP2|OR8J1_HUMAN/1-316 Olfactory receptor 8J1 OS=Homo sapiens GN=OR8J1 PE=2 SV=2

>sp|Q8NGG0|OR8J3_HUMAN/1-315 Olfactory receptor 8J3 OS=Homo sapiens GN=OR8J3 PE=2 SV=1

>sp|Q8NGG5|OR8K1_HUMAN/1-319 Olfactory receptor 8K1 OS=Homo sapiens GN=OR8K1 PE=2 SV=1

>sp|Q8NH50|OR8K5_HUMAN/1-307 Olfactory receptor 8K5 OS=Homo sapiens GN=OR8K5 PE=2 SV=1

>sp|Q8NH51|OR8K3_HUMAN/1-312 Olfactory receptor 8K3 OS=Homo sapiens GN=OR8K3 PE=2 SV=1

>sp|Q8NGG2|OR5T2_HUMAN/1-359 Olfactory receptor 5T2 OS=Homo sapiens GN=OR5T2 PE=2 SV=3

>sp|Q8NG75|OR5T1_HUMAN/1-326 Olfactory receptor 5T1 OS=Homo sapiens GN=OR5T1 PE=2 SV=1

>sp|Q8NGG3|OR5T3_HUMAN/1-340 Olfactory receptor 5T3 OS=Homo sapiens GN=OR5T3 PE=2 SV=3

>sp|Q8NGF4|O5AP2_HUMAN/1-316 Olfactory receptor 5AP2 OS=Homo sapiens GN=OR5AP2 PE=2 SV=1

>sp|Q8NGP9|O5AR1_HUMAN/1-310 Olfactory receptor 5AR1 OS=Homo sapiens GN=OR5AR1 PE=1 SV=1

>sp|A6NL26|OR5BL_HUMAN/1-309 Olfactory receptor 5B21 OS=Homo sapiens GN=OR5B21 PE=2 SV=1

>sp|Q8NGF7|OR5BH_HUMAN/1-314 Olfactory receptor 5B17 OS=Homo sapiens GN=OR5B17 PE=2 SV=1

>sp|Q96R08|OR5BC_HUMAN/1-314 Olfactory receptor 5B12 OS=Homo sapiens GN=OR5B12 PE=2 SV=2

>sp|Q96R09|OR5B2_HUMAN/1-309 Olfactory receptor 5B2 OS=Homo sapiens GN=OR5B2 PE=2 SV=3

>sp|Q8NH48|OR5B3_HUMAN/1-314 Olfactory receptor 5B3 OS=Homo sapiens GN=OR5B3 PE=2 SV=1

>sp|Q8NH69|OR5W2_HUMAN/1-310 Olfactory receptor 5W2 OS=Homo sapiens GN=OR5W2 PE=2 SV=1

>sp|Q8NGR4|OR5C1_HUMAN/1-320 Olfactory receptor 5C1 OS=Homo sapiens GN=OR5C1 PE=2 SV=1

>sp|Q8NG92|O13H1_HUMAN/1-308 Olfactory receptor 13H1 OS=Homo sapiens GN=OR13H1 PE=2 SV=1

>sp|Q8NGF6|O10W1_HUMAN/1-305 Olfactory receptor 10W1 OS=Homo sapiens GN=OR10W1 PE=2 SV=1

>sp|Q8NGQ4|O10Q1_HUMAN/1-319 Olfactory receptor 10Q1 OS=Homo sapiens GN=OR10Q1 PE=2 SV=1

>sp|Q8NGI7|O10V1_HUMAN/1-309 Olfactory receptor 10V1 OS=Homo sapiens GN=OR10V1 PE=2 SV=3

>sp|Q8NH19|O10AG_HUMAN/1-301 Olfactory receptor 10AG1 OS=Homo sapiens GN=OR10AG1 PE=2 SV=1

>sp|A4D2G3|O2A25_HUMAN/1-310 Olfactory receptor 2A25 OS=Homo sapiens GN=OR2A25 PE=2 SV=2

>sp|O95047|OR2A4_HUMAN/1-310 Olfactory receptor 2A4 OS=Homo sapiens GN=OR2A4 PE=2 SV=1

>sp|Q96R45|OR2A7_HUMAN/1-310 Olfactory receptor 2A7 OS=Homo sapiens GN=OR2A7 PE=2 SV=3

>sp|Q8NGT7|O2A12_HUMAN/1-310 Olfactory receptor 2A12 OS=Homo sapiens GN=OR2A12 PE=3 SV=1

>sp|Q6IF42|OR2A2_HUMAN/1-318 Olfactory receptor 2A2 OS=Homo sapiens GN=OR2A2 PE=2 SV=2

>sp|Q96R47|O2A14_HUMAN/1-310 Olfactory receptor 2A14 OS=Homo sapiens GN=OR2A14 PE=2 SV=4

>sp|Q8NGT9|OR2A1_HUMAN/1-310 Olfactory receptor 2A1/2A42 OS=Homo sapiens GN=OR2A1 PE=2 SV=2

>sp|Q96R48|OR2A5_HUMAN/1-311 Olfactory receptor 2A5 OS=Homo sapiens GN=OR2A5 PE=2 SV=2

>sp|Q8NGU4|OR2I1_HUMAN/1-316 Putative olfactory receptor 2I1 OS=Homo sapiens GN=OR2I1P PE=5 SV=1

>sp|Q5JQS5|OR2BB_HUMAN/1-317 Olfactory receptor 2B11 OS=Homo sapiens GN=OR2B11 PE=2 SV=1

>sp|Q8N628|OR2C3_HUMAN/1-320 Olfactory receptor 2C3 OS=Homo sapiens GN=OR2C3 PE=2 SV=3

>sp|O95371|OR2C1_HUMAN/1-312 Olfactory receptor 2C1 OS=Homo sapiens GN=OR2C1 PE=2 SV=3

>sp|Q9GZK4|OR2H1_HUMAN/1-316 Olfactory receptor 2H1 OS=Homo sapiens GN=OR2H1 PE=2 SV=1

>sp|O95918|OR2H2_HUMAN/1-312 Olfactory receptor 2H2 OS=Homo sapiens GN=OR2H2 PE=2 SV=2

>sp|O76000|OR2B3_HUMAN/1-313 Putative olfactory receptor 2B3 OS=Homo sapiens GN=OR2B3 PE=2 SV=1

>sp|Q9GZK3|OR2B2_HUMAN/1-357 Olfactory receptor 2B2 OS=Homo sapiens GN=OR2B2 PE=2 SV=1

>sp|P58173|OR2B6_HUMAN/1-313 Olfactory receptor 2B6 OS=Homo sapiens GN=OR2B6 PE=2 SV=1

>sp|O76001|OR2J3_HUMAN/1-311 Olfactory receptor 2J3 OS=Homo sapiens GN=OR2J3 PE=2 SV=1

>sp|Q9GZK6|OR2J1_HUMAN/1-312 Olfactory receptor 2J1 OS=Homo sapiens GN=OR2J1 PE=3 SV=2

>sp|O76002|OR2J2_HUMAN/1-312 Olfactory receptor 2J2 OS=Homo sapiens GN=OR2J2 PE=2 SV=1

>sp|Q9Y3N9|OR2W1_HUMAN/1-320 Olfactory receptor 2W1 OS=Homo sapiens GN=OR2W1 PE=2 SV=1

>sp|Q7Z3T1|OR2W3_HUMAN/1-314 Olfactory receptor 2W3 OS=Homo sapiens GN=OR2W3 PE=2 SV=2

>sp|Q8NHA6|OR2W6_HUMAN/1-318 Putative olfactory receptor 2W6 OS=Homo sapiens GN=OR2W6P PE=5 SV=1

>sp|Q8NGZ5|OR2G2_HUMAN/1-317 Olfactory receptor 2G2 OS=Homo sapiens GN=OR2G2 PE=2 SV=1

>sp|Q5TZ20|OR2G6_HUMAN/1-316 Olfactory receptor 2G6 OS=Homo sapiens GN=OR2G6 PE=2 SV=1

>sp|Q8NGZ4|OR2G3_HUMAN/1-309 Olfactory receptor 2G3 OS=Homo sapiens GN=OR2G3 PE=2 SV=1

>sp|Q8NGV0|OR2Y1_HUMAN/1-311 Olfactory receptor 2Y1 OS=Homo sapiens GN=OR2Y1 PE=2 SV=1

>sp|P59922|OR2B8_HUMAN/1-312 Putative olfactory receptor 2B8 OS=Homo sapiens GN=OR2B8P PE=5 SV=1

>sp|Q8NGY0|O10X1_HUMAN/1-326 Olfactory receptor 10X1 OS=Homo sapiens GN=OR10X1 PE=2 SV=2

>sp|Q8NGT2|O13J1_HUMAN/1-312 Olfactory receptor 13J1 OS=Homo sapiens GN=OR13J1 PE=2 SV=1

>sp|Q8NGS4|O13F1_HUMAN/1-319 Olfactory receptor 13F1 OS=Homo sapiens GN=OR13F1 PE=2 SV=1

>sp|Q8NGT1|OR2K2_HUMAN/1-345 Olfactory receptor 2K2 OS=Homo sapiens GN=OR2K2 PE=2 SV=2

>sp|Q8NGV5|O13D1_HUMAN/1-346 Olfactory receptor 13D1 OS=Homo sapiens GN=OR13D1 PE=2 SV=3

>sp|Q8NGT0|O13C9_HUMAN/1-318 Olfactory receptor 13C9 OS=Homo sapiens GN=OR13C9 PE=2 SV=1

>sp|Q8NGS9|O13C2_HUMAN/1-318 Olfactory receptor 13C2 OS=Homo sapiens GN=OR13C2 PE=2 SV=1

>sp|Q8NGS8|O13C5_HUMAN/1-318 Olfactory receptor 13C5 OS=Homo sapiens GN=OR13C5 PE=2 SV=1

>sp|Q8NGS6|O13C3_HUMAN/1-347 Olfactory receptor 13C3 OS=Homo sapiens GN=OR13C3 PE=2 SV=2

>sp|Q8NGS5|O13C4_HUMAN/1-318 Olfactory receptor 13C4 OS=Homo sapiens GN=OR13C4 PE=2 SV=1

>sp|Q8NGS7|O13C8_HUMAN/1-320 Olfactory receptor 13C8 OS=Homo sapiens GN=OR13C8 PE=2 SV=1

>sp|Q9NQN1|OR2S1_HUMAN/1-319 Olfactory receptor 2S2 OS=Homo sapiens GN=OR2S2 PE=2 SV=2

>sp|Q8NH95|YI035_HUMAN/1-320 Putative olfactory receptor ENSP00000348552 OS=Homo sapiens PE=5 SV=1

>sp|Q13607|OR2F1_HUMAN/1-317 Olfactory receptor 2F1 OS=Homo sapiens GN=OR2F1 PE=2 SV=2

>sp|O95006|OR2F2_HUMAN/1-317 Olfactory receptor 2F2 OS=Homo sapiens GN=OR2F2 PE=2 SV=1

>sp|Q9H210|OR2D2_HUMAN/1-308 Olfactory receptor 2D2 OS=Homo sapiens GN=OR2D2 PE=2 SV=4

>sp|Q8NGH3|OR2D3_HUMAN/1-330 Olfactory receptor 2D3 OS=Homo sapiens GN=OR2D3 PE=2 SV=2

>sp|Q8NGE3|O10P1_HUMAN/1-313 Olfactory receptor 10P1 OS=Homo sapiens GN=OR10P1 PE=2 SV=1

>sp|Q96KK4|O10C1_HUMAN/1-312 Olfactory receptor 10C1 OS=Homo sapiens GN=OR10C1 PE=2 SV=3

>sp|P58181|O10A3_HUMAN/1-314 Olfactory receptor 10A3 OS=Homo sapiens GN=OR10A3 PE=2 SV=1

>sp|Q8NH74|O10A6_HUMAN/1-314 Olfactory receptor 10A6 OS=Homo sapiens GN=OR10A6 PE=2 SV=1

>sp|Q8NGE5|O10A7_HUMAN/1-316 Olfactory receptor 10A7 OS=Homo sapiens GN=OR10A7 PE=2 SV=1

>sp|Q9H209|O10A4_HUMAN/1-315 Olfactory receptor 10A4 OS=Homo sapiens GN=OR10A4 PE=2 SV=2

>sp|Q9H208|O10A2_HUMAN/1-303 Olfactory receptor 10A2 OS=Homo sapiens GN=OR10A2 PE=2 SV=2

>sp|Q9H207|O10A5_HUMAN/1-317 Olfactory receptor 10A5 OS=Homo sapiens GN=OR10A5 PE=2 SV=1

>sp|O60404|O10H3_HUMAN/1-316 Olfactory receptor 10H3 OS=Homo sapiens GN=OR10H3 PE=2 SV=1

>sp|Q8NGA5|O10H4_HUMAN/1-316 Olfactory receptor 10H4 OS=Homo sapiens GN=OR10H4 PE=2 SV=1

>sp|O60403|O10H2_HUMAN/1-315 Olfactory receptor 10H2 OS=Homo sapiens GN=OR10H2 PE=2 SV=1

>sp|Q9Y4A9|O10H1_HUMAN/1-318 Olfactory receptor 10H1 OS=Homo sapiens GN=OR10H1 PE=2 SV=1

>sp|Q8NGA6|O10H5_HUMAN/1-315 Olfactory receptor 10H5 OS=Homo sapiens GN=OR10H5 PE=2 SV=1

>sp|Q9UGF6|OR5V1_HUMAN/1-321 Olfactory receptor 5V1 OS=Homo sapiens GN=OR5V1 PE=2 SV=1

>sp|Q8NGX5|O10K1_HUMAN/1-313 Olfactory receptor 10K1 OS=Homo sapiens GN=OR10K1 PE=2 SV=1

>sp|Q6IF99|O10K2_HUMAN/1-312 Olfactory receptor 10K2 OS=Homo sapiens GN=OR10K2 PE=2 SV=1

>sp|Q8NGX6|O10R2_HUMAN/1-335 Olfactory receptor 10R2 OS=Homo sapiens GN=OR10R2 PE=2 SV=3

>sp|Q8NGX3|O10T2_HUMAN/1-314 Olfactory receptor 10T2 OS=Homo sapiens GN=OR10T2 PE=2 SV=1

>sp|Q8NGY1|O10Z1_HUMAN/1-313 Olfactory receptor 10Z1 OS=Homo sapiens GN=OR10Z1 PE=2 SV=1

>sp|Q5JRS4|O10J3_HUMAN/1-329 Olfactory receptor 10J3 OS=Homo sapiens GN=OR10J3 PE=2 SV=1

>sp|P0C629|O10J4_HUMAN/1-311 Olfactory receptor 10J4 OS=Homo sapiens GN=OR10J4 PE=3 SV=1

>sp|P30954|O10J1_HUMAN/1-320 Olfactory receptor 10J1 OS=Homo sapiens GN=OR10J1 PE=2 SV=2

>sp|Q8NGY7|O10J6_HUMAN/1-276 Putative olfactory receptor 10J6 OS=Homo sapiens GN=OR10J6P PE=5 SV=1

>sp|Q8NHC4|O10J5_HUMAN/1-309 Olfactory receptor 10J5 OS=Homo sapiens GN=OR10J5 PE=2 SV=1

>sp|Q8NH54|O56A3_HUMAN/1-315 Olfactory receptor 56A3 OS=Homo sapiens GN=OR56A3 PE=1 SV=2

>sp|Q8NGH5|O56A1_HUMAN/1-318 Olfactory receptor 56A1 OS=Homo sapiens GN=OR56A1 PE=2 SV=3

>sp|Q8NGH8|O56A4_HUMAN/1-313 Olfactory receptor 56A4 OS=Homo sapiens GN=OR56A4 PE=2 SV=2

>sp|P0C7T3|O56A5_HUMAN/1-313 Olfactory receptor 56A5 OS=Homo sapiens GN=OR56A5 PE=3 SV=1

>sp|Q8NH76|O56B4_HUMAN/1-319 Olfactory receptor 56B4 OS=Homo sapiens GN=OR56B4 PE=2 SV=1

>sp|Q8NGI3|O56B1_HUMAN/1-324 Olfactory receptor 56B1 OS=Homo sapiens GN=OR56B1 PE=2 SV=2

>sp|Q8NGI1|O56B2_HUMAN/1-322 Putative olfactory receptor 56B2 OS=Homo sapiens GN=OR56B2P PE=5 SV=1

>sp|Q9H342|O51J1_HUMAN/1-316 Olfactory receptor 51J1 OS=Homo sapiens GN=OR51J1 PE=2 SV=2

>sp|Q8NGJ8|O51S1_HUMAN/1-323 Olfactory receptor 51S1 OS=Homo sapiens GN=OR51S1 PE=2 SV=1

>sp|Q8NGK6|O52I1_HUMAN/1-324 Olfactory receptor 52I1 OS=Homo sapiens GN=OR52I1 PE=2 SV=2

>sp|Q8NH67|O52I2_HUMAN/1-350 Olfactory receptor 52I2 OS=Homo sapiens GN=OR52I2 PE=2 SV=3

>sp|Q6IF63|O52W1_HUMAN/1-320 Olfactory receptor 52W1 OS=Homo sapiens GN=OR52W1 PE=2 SV=2

>sp|Q8NGJ9|O51T1_HUMAN/1-327 Olfactory receptor 51T1 OS=Homo sapiens GN=OR51T1 PE=2 SV=1

>sp|Q9H2C8|O51V1_HUMAN/1-321 Olfactory receptor 51V1 OS=Homo sapiens GN=OR51V1 PE=2 SV=2

>sp|Q9H341|O51M1_HUMAN/1-315 Olfactory receptor 51M1 OS=Homo sapiens GN=OR51M1 PE=2 SV=3

>sp|Q8TCB6|O51E1_HUMAN/1-317 Olfactory receptor 51E1 OS=Homo sapiens GN=OR51E1 PE=2 SV=1

>sp|Q8NGF3|O51D1_HUMAN/1-324 Olfactory receptor 51D1 OS=Homo sapiens GN=OR51D1 PE=2 SV=1

>sp|Q9H255|O51E2_HUMAN/1-320 Olfactory receptor 51E2 OS=Homo sapiens GN=OR51E2 PE=2 SV=1

>sp|A6NGY5|O51F1_HUMAN/1-319 Olfactory receptor 51F1 OS=Homo sapiens GN=OR51F1 PE=2 SV=1

>sp|Q8NH61|O51F2_HUMAN/1-342 Olfactory receptor 51F2 OS=Homo sapiens GN=OR51F2 PE=2 SV=2

>sp|Q8NH59|O51Q1_HUMAN/1-317 Olfactory receptor 51Q1 OS=Homo sapiens GN=OR51Q1 PE=2 SV=2

>sp|Q9H344|O51I2_HUMAN/1-312 Olfactory receptor 51I2 OS=Homo sapiens GN=OR51I2 PE=2 SV=1

>sp|Q9H343|O51I1_HUMAN/1-314 Olfactory receptor 51I1 OS=Homo sapiens GN=OR51I1 PE=2 SV=1

>sp|Q9Y5P0|O51B4_HUMAN/1-310 Olfactory receptor 51B4 OS=Homo sapiens GN=OR51B4 PE=2 SV=3

>sp|Q9Y5P1|O51B2_HUMAN/1-312 Olfactory receptor 51B2 OS=Homo sapiens GN=OR51B2 PE=2 SV=4

>sp|Q9H339|O51B5_HUMAN/1-312 Olfactory receptor 51B5 OS=Homo sapiens GN=OR51B5 PE=2 SV=2

>sp|Q9H340|O51B6_HUMAN/1-312 Olfactory receptor 51B6 OS=Homo sapiens GN=OR51B6 PE=2 SV=2

>sp|Q8NH63|O51H1_HUMAN/1-302 Putative olfactory receptor 51H1 OS=Homo sapiens GN=OR51H1P PE=5 SV=1

>sp|Q8NGJ5|O51L1_HUMAN/1-315 Olfactory receptor 51L1 OS=Homo sapiens GN=OR51L1 PE=2 SV=1

>sp|Q8NH64|O51A7_HUMAN/1-312 Olfactory receptor 51A7 OS=Homo sapiens GN=OR51A7 PE=2 SV=1

>sp|Q8NGJ7|O51A2_HUMAN/1-313 Olfactory receptor 51A2 OS=Homo sapiens GN=OR51A2 PE=2 SV=1

>sp|Q8NGJ6|O51A4_HUMAN/1-313 Olfactory receptor 51A4 OS=Homo sapiens GN=OR51A4 PE=2 SV=1

>sp|Q8NGK1|O51G1_HUMAN/1-321 Olfactory receptor 51G1 OS=Homo sapiens GN=OR51G1 PE=2 SV=1

>sp|Q8NGK0|O51G2_HUMAN/1-314 Olfactory receptor 51G2 OS=Homo sapiens GN=OR51G2 PE=2 SV=1

>sp|A6NMU1|O52A4_HUMAN/1-304 Putative olfactory receptor 52A4 OS=Homo sapiens GN=OR52A4 PE=5 SV=1

>sp|Q9UKL2|O52A1_HUMAN/1-312 Olfactory receptor 52A1 OS=Homo sapiens GN=OR52A1 PE=2 SV=2

>sp|Q9H2C5|O52A5_HUMAN/1-316 Olfactory receptor 52A5 OS=Homo sapiens GN=OR52A5 PE=2 SV=1

>sp|Q8NGK5|O52M1_HUMAN/1-317 Olfactory receptor 52M1 OS=Homo sapiens GN=OR52M1 PE=2 SV=1

>sp|Q8NGK4|O52K1_HUMAN/1-314 Olfactory receptor 52K1 OS=Homo sapiens GN=OR52K1 PE=2 SV=2

>sp|Q8NGK3|O52K2_HUMAN/1-314 Olfactory receptor 52K2 OS=Homo sapiens GN=OR52K2 PE=2 SV=2

>sp|Q8NH56|O52N5_HUMAN/1-324 Olfactory receptor 52N5 OS=Homo sapiens GN=OR52N5 PE=2 SV=2

>sp|Q8NGI2|O52N4_HUMAN/1-321 Olfactory receptor 52N4 OS=Homo sapiens GN=OR52N4 PE=2 SV=2

>sp|Q8NGI0|O52N2_HUMAN/1-321 Olfactory receptor 52N2 OS=Homo sapiens GN=OR52N2 PE=2 SV=1

>sp|Q8NH53|O52N1_HUMAN/1-320 Olfactory receptor 52N1 OS=Homo sapiens GN=OR52N1 PE=2 SV=1

>sp|Q8NH57|O52P1_HUMAN/1-321 Putative olfactory receptor 52P1 OS=Homo sapiens GN=OR52P1P PE=5 SV=2

>sp|Q8NGF1|O52R1_HUMAN/1-315 Olfactory receptor 52R1 OS=Homo sapiens GN=OR52R1 PE=2 SV=2

>sp|Q8NGH7|O52L1_HUMAN/1-329 Olfactory receptor 52L1 OS=Homo sapiens GN=OR52L1 PE=2 SV=4

>sp|Q8NGH6|O52L2_HUMAN/1-319 Putative olfactory receptor 52L2 OS=Homo sapiens GN=OR52L2P PE=5 SV=3

>sp|Q8NGK2|O52B4_HUMAN/1-314 Olfactory receptor 52B4 OS=Homo sapiens GN=OR52B4 PE=2 SV=2

>sp|Q96RD2|O52B2_HUMAN/1-323 Olfactory receptor 52B2 OS=Homo sapiens GN=OR52B2 PE=2 SV=3

>sp|Q8NGJ2|O52H1_HUMAN/1-320 Olfactory receptor 52H1 OS=Homo sapiens GN=OR52H1 PE=2 SV=3

>sp|P0C646|O52Z1_HUMAN/1-297 Olfactory receptor 52Z1 OS=Homo sapiens GN=OR52Z1 PE=3 SV=1

>sp|Q8NGF0|O52B6_HUMAN/1-335 Olfactory receptor 52B6 OS=Homo sapiens GN=OR52B6 PE=2 SV=3

>sp|Q9H346|O52D1_HUMAN/1-318 Olfactory receptor 52D1 OS=Homo sapiens GN=OR52D1 PE=2 SV=1

>sp|Q8NH60|O52J3_HUMAN/1-311 Olfactory receptor 52J3 OS=Homo sapiens GN=OR52J3 PE=2 SV=2

>sp|Q8NH55|O52E5_HUMAN/1-315 Olfactory receptor 52E5 OS=Homo sapiens GN=OR52E5 PE=3 SV=1

>sp|Q96RD3|O52E6_HUMAN/1-313 Olfactory receptor 52E6 OS=Homo sapiens GN=OR52E6 PE=2 SV=2

>sp|Q6IFG1|O52E8_HUMAN/1-317 Olfactory receptor 52E8 OS=Homo sapiens GN=OR52E8 PE=1 SV=3

>sp|Q8NGH9|O52E4_HUMAN/1-312 Olfactory receptor 52E4 OS=Homo sapiens GN=OR52E4 PE=2 SV=1

>sp|Q8NGJ3|O52E1_HUMAN/1-308 Olfactory receptor 52E1 OS=Homo sapiens GN=OR52E1 PE=3 SV=1

>sp|Q8NGJ4|O52E2_HUMAN/1-325 Olfactory receptor 52E2 OS=Homo sapiens GN=OR52E2 PE=2 SV=2

Group 8a(a):

>sp|Q6DWJ6|GP139_HUMAN/1-353 Probable G-protein coupled receptor 139 OS=Homo sapiens GN=GPR139 PE=2 SV=1

Group 8a(b):

>sp|P51810|GP143_HUMAN/1-404 G-protein coupled receptor 143 OS=Homo sapiens GN=GPR143 PE=1 SV=2

Group 8a(c):

>sp|Q9NZH0|GPC5B_HUMAN/1-403 G-protein coupled receptor family C group 5 member B OS=Homo sapiens GN=GPRC5B PE=2 SV=2

>sp|Q9NZD1|GPC5D_HUMAN/1-345 G-protein coupled receptor family C group 5 member D OS=Homo sapiens GN=GPRC5D PE=2 SV=1

>sp|Q8NFJ5|RAI3_HUMAN/1-357 Retinoic acid-induced protein 3 OS=Homo sapiens GN=GPRC5A PE=1 SV=2

Group 8b(a):

>sp|Q9UJ42|GP160_HUMAN/1-338 Probable G-protein coupled receptor 160 OS=Homo sapiens GN=GPR160 PE=2 SV=1

Group 8b(b):

>sp|Q99679|GPR21_HUMAN/1-349 Probable G-protein coupled receptor 21 OS=Homo sapiens GN=GPR21 PE=2 SV=1

>sp|Q9Y2T5|GPR52_HUMAN/1-361 Probable G-protein coupled receptor 52 OS=Homo sapiens GN=GPR52 PE=2 SV=2

Group 8c(a):

>sp|Q8TDV5|GP119_HUMAN/1-335 Glucose-dependent insulinotropic receptor OS=Homo sapiens GN=GPR119 PE=1 SV=1

>sp|Q8TDU6|GPBAR_HUMAN/1-330 G-protein coupled bile acid receptor 1 OS=Homo sapiens GN=GPBAR1 PE=2 SV=1

Group 8c(b):

>sp|Q8NDV2|GPR26_HUMAN/1-337 G-protein coupled receptor 26 OS=Homo sapiens GN=GPR26 PE=1 SV=1

>sp|Q96P69|GPR78_HUMAN/1-363 G-protein coupled receptor 78 OS=Homo sapiens GN=GPR78 PE=1 SV=2

>sp|P33765|AA3R_HUMAN/1-318 Adenosine receptor A3 OS=Homo sapiens GN=ADORA3 PE=2 SV=2

>sp|P30542|AA1R_HUMAN/1-326 Adenosine receptor A1 OS=Homo sapiens GN=ADORA1 PE=1 SV=1

>sp|P29274|AA2AR_HUMAN/1-412 Adenosine receptor A2a OS=Homo sapiens GN=ADORA2A PE=1 SV=2

>sp|P29275|AA2BR_HUMAN/1-332 Adenosine receptor A2b OS=Homo sapiens GN=ADORA2B PE=2 SV=1

Group 8d:

>sp|P43119|PI2R_HUMAN/1-386 Prostacyclin receptor OS=Homo sapiens GN=PTGIR PE=1 SV=1

>sp|Q13258|PD2R_HUMAN/1-359 Prostaglandin D2 receptor OS=Homo sapiens GN=PTGDR PE=2 SV=2

>sp|P43116|PE2R2_HUMAN/1-358 Prostaglandin E2 receptor EP2 subtype OS=Homo sapiens GN=PTGER2 PE=2 SV=2

>sp|P43115|PE2R3_HUMAN/1-390 Prostaglandin E2 receptor EP3 subtype OS=Homo sapiens GN=PTGER3 PE=2 SV=1

>sp|P43088|PF2R_HUMAN/1-359 Prostaglandin F2-alpha receptor OS=Homo sapiens GN=PTGFR PE=2 SV=1

>sp|P21731|TA2R_HUMAN/1-343 Thromboxane A2 receptor OS=Homo sapiens GN=TBXA2R PE=1 SV=3

>sp|P34995|PE2R1_HUMAN/1-402 Prostaglandin E2 receptor EP1 subtype OS=Homo sapiens GN=PTGER1 PE=2 SV=3

Group 8e:

>sp|P04001|OPSG_HUMAN/1-364 Medium-wave-sensitive opsin 1 OS=Homo sapiens GN=OPN1MW PE=1 SV=1

>sp|P04000|OPSR_HUMAN/1-364 Long-wave-sensitive opsin 1 OS=Homo sapiens GN=OPN1LW PE=1 SV=1

>sp|P08100|OPSD_HUMAN/1-348 Rhodopsin OS=Homo sapiens GN=RHO PE=1 SV=1

>sp|P03999|OPSB_HUMAN/1-348 Short-wave-sensitive opsin 1 OS=Homo sapiens GN=OPN1SW PE=1 SV=1

>sp|P47804|RGR_HUMAN/1-291 RPE-retinal G protein-coupled receptor OS=Homo sapiens GN=RGR PE=1 SV=1

>sp|Q6U736|OPN5_HUMAN/1-354 Opsin-5 OS=Homo sapiens GN=OPN5 PE=1 SV=3

>sp|O14718|OPSX_HUMAN/1-337 Visual pigment-like receptor peropsin OS=Homo sapiens GN=RRH PE=1 SV=1

Group 8f(a):

>sp|P21554|CNR1_HUMAN/1-472 Cannabinoid receptor 1 OS=Homo sapiens GN=CNR1 PE=1 SV=1

>sp|P34972|CNR2_HUMAN/1-360 Cannabinoid receptor 2 OS=Homo sapiens GN=CNR2 PE=1 SV=1

Group 8f(b):

>sp|Q5UAW9|GP157_HUMAN/1-335 Probable G-protein coupled receptor 157 OS=Homo sapiens GN=GPR157 PE=1 SV=2

Group 8f(c):

>sp|P47775|GPR12_HUMAN/1-334 G-protein coupled receptor 12 OS=Homo sapiens GN=GPR12 PE=1 SV=1

>sp|P46089|GPR3_HUMAN/1-330 G-protein coupled receptor 3 OS=Homo sapiens GN=GPR3 PE=1 SV=1

>sp|P46095|GPR6_HUMAN/1-362 G-protein coupled receptor 6 OS=Homo sapiens GN=GPR6 PE=1 SV=1

>sp|Q9UBY5|LPAR3_HUMAN/1-353 Lysophosphatidic acid receptor 3 OS=Homo sapiens GN=LPAR3 PE=2 SV=1

>sp|Q9HBW0|LPAR2_HUMAN/1-351 Lysophosphatidic acid receptor 2 OS=Homo sapiens GN=LPAR2 PE=1 SV=2

>sp|Q92633|LPAR1_HUMAN/1-364 Lysophosphatidic acid receptor 1 OS=Homo sapiens GN=LPAR1 PE=1 SV=3

>sp|O95977|S1PR4_HUMAN/1-384 Sphingosine 1-phosphate receptor 4 OS=Homo sapiens GN=S1PR4 PE=1 SV=1

>sp|P21453|S1PR1_HUMAN/1-382 Sphingosine 1-phosphate receptor 1 OS=Homo sapiens GN=S1PR1 PE=1 SV=2

>sp|Q99500|S1PR3_HUMAN/1-378 Sphingosine 1-phosphate receptor 3 OS=Homo sapiens GN=S1PR3 PE=1 SV=2

>sp|Q9H228|S1PR5_HUMAN/1-398 Sphingosine 1-phosphate receptor 5 OS=Homo sapiens GN=S1PR5 PE=2 SV=1

>sp|O95136|S1PR2_HUMAN/1-353 Sphingosine 1-phosphate receptor 2 OS=Homo sapiens GN=S1PR2 PE=1 SV=2

Group 8h:

>sp|Q96AM1|MRGRF_HUMAN/1-343 Mas-related G-protein coupled receptor member F OS=Homo sapiens GN=MRGPRF PE=2 SV=1

>sp|P04201|MAS_HUMAN/1-325 Proto-oncogene Mas OS=Homo sapiens GN=MAS1 PE=1 SV=1

>sp|P35410|MAS1L_HUMAN/1-378 Mas-related G-protein coupled receptor MRG OS=Homo sapiens GN=MAS1L PE=2 SV=1

>sp|Q8TDS7|MRGRD_HUMAN/1-321 Mas-related G-protein coupled receptor member D OS=Homo sapiens GN=MRGPRD PE=2 SV=1

>sp|Q96LB1|MRGX2_HUMAN/1-330 Mas-related G-protein coupled receptor member X2 OS=Homo sapiens GN=MRGPRX2 PE=2 SV=1

>sp|Q96LA9|MRGX4_HUMAN/1-322 Mas-related G-protein coupled receptor member X4 OS=Homo sapiens GN=MRGPRX4 PE=2 SV=2

>sp|Q96LB2|MRGX1_HUMAN/1-322 Mas-related G-protein coupled receptor member X1 OS=Homo sapiens GN=MRGPRX1 PE=1 SV=1

>sp|Q96LB0|MRGX3_HUMAN/1-322 Mas-related G-protein coupled receptor member X3 OS=Homo sapiens GN=MRGPRX3 PE=2 SV=2

>sp|Q86SM5|MRGRG_HUMAN/1-289 Mas-related G-protein coupled receptor member G OS=Homo sapiens GN=MRGPRG PE=2 SV=2

>sp|Q86SM8|MRGRE_HUMAN/1-311 Mas-related G-protein coupled receptor member E OS=Homo sapiens GN=MRGPRE PE=2 SV=2

Group 8i:

>sp|P30968|GNRHR_HUMAN/1-328 Gonadotropin-releasing hormone receptor OS=Homo sapiens GN=GNRHR PE=1 SV=1

>sp|Q8TDV2|GP148_HUMAN/1-347 Probable G-protein coupled receptor 148 OS=Homo sapiens GN=GPR148 PE=2 SV=2

Group 8j:

>sp|O95800|GPR75_HUMAN/1-540 Probable G-protein coupled receptor 75 OS=Homo sapiens GN=GPR75 PE=2 SV=1

>sp|P35408|PE2R4_HUMAN/1-488 Prostaglandin E2 receptor EP4 subtype OS=Homo sapiens GN=PTGER4 PE=1 SV=1

>sp|Q6NV75|GP153_HUMAN/1-609 Probable G-protein coupled receptor 153 OS=Homo sapiens GN=GPR153 PE=2 SV=2

>sp|Q16538|GP162_HUMAN/1-588 Probable G-protein coupled receptor 162 OS=Homo sapiens GN=GPR162 PE=2 SV=1

>sp|Q14439|GP176_HUMAN/1-515 Probable G-protein coupled receptor 176 OS=Homo sapiens GN=GPR176 PE=2 SV=1

>sp|Q8TDT2|GP152_HUMAN/1-470 Probable G-protein coupled receptor 152 OS=Homo sapiens GN=GPR152 PE=2 SV=1

>sp|Q8N6U8|GP161_HUMAN/1-529 G-protein coupled receptor 161 OS=Homo sapiens GN=GPR161 PE=2 SV=1

>sp|Q13585|MTR1L_HUMAN/1-617 Melatonin-related receptor OS=Homo sapiens GN=GPR50 PE=1 SV=3

Group 8k:

>sp|Q9BZJ8|GPR61_HUMAN/1-451 Probable G-protein coupled receptor 61 OS=Homo sapiens GN=GPR61 PE=2 SV=2

>sp|Q9BZJ7|GPR62_HUMAN/1-368 Probable G-protein coupled receptor 62 OS=Homo sapiens GN=GPR62 PE=2 SV=2

Group 8l(a):

>sp|Q15760|GPR19_HUMAN/1-415 Probable G-protein coupled receptor 19 OS=Homo sapiens GN=GPR19 PE=2 SV=2

>sp|Q9NYM4|GPR83_HUMAN/1-423 Probable G-protein coupled receptor 83 OS=Homo sapiens GN=GPR83 PE=2 SV=2

Group 8l(b):

>sp|P21452|NK2R_HUMAN/1-398 Substance-K receptor OS=Homo sapiens GN=TACR2 PE=2 SV=3

>sp|P25103|NK1R_HUMAN/1-407 Substance-P receptor OS=Homo sapiens GN=TACR1 PE=1 SV=1

>sp|P29371|NK3R_HUMAN/1-465 Neuromedin-K receptor OS=Homo sapiens GN=TACR3 PE=2 SV=1

>sp|Q96P65|QRFPR_HUMAN/1-431 Pyroglutamylated RFamide peptide receptor OS=Homo sapiens GN=QRFPR PE=1 SV=2

>sp|O43613|OX1R_HUMAN/1-425 Orexin receptor type 1 OS=Homo sapiens GN=HCRTR1 PE=1 SV=2

>sp|O43614|OX2R_HUMAN/1-444 Orexin receptor type 2 OS=Homo sapiens GN=HCRTR2 PE=2 SV=2

>sp|Q9GZQ6|NPFF1_HUMAN/1-430 Neuropeptide FF receptor 1 OS=Homo sapiens GN=NPFFR1 PE=1 SV=1

>sp|Q9Y5X5|NPFF2_HUMAN/1-522 Neuropeptide FF receptor 2 OS=Homo sapiens GN=NPFFR2 PE=1 SV=2

Group 8m(a):

>sp|Q8NGU9|GP150_HUMAN/1-434 Probable G-protein coupled receptor 150 OS=Homo sapiens GN=GPR150 PE=2 SV=1

>sp|Q8IZ08|GP135_HUMAN/1-494 Probable G-protein coupled receptor 135 OS=Homo sapiens GN=GPR135 PE=2 SV=2

Group 8m(b):

>sp|Q9UHM6|OPN4_HUMAN/1-478 Melanopsin OS=Homo sapiens GN=OPN4 PE=1 SV=1

>sp|Q9H1Y3|OPN3_HUMAN/1-402 Opsin-3 OS=Homo sapiens GN=OPN3 PE=1 SV=1

Group 8n:

>sp|P41146|OPRX_HUMAN/1-370 Nociceptin receptor OS=Homo sapiens GN=OPRL1 PE=2 SV=1

>sp|P41143|OPRD_HUMAN/1-372 Delta-type opioid receptor OS=Homo sapiens GN=OPRD1 PE=1 SV=4

>sp|P35372|OPRM_HUMAN/1-400 Mu-type opioid receptor OS=Homo sapiens GN=OPRM1 PE=1 SV=2

>sp|P41145|OPRK_HUMAN/1-380 Kappa-type opioid receptor OS=Homo sapiens GN=OPRK1 PE=1 SV=2

>sp|P48145|NPBW1_HUMAN/1-328 Neuropeptides B/W receptor type 1 OS=Homo sapiens GN=NPBWR1 PE=1 SV=2

>sp|P48146|NPBW2_HUMAN/1-333 Neuropeptides B/W receptor type 2 OS=Homo sapiens GN=NPBWR2 PE=1 SV=2

>sp|P30872|SSR1_HUMAN/1-391 Somatostatin receptor type 1 OS=Homo sapiens GN=SSTR1 PE=1 SV=1

>sp|P31391|SSR4_HUMAN/1-388 Somatostatin receptor type 4 OS=Homo sapiens GN=SSTR4 PE=2 SV=2

>sp|P30874|SSR2_HUMAN/1-369 Somatostatin receptor type 2 OS=Homo sapiens GN=SSTR2 PE=1 SV=1

>sp|P32745|SSR3_HUMAN/1-418 Somatostatin receptor type 3 OS=Homo sapiens GN=SSTR3 PE=1 SV=1

>sp|P35346|SSR5_HUMAN/1-364 Somatostatin receptor type 5 OS=Homo sapiens GN=SSTR5 PE=1 SV=3

Group 8o(a):

>sp|Q96RI0|PAR4_HUMAN/1-385 Proteinase-activated receptor 4 OS=Homo sapiens GN=F2RL3 PE=1 SV=3

>sp|P25116|PAR1_HUMAN/1-425 Proteinase-activated receptor 1 OS=Homo sapiens GN=F2R PE=1 SV=2

>sp|P55085|PAR2_HUMAN/1-397 Proteinase-activated receptor 2 OS=Homo sapiens GN=F2RL1 PE=1 SV=1

>sp|O00254|PAR3_HUMAN/1-374 Proteinase-activated receptor 3 OS=Homo sapiens GN=F2RL2 PE=1 SV=1

Group 8o(b):

>sp|Q8TDS5|OXER1_HUMAN/1-423 Oxoeicosanoid receptor 1 OS=Homo sapiens GN=OXER1 PE=2 SV=1

>sp|Q99705|MCHR1_HUMAN/1-422 Melanin-concentrating hormone receptor 1 OS=Homo sapiens GN=MCHR1 PE=1 SV=2

>sp|Q8TDU9|RL3R2_HUMAN/1-374 Relaxin-3 receptor 2 OS=Homo sapiens GN=RXFP4 PE=1 SV=1

>sp|Q9NSD7|RL3R1_HUMAN/1-469 Relaxin-3 receptor 1 OS=Homo sapiens GN=RXFP3 PE=1 SV=1

Group 9a:

>sp|Q7Z5H4|VN1R5_HUMAN/1-357 Vomeronasal type-1 receptor 5 OS=Homo sapiens GN=VN1R5 PE=2 SV=2

>sp|Q8TDU5|VNRL4_HUMAN/1-208 Putative vomeronasal receptor-like protein 4 OS=Homo sapiens GN=VN1R17P PE=5 SV=1

>sp|Q8NGA4|GPC39_HUMAN/1-272 Putative G-protein coupled receptor GPCR39 OS=Homo sapiens PE=5 SV=2

>sp|O75388|GPR32_HUMAN/1-356 Probable G-protein coupled receptor 32 OS=Homo sapiens GN=GPR32 PE=2 SV=1

>sp|Q16570|DUFFY_HUMAN/1-336 Duffy antigen/chemokine receptor OS=Homo sapiens GN=DARC PE=1 SV=3

>sp|Q96P88|GNRR2_HUMAN/1-178 Putative gonadotropin-releasing hormone II receptor OS=Homo sapiens GN=GNRHR2 PE=5 SV=3

>sp|Q96CH1|GP146_HUMAN/1-333 Probable G-protein coupled receptor 146 OS=Homo sapiens GN=GPR146 PE=2 SV=1

Group 9b(a):

>sp|Q9NPC1|LT4R2_HUMAN/1-389 Leukotriene B4 receptor 2 OS=Homo sapiens GN=LTB4R2 PE=1 SV=1

>sp|Q15722|LT4R1_HUMAN/1-352 Leukotriene B4 receptor 1 OS=Homo sapiens GN=LTB4R PE=1 SV=2

Group 9b(b):

>sp|Q49SQ1|GPR33_HUMAN/1-333 Probable G-protein coupled receptor 33 OS=Homo sapiens GN=GPR33 PE=2 SV=1

>sp|P46091|GPR1_HUMAN/1-355 G-protein coupled receptor 1 OS=Homo sapiens GN=GPR1 PE=1 SV=2

>sp|Q99788|CML1_HUMAN/1-373 Chemokine-like receptor 1 OS=Homo sapiens GN=CMKLR1 PE=1 SV=2

>sp|Q9Y5Y4|PD2R2_HUMAN/1-395 Prostaglandin D2 receptor 2 OS=Homo sapiens GN=PTGDR2 PE=1 SV=3

>sp|P21462|FPR1_HUMAN/1-350 fMet-Leu-Phe receptor OS=Homo sapiens GN=FPR1 PE=1 SV=3

>sp|P25089|FPR3_HUMAN/1-353 N-formyl peptide receptor 3 OS=Homo sapiens GN=FPR3 PE=2 SV=2

>sp|P25090|FPR2_HUMAN/1-351 N-formyl peptide receptor 2 OS=Homo sapiens GN=FPR2 PE=2 SV=2

>sp|Q9P296|C5ARL_HUMAN/1-337 C5a anaphylatoxin chemotactic receptor C5L2 OS=Homo sapiens GN=GPR77 PE=2 SV=1

>sp|Q16581|C3AR_HUMAN/1-482 C3a anaphylatoxin chemotactic receptor OS=Homo sapiens GN=C3AR1 PE=1 SV=2

>sp|P21730|C5AR_HUMAN/1-350 C5a anaphylatoxin chemotactic receptor OS=Homo sapiens GN=C5AR1 PE=1 SV=2

Group 9c:

>sp|O00590|CCBP2_HUMAN/1-384 Chemokine-binding protein 2 OS=Homo sapiens GN=CCBP2 PE=2 SV=2

>sp|P49238|CX3C1_HUMAN/1-355 CX3C chemokine receptor 1 OS=Homo sapiens GN=CX3CR1 PE=1 SV=1

>sp|O00421|CCRL2_HUMAN/1-344 C-C chemokine receptor-like 2 OS=Homo sapiens GN=CCRL2 PE=1 SV=2

>sp|P51685|CCR8_HUMAN/1-355 C-C chemokine receptor type 8 OS=Homo sapiens GN=CCR8 PE=1 SV=1

>sp|P51679|CCR4_HUMAN/1-360 C-C chemokine receptor type 4 OS=Homo sapiens GN=CCR4 PE=1 SV=1

>sp|P41597|CCR2_HUMAN/1-374 C-C chemokine receptor type 2 OS=Homo sapiens GN=CCR2 PE=1 SV=1

>sp|P51681|CCR5_HUMAN/1-352 C-C chemokine receptor type 5 OS=Homo sapiens GN=CCR5 PE=1 SV=1

>sp|P32246|CCR1_HUMAN/1-355 C-C chemokine receptor type 1 OS=Homo sapiens GN=CCR1 PE=1 SV=1

>sp|P51677|CCR3_HUMAN/1-355 C-C chemokine receptor type 3 OS=Homo sapiens GN=CCR3 PE=1 SV=1

>sp|P61073|CXCR4_HUMAN/1-352 C-X-C chemokine receptor type 4 OS=Homo sapiens GN=CXCR4 PE=1 SV=1

>sp|P46092|CCR10_HUMAN/1-362 C-C chemokine receptor type 10 OS=Homo sapiens GN=CCR10 PE=1 SV=3

>sp|P32302|CXCR5_HUMAN/1-372 C-X-C chemokine receptor type 5 OS=Homo sapiens GN=CXCR5 PE=1 SV=1

>sp|Q9NPB9|CCRL1_HUMAN/1-350 C-C chemokine receptor type 11 OS=Homo sapiens GN=CCRL1 PE=1 SV=1

>sp|O00574|CXCR6_HUMAN/1-342 C-X-C chemokine receptor type 6 OS=Homo sapiens GN=CXCR6 PE=2 SV=1

>sp|P51686|CCR9_HUMAN/1-369 C-C chemokine receptor type 9 OS=Homo sapiens GN=CCR9 PE=1 SV=2

>sp|P49682|CXCR3_HUMAN/1-368 C-X-C chemokine receptor type 3 OS=Homo sapiens GN=CXCR3 PE=1 SV=2

>sp|P25024|CXCR1_HUMAN/1-350 C-X-C chemokine receptor type 1 OS=Homo sapiens GN=CXCR1 PE=1 SV=2

>sp|P25025|CXCR2_HUMAN/1-360 C-X-C chemokine receptor type 2 OS=Homo sapiens GN=CXCR2 PE=1 SV=2

>sp|P32248|CCR7_HUMAN/1-378 C-C chemokine receptor type 7 OS=Homo sapiens GN=CCR7 PE=1 SV=2

>sp|P51684|CCR6_HUMAN/1-374 C-C chemokine receptor type 6 OS=Homo sapiens GN=CCR6 PE=1 SV=2

Group 9d(a):

>sp|Q6W5P4|NPSR1_HUMAN/1-371 Neuropeptide S receptor OS=Homo sapiens GN=NPSR1 PE=2 SV=1

Group 9d(b):

>sp|P30518|V2R_HUMAN/1-371 Vasopressin V2 receptor OS=Homo sapiens GN=AVPR2 PE=1 SV=1

>sp|P37288|V1AR_HUMAN/1-418 Vasopressin V1a receptor OS=Homo sapiens GN=AVPR1A PE=1 SV=1

>sp|P30559|OXYR_HUMAN/1-389 Oxytocin receptor OS=Homo sapiens GN=OXTR PE=1 SV=2

>sp|P47901|V1BR_HUMAN/1-424 Vasopressin V1b receptor OS=Homo sapiens GN=AVPR1B PE=2 SV=1

Group 9e(a):

>sp|O43194|GPR39_HUMAN/1-453 G-protein coupled receptor 39 OS=Homo sapiens GN=GPR39 PE=1 SV=1

Group 9e(b):

>sp|Q9HB89|NMUR1_HUMAN/1-426 Neuromedin-U receptor 1 OS=Homo sapiens GN=NMUR1 PE=2 SV=1

>sp|Q9GZQ4|NMUR2_HUMAN/1-415 Neuromedin-U receptor 2 OS=Homo sapiens GN=NMUR2 PE=2 SV=2

>sp|Q92847|GHSR_HUMAN/1-366 Growth hormone secretagogue receptor type 1 OS=Homo sapiens GN=GHSR PE=1 SV=1

>sp|O43193|MTLR_HUMAN/1-412 Motilin receptor OS=Homo sapiens GN=MLNR PE=2 SV=1

>sp|O95665|NTR2_HUMAN/1-410 Neurotensin receptor type 2 OS=Homo sapiens GN=NTSR2 PE=2 SV=2

>sp|P30989|NTR1_HUMAN/1-418 Neurotensin receptor type 1 OS=Homo sapiens GN=NTSR1 PE=1 SV=2

Group 9f:

>sp|P48039|MTR1A_HUMAN/1-350 Melatonin receptor type 1A OS=Homo sapiens GN=MTNR1A PE=2 SV=1

>sp|P49286|MTR1B_HUMAN/1-362 Melatonin receptor type 1B OS=Homo sapiens GN=MTNR1B PE=2 SV=1

>sp|Q8TCW9|PKR1_HUMAN/1-393 Prokineticin receptor 1 OS=Homo sapiens GN=PROKR1 PE=2 SV=1

>sp|Q8NFJ6|PKR2_HUMAN/1-384 Prokineticin receptor 2 OS=Homo sapiens GN=PROKR2 PE=1 SV=1

>sp|P25101|EDNRA_HUMAN/1-427 Endothelin-1 receptor OS=Homo sapiens GN=EDNRA PE=1 SV=1

>sp|P24530|EDNRB_HUMAN/1-442 Endothelin B receptor OS=Homo sapiens GN=EDNRB PE=1 SV=1

>sp|P32247|BRS3_HUMAN/1-399 Bombesin receptor subtype-3 OS=Homo sapiens GN=BRS3 PE=1 SV=1

>sp|P30550|GRPR_HUMAN/1-384 Gastrin-releasing peptide receptor OS=Homo sapiens GN=GRPR PE=2 SV=1

>sp|P28336|NMBR_HUMAN/1-390 Neuromedin-B receptor OS=Homo sapiens GN=NMBR PE=2 SV=2

>sp|P49146|NPY2R_HUMAN/1-381 Neuropeptide Y receptor type 2 OS=Homo sapiens GN=NPY2R PE=2 SV=1

>sp|P49683|PRLHR_HUMAN/1-370 Prolactin-releasing peptide receptor OS=Homo sapiens GN=PRLHR PE=1 SV=3

>sp|P50391|NPY4R_HUMAN/1-375 Neuropeptide Y receptor type 4 OS=Homo sapiens GN=PPYR1 PE=2 SV=1

>sp|P25929|NPY1R_HUMAN/1-384 Neuropeptide Y receptor type 1 OS=Homo sapiens GN=NPY1R PE=1 SV=1

>sp|Q99463|NPY6R_HUMAN/1-290 Putative neuropeptide Y receptor type 6 OS=Homo sapiens GN=NPY6R PE=5 SV=1

Group 9g(a):

>sp|Q8TDV0|GP151_HUMAN/1-419 Probable G-protein coupled receptor 151 OS=Homo sapiens GN=GPR151 PE=2 SV=1

Group 9g(b):

>sp|Q9UKP6|UR2R_HUMAN/1-389 Urotensin-2 receptor OS=Homo sapiens GN=UTS2R PE=1 SV=1

>sp|Q969F8|KISSR_HUMAN/1-398 KiSS-1 receptor OS=Homo sapiens GN=KISS1R PE=1 SV=2

>sp|P47211|GALR1_HUMAN/1-349 Galanin receptor type 1 OS=Homo sapiens GN=GALR1 PE=2 SV=3

>sp|O43603|GALR2_HUMAN/1-387 Galanin receptor type 2 OS=Homo sapiens GN=GALR2 PE=1 SV=1

>sp|O60755|GALR3_HUMAN/1-368 Galanin receptor type 3 OS=Homo sapiens GN=GALR3 PE=2 SV=1

Group 9h:

>sp|Q7Z602|GP141_HUMAN/1-305 Probable G-protein coupled receptor 141 OS=Homo sapiens GN=GPR141 PE=2 SV=1

>sp|Q96P67|GPR82_HUMAN/1-336 Probable G-protein coupled receptor 82 OS=Homo sapiens GN=GPR82 PE=2 SV=1

>sp|O00270|GPR31_HUMAN/1-319 12-(S)-hydroxy-5,8,10,14-eicosatetraenoic acid receptor OS=Homo sapiens GN=GPR31 PE=2 SV=2

>sp|Q9BXC0|HCAR1_HUMAN/1-346 Hydroxycarboxylic acid receptor 1 OS=Homo sapiens GN=HCAR1 PE=1 SV=1

>sp|Q8TDS4|HCAR2_HUMAN/1-363 Hydroxycarboxylic acid receptor 2 OS=Homo sapiens GN=HCAR2 PE=1 SV=1

>sp|P49019|HCAR3_HUMAN/1-387 Hydroxycarboxylic acid receptor 3 OS=Homo sapiens GN=HCAR3 PE=1 SV=2

>sp|O14626|GP171_HUMAN/1-319 Probable G-protein coupled receptor 171 OS=Homo sapiens GN=GPR171 PE=2 SV=1

>sp|Q9BY21|GPR87_HUMAN/1-358 G-protein coupled receptor 87 OS=Homo sapiens GN=GPR87 PE=2 SV=1

>sp|Q15391|P2Y14_HUMAN/1-338 P2Y purinoceptor 14 OS=Homo sapiens GN=P2RY14 PE=1 SV=1

>sp|Q9H244|P2Y12_HUMAN/1-342 P2Y purinoceptor 12 OS=Homo sapiens GN=P2RY12 PE=1 SV=1

>sp|Q9BPV8|P2Y13_HUMAN/1-354 P2Y purinoceptor 13 OS=Homo sapiens GN=P2RY13 PE=2 SV=3

Group 9i:

>sp|O14842|FFAR1_HUMAN/1-300 Free fatty acid receptor 1 OS=Homo sapiens GN=FFAR1 PE=2 SV=1

>sp|O15552|FFAR2_HUMAN/1-330 Free fatty acid receptor 2 OS=Homo sapiens GN=FFAR2 PE=2 SV=1

>sp|O14843|FFAR3_HUMAN/1-346 Free fatty acid receptor 3 OS=Homo sapiens GN=FFAR3 PE=1 SV=1

>sp|O15529|GPR42_HUMAN/1-346 G-protein coupled receptor 42 OS=Homo sapiens GN=GPR42 PE=1 SV=1

>sp|Q8IYL9|PSYR_HUMAN/1-337 Psychosine receptor OS=Homo sapiens GN=GPR65 PE=1 SV=1

>sp|Q15743|OGR1_HUMAN/1-365 Ovarian cancer G-protein coupled receptor 1 OS=Homo sapiens GN=GPR68 PE=1 SV=1

>sp|P46093|GPR4_HUMAN/1-362 G-protein coupled receptor 4 OS=Homo sapiens GN=GPR4 PE=2 SV=2

>sp|P25105|PTAFR_HUMAN/1-342 Platelet-activating factor receptor OS=Homo sapiens GN=PTAFR PE=1 SV=1

Group 9j:

>sp|Q9Y2T6|GPR55_HUMAN/1-319 G-protein coupled receptor 55 OS=Homo sapiens GN=GPR55 PE=1 SV=2

>sp|Q9HC97|GPR35_HUMAN/1-309 G-protein coupled receptor 35 OS=Homo sapiens GN=GPR35 PE=2 SV=4

>sp|Q99677|LPAR4_HUMAN/1-370 Lysophosphatidic acid receptor 4 OS=Homo sapiens GN=LPAR4 PE=1 SV=1

>sp|P43657|LPAR6_HUMAN/1-344 Lysophosphatidic acid receptor 6 OS=Homo sapiens GN=LPAR6 PE=1 SV=3

>sp|Q9BXC1|GP174_HUMAN/1-333 Probable G-protein coupled receptor 174 OS=Homo sapiens GN=GPR174 PE=2 SV=1

>sp|O00398|P2Y10_HUMAN/1-339 Putative P2Y purinoceptor 10 OS=Homo sapiens GN=P2RY10 PE=2 SV=1

Group 9k:

>sp|O15218|GP182_HUMAN/1-404 G-protein coupled receptor 182 OS=Homo sapiens GN=GPR182 PE=2 SV=1

>sp|P25106|CXCR7_HUMAN/1-362 C-X-C chemokine receptor type 7 OS=Homo sapiens GN=CXCR7 PE=1 SV=3

>sp|Q9UNW8|GP132_HUMAN/1-380 Probable G-protein coupled receptor 132 OS=Homo sapiens GN=GPR132 PE=2 SV=1

>sp|Q9H1C0|LPAR5_HUMAN/1-372 Lysophosphatidic acid receptor 5 OS=Homo sapiens GN=LPAR5 PE=2 SV=1

Group 9l:

>sp|Q96P68|OXGR1_HUMAN/1-337 2-oxoglutarate receptor 1 OS=Homo sapiens GN=OXGR1 PE=2 SV=1

>sp|P47900|P2RY1_HUMAN/1-373 P2Y purinoceptor 1 OS=Homo sapiens GN=P2RY1 PE=1 SV=1

>sp|Q15077|P2RY6_HUMAN/1-328 P2Y purinoceptor 6 OS=Homo sapiens GN=P2RY6 PE=1 SV=1

>sp|P41231|P2RY2_HUMAN/1-377 P2Y purinoceptor 2 OS=Homo sapiens GN=P2RY2 PE=1 SV=4

>sp|P51582|P2RY4_HUMAN/1-365 P2Y purinoceptor 4 OS=Homo sapiens GN=P2RY4 PE=1 SV=1

Group 10a:

>sp|Q9H3N8|HRH4_HUMAN/1-390 Histamine H4 receptor OS=Homo sapiens GN=HRH4 PE=1 SV=2

>sp|Q9Y5N1|HRH3_HUMAN/1-445 Histamine H3 receptor OS=Homo sapiens GN=HRH3 PE=1 SV=2

>sp|P25021|HRH2_HUMAN/1-359 Histamine H2 receptor OS=Homo sapiens GN=HRH2 PE=2 SV=1

>sp|P35367|HRH1_HUMAN/1-487 Histamine H1 receptor OS=Homo sapiens GN=HRH1 PE=1 SV=1

Group 10b:

>sp|P08172|ACM2_HUMAN/1-466 Muscarinic acetylcholine receptor M2 OS=Homo sapiens GN=CHRM2 PE=1 SV=1

>sp|P08173|ACM4_HUMAN/1-479 Muscarinic acetylcholine receptor M4 OS=Homo sapiens GN=CHRM4 PE=1 SV=2

>sp|P20309|ACM3_HUMAN/1-590 Muscarinic acetylcholine receptor M3 OS=Homo sapiens GN=CHRM3 PE=1 SV=1

>sp|P11229|ACM1_HUMAN/1-460 Muscarinic acetylcholine receptor M1 OS=Homo sapiens GN=CHRM1 PE=1 SV=2

>sp|P08912|ACM5_HUMAN/1-532 Muscarinic acetylcholine receptor M5 OS=Homo sapiens GN=CHRM5 PE=2 SV=2

Group 10c:

>sp|P18089|ADA2B_HUMAN/1-450 Alpha-2B adrenergic receptor OS=Homo sapiens GN=ADRA2B PE=2 SV=2

>sp|P08913|ADA2A_HUMAN/1-450 Alpha-2A adrenergic receptor OS=Homo sapiens GN=ADRA2A PE=1 SV=3

>sp|P18825|ADA2C_HUMAN/1-462 Alpha-2C adrenergic receptor OS=Homo sapiens GN=ADRA2C PE=2 SV=2

>sp|P35348|ADA1A_HUMAN/1-466 Alpha-1A adrenergic receptor OS=Homo sapiens GN=ADRA1A PE=2 SV=2

>sp|P35368|ADA1B_HUMAN/1-520 Alpha-1B adrenergic receptor OS=Homo sapiens GN=ADRA1B PE=2 SV=3

>sp|P25100|ADA1D_HUMAN/1-572 Alpha-1D adrenergic receptor OS=Homo sapiens GN=ADRA1D PE=2 SV=2

>sp|P07550|ADRB2_HUMAN/1-413 Beta-2 adrenergic receptor OS=Homo sapiens GN=ADRB2 PE=1 SV=3

>sp|P08588|ADRB1_HUMAN/1-477 Beta-1 adrenergic receptor OS=Homo sapiens GN=ADRB1 PE=1 SV=2

>sp|P13945|ADRB3_HUMAN/1-408 Beta-3 adrenergic receptor OS=Homo sapiens GN=ADRB3 PE=2 SV=3

Group 10d:

>sp|P21728|DRD1_HUMAN/1-446 D(1A) dopamine receptor OS=Homo sapiens GN=DRD1 PE=1 SV=1

>sp|P21918|DRD5_HUMAN/1-477 D(1B) dopamine receptor OS=Homo sapiens GN=DRD5 PE=1 SV=2

>sp|P21917|DRD4_HUMAN/1-467 D(4) dopamine receptor OS=Homo sapiens GN=DRD4 PE=1 SV=2

>sp|P35462|DRD3_HUMAN/1-400 D(3) dopamine receptor OS=Homo sapiens GN=DRD3 PE=1 SV=2

>sp|P14416|DRD2_HUMAN/1-443 D(2) dopamine receptor OS=Homo sapiens GN=DRD2 PE=1 SV=2

Group 10e:

>sp|P41595|5HT2B_HUMAN/1-481 5-hydroxytryptamine receptor 2B OS=Homo sapiens GN=HTR2B PE=1 SV=1

>sp|P28223|5HT2A_HUMAN/1-471 5-hydroxytryptamine receptor 2A OS=Homo sapiens GN=HTR2A PE=1 SV=2

>sp|P28335|5HT2C_HUMAN/1-458 5-hydroxytryptamine receptor 2C OS=Homo sapiens GN=HTR2C PE=1 SV=1

>sp|P50406|5HT6R_HUMAN/1-440 5-hydroxytryptamine receptor 6 OS=Homo sapiens GN=HTR6 PE=1 SV=1

>sp|P47898|5HT5A_HUMAN/1-357 5-hydroxytryptamine receptor 5A OS=Homo sapiens GN=HTR5A PE=1 SV=1

>sp|P34969|5HT7R_HUMAN/1-479 5-hydroxytryptamine receptor 7 OS=Homo sapiens GN=HTR7 PE=1 SV=2

>sp|Q13639|5HT4R_HUMAN/1-388 5-hydroxytryptamine receptor 4 OS=Homo sapiens GN=HTR4 PE=1 SV=2

>sp|P08908|5HT1A_HUMAN/1-422 5-hydroxytryptamine receptor 1A OS=Homo sapiens GN=HTR1A PE=1 SV=3

>sp|P28222|5HT1B_HUMAN/1-390 5-hydroxytryptamine receptor 1B OS=Homo sapiens GN=HTR1B PE=1 SV=1

>sp|P28221|5HT1D_HUMAN/1-377 5-hydroxytryptamine receptor 1D OS=Homo sapiens GN=HTR1D PE=2 SV=1

>sp|P28566|5HT1E_HUMAN/1-365 5-hydroxytryptamine receptor 1E OS=Homo sapiens GN=HTR1E PE=2 SV=1

>sp|P30939|5HT1F_HUMAN/1-366 5-hydroxytryptamine receptor 1F OS=Homo sapiens GN=HTR1F PE=2 SV=1

Group 11a(a):

>sp|Q99680|GPR22_HUMAN/1-433 Probable G-protein coupled receptor 22 OS=Homo sapiens GN=GPR22 PE=2 SV=2

>sp|P34981|TRFR_HUMAN/1-398 Thyrotropin-releasing hormone receptor OS=Homo sapiens GN=TRHR PE=1 SV=1

>sp|Q9GZN0|GPR88_HUMAN/1-384 Probable G-protein coupled receptor 88 OS=Homo sapiens GN=GPR88 PE=2 SV=2

>sp|Q5NUL3|O3FA1_HUMAN/1-377 Omega-3 fatty acid receptor 1 OS=Homo sapiens GN=O3FAR1 PE=1 SV=2

Group 11a(b):

>sp|Q9Y5Y3|GPR45_HUMAN/1-372 Probable G-protein coupled receptor 45 OS=Homo sapiens GN=GPR45 PE=2 SV=2

>sp|Q9BZJ6|GPR63_HUMAN/1-419 Probable G-protein coupled receptor 63 OS=Homo sapiens GN=GPR63 PE=2 SV=2

Group 11a(c):

>sp|Q96P66|GP101_HUMAN/1-508 Probable G-protein coupled receptor 101 OS=Homo sapiens GN=GPR101 PE=2 SV=1

>sp|Q15761|NPY5R_HUMAN/1-445 Neuropeptide Y receptor type 5 OS=Homo sapiens GN=NPY5R PE=2 SV=2

>sp|Q9NS67|GPR27_HUMAN/1-375 Probable G-protein coupled receptor 27 OS=Homo sapiens GN=GPR27 PE=2 SV=1

>sp|Q9NS66|GP173_HUMAN/1-373 Probable G-protein coupled receptor 173 OS=Homo sapiens GN=GPR173 PE=2 SV=1

>sp|P60893|GPR85_HUMAN/1-370 Probable G-protein coupled receptor 85 OS=Homo sapiens GN=GPR85 PE=2 SV=1

>sp|P32238|CCKAR_HUMAN/1-428 Cholecystokinin receptor type A OS=Homo sapiens GN=CCKAR PE=1 SV=1

>sp|P32239|GASR_HUMAN/1-447 Gastrin/cholecystokinin type B receptor OS=Homo sapiens GN=CCKBR PE=1 SV=1

>sp|Q9NQS5|GPR84_HUMAN/1-396 G-protein coupled receptor 84 OS=Homo sapiens GN=GPR84 PE=2 SV=1

Group 11b:

>sp|P46663|BKRB1_HUMAN/1-353 B1 bradykinin receptor OS=Homo sapiens GN=BDKRB1 PE=1 SV=3

>sp|P30411|BKRB2_HUMAN/1-391 B2 bradykinin receptor OS=Homo sapiens GN=BDKRB2 PE=1 SV=2

>sp|P50052|AGTR2_HUMAN/1-363 Type-2 angiotensin II receptor OS=Homo sapiens GN=AGTR2 PE=1 SV=1

>sp|P30556|AGTR1_HUMAN/1-359 Type-1 angiotensin II receptor OS=Homo sapiens GN=AGTR1 PE=1 SV=1

>sp|O00155|GPR25_HUMAN/1-361 Probable G-protein coupled receptor 25 OS=Homo sapiens GN=GPR25 PE=2 SV=2

>sp|P49685|GPR15_HUMAN/1-360 G-protein coupled receptor 15 OS=Homo sapiens GN=GPR15 PE=2 SV=1

Group 11c:

>sp|Q969V1|MCHR2_HUMAN/1-340 Melanin-concentrating hormone receptor 2 OS=Homo sapiens GN=MCHR2 PE=1 SV=1

>sp|P35414|APJ_HUMAN/1-380 Apelin receptor OS=Homo sapiens GN=APLNR PE=1 SV=1

>sp|Q9BXA5|SUCR1_HUMAN/1-334 Succinate receptor 1 OS=Homo sapiens GN=SUCNR1 PE=1 SV=2

>sp|Q96G91|P2Y11_HUMAN/1-374 P2Y purinoceptor 11 OS=Homo sapiens GN=P2RY11 PE=2 SV=2

>sp|P46094|XCR1_HUMAN/1-333 Chemokine XC receptor 1 OS=Homo sapiens GN=XCR1 PE=1 SV=1

>sp|Q99527|GPER_HUMAN/1-375 G-protein coupled estrogen receptor 1 OS=Homo sapiens GN=GPER PE=2 SV=1

>sp|Q14330|GPR18_HUMAN/1-331 N-arachidonyl glycine receptor OS=Homo sapiens GN=GPR18 PE=2 SV=2

>sp|Q9UPC5|GPR34_HUMAN/1-381 Probable G-protein coupled receptor 34 OS=Homo sapiens GN=GPR34 PE=1 SV=2

>sp|Q99678|GPR20_HUMAN/1-358 G-protein coupled receptor 20 OS=Homo sapiens GN=GPR20 PE=1 SV=2

>sp|Q86VZ1|P2RY8_HUMAN/1-359 P2Y purinoceptor 8 OS=Homo sapiens GN=P2RY8 PE=1 SV=1

>sp|P32249|GP183_HUMAN/1-361 G-protein coupled receptor 183 OS=Homo sapiens GN=GPR183 PE=1 SV=3

>sp|Q13304|GPR17_HUMAN/1-367 Uracil nucleotide/cysteinyl leukotriene receptor OS=Homo sapiens GN=GPR17 PE=2 SV=2

>sp|Q9Y271|CLTR1_HUMAN/1-337 Cysteinyl leukotriene receptor 1 OS=Homo sapiens GN=CYSLTR1 PE=2 SV=1

>sp|Q9NS75|CLTR2_HUMAN/1-346 Cysteinyl leukotriene receptor 2 OS=Homo sapiens GN=CYSLTR2 PE=2 SV=1

Protein structure (receptor name) used to determine 7TM domain:

- group 1: GRM1
- group 2: SMO
- group 3: GRM1
- group 4a: SMO
- group 4b: CRFR1
- group 5a: AA2AR
- group 5b: SMO
- group 6a: SMO
- group 6b: GLR
- group 6c: CRFR1
- group 7: OPSD (chosen because of experimental evidence [65])
- group 8a(a): AA2AR
- group 8a(b): S1PR1
- group 8a(c): GLR
- group 8b(a): P2Y12
- group 8b(b): OPSD
- group 8c(a): OPSD
- group 8c(b): AA2AR
- group 8d: OPSD
- group 8e: OPSD
- group 8f(a): AA2A
- group 8f(b): NTR1
- group 8f(c): S1PR1
- (group 8g: missing in naming)
- group 8h: OPRD
- group 8i: OPSD
- group 8j: AA2AR
- group 8k: AA2AR
- group 8l(a): NTR1
- group 8l(b): AA2A
- group 8m(a): S1PR1
- group 8m(b): OPSD
- group 8n: OPRD
- group 8o(a): PAR1
- group 8o(b): NTR1
- group 9a: P2Y12
- group 9b(a): NTR1
- group 9b(b): CXCR4
- group 9c: CXCR4
- group 9d(a): NTR1
- group 9d(b): OPRD
- group 9e(a): AA2AR
- group 93(b): NTR1
- group 9f: CXCR4
- group 9g(a): S1PR1
- group 9g(b): OPRD
- group 9h: P2Y12
- group 9i: P2Y12
- group 9j: OPRD
- group 9k: CXCR4
- group 9l: P2Y12
- group 10a: H1HR
- group 10b: ACM2
- group 10c: ADRB2
- group 10d: D3DR
- group 10e: 5HT1B
- group 11a(a): OPSD
- group 11a(b): P2Y12
- group 11a(c): D3DR
- group 11b: CXCR4
- group 11c: P2Y12
