# Supplementary material for: Sequence, Structure and Ligand Binding Evolution of Rhodopsin-Like G Protein-Coupled Receptors: A Crystal Structure-Based Phylogenetic Analysis
Source: PLoS One. 2015 Apr 16;10(4):e0123533. doi: 10.1371/journal.pone.0123533 (PMC4399913; doi:10.1371/journal.pone.0123533)
Supplement: S3 File — (DOCX) [file pone.0123533.s010.docx]

(GRM7:0.11916453,GRM8:0.05321355,(GRM4:0.12749033,(GRM6:0.16908675,((GRM2:0.02390221,GRM3:1.61824633)427:0.55749940,((GRM5:0.67022560,GRM1:0.21498798)491:0.44580182,((GPC6A:1.58543938,CASR:0.90052217)359:0.19129964,(((((((CALCR:0.33214429,CALRL:0.32590436)510:1.17065710,((CRFR1:0.42705178,CRFR2:0.16938352)502:0.86286678,((GLP2R:0.84052981,(GLR:0.66814803,(GLP1R:0.59915983,GIPR:0.61374072)359:0.20155186)476:0.32187304)372:0.15313861,((PTH1R:0.35307092,PTH2R:0.68978748)509:0.50035049,(VIPR2:0.53082021,((SCTR:0.51722426,PACR:0.46113839)362:0.15410372,(VIPR1:0.37381825,GHRHR:1.11281761)318:0.19547668)266:0.12113782)309:0.13624648)314:0.13258197)450:0.52260567)277:0.28045995)387:0.68479320,(GP126:2.10312408,(GP144:1.76456865,(EMR1:1.74305200,(GP110:0.69087476,GP116:0.76076677)508:0.79783192)342:0.11480629)404:0.47784099)472:0.73441303)288:0.67675569,(((FZD3:0.21917942,FZD6:0.26657374)356:0.14367664,((FZD2:0.06135186,(FZD1:0.10654143,FZD7:0.09689937)305:0.02746229)454:0.11554955,((FZD4:0.33734197,(FZD10:0.17440336,FZD9:0.24585550)508:0.25934947)481:0.27607313,(SMO:2.46679920,(FZD5:0.21649077,FZD8:0.24882907)403:0.14588916)248:0.19075052)243:0.17905993)278:0.39561457)486:1.75530700,(TS1R2:4.00818154,GPC5B:2.67099020)170:2.14837627)16:0.32505004)44:0.39515544,(((((((ELTD1:0.55717786,(GP114:2.29760134,GP128:2.70712435)175:0.71567256)72:0.38349107,((LPHN3:0.22133330,(LPHN1:0.20634644,LPHN2:0.12292653)510:0.31725144)480:0.39702612,(GPR64:1.84421809,(BAI1:0.29424086,(BAI3:0.19571112,BAI2:0.30039040)418:0.18165756)510:1.42848712)191:0.33083433)143:0.28628666)27:0.18485592,((EMR4:0.42978371,(CD97:0.82706547,EMR2:0.42773377)427:0.38292891)264:0.17502567,(GPR97:1.90904718,EMR3:0.88081100)127:0.21437985)197:0.40878929)62:0.18517968,(GP125:0.68942910,GP124:0.56829584)510:1.55240326)339:2.27330858,((GP158:1.99990508,(GABR2:1.07771325,GABR1:2.01073794)430:0.95411244)380:2.02932219,(GP142:2.56912399,((((((ADRB2_CORR:0.65313559,ADRB1_CORR:0.46751276)508:0.79865048,(5HT1B_CORR:0.76913679,HRH1_CORRH:2.58485790)155:0.14429071)198:0.28234966,(5HT2B_CORR:1.63186685,(DRD3_CORRH:1.37724487,(ACM3_CORRH:1.12548541,ACM2_CORRH:0.38324562)498:1.06559943)294:0.27988159)195:0.22066511)235:0.38695708,(AA2AR_CORR:1.25259144,(S1PR1_CORR:1.33727847,(ACTHR:1.01443357,((MC3R:0.29914000,MC4R:0.33276490)325:0.11582184,(MSHR:0.90843306,MC5R:0.28893228)504:0.24009123)481:0.43461313)504:0.59920576)348:0.31856472)102:0.09431337)154:0.33142204,((OPSD_CORRH:1.58146214,(LGR5:0.78853800,(LGR6:0.54818946,LGR4:1.53567958)437:0.35917228)505:1.83799896)257:0.51947298,(NTR1_CORRH:1.43011097,(GPR37:0.54882493,ETBR2:0.46318953)506:1.57108866)296:0.32010186)99:0.24882344)90:0.36744650,(((OPRM_CORRH:0.22076610,OPRD_CORRH:0.20292759)504:0.35274847,(OPRX_CORRH:0.45623035,OPRK_CORRH:0.21508639)457:0.17656294)496:0.79383745,(CXCR4_CORR:1.41140559,(CCR5_CORRH:1.37317737,(P2Y12_CORR:1.63004417,PAR1_CORRH:1.45739551)315:0.25918683)428:0.46606301)323:0.30950803)157:0.20583658)41:0.08985815)159:0.36784131)34:0.25698328)7:0.00010691,((GP113:4.55786112,GPR56:4.55761580)236:0.98319048,(GP111:1.68693325,GP115:1.99980784)444:2.08461633)108:0.43313406)12:0.22660433,(((GPC5C:2.45511403,(GP180:3.66891492,((GP156:4.55786103,GP149:3.54535223)335:1.31383359,(GP133:4.17237943,GP123:2.98471916)207:1.06135433)59:0.28117008)34:0.25036122)31:0.22734613,(TS1R3:3.53804277,TS1R1:4.55786335)202:1.84437059)21:0.42262391,((RXFP2:4.54160150,RXFP1:0.00010840)494:2.54278923,(TSHR:1.61013287,(FSHR:0.48237689,LSHR:0.42193658)262:0.19926712)495:1.61051426)466:2.49663675)16:0.37294325)4:0.19193847)17:0.20574344,((TA2R5:1.55009138,(T2R38:1.84443156,(((TA2R7:0.74020674,TA2R9:0.54977129)205:0.14395901,(((T2R14:0.80426414,(T2R13:0.51975194,(T2R20:0.17875038,(T2R19:0.35522102,(T2R50:0.32587491,(T2R30:0.08855168,(T2R46:0.06004034,(T2R45:0.11594211,(T2R31:0.07187586,T2R43:0.04488317)464:0.03341666)401:0.02669742)323:0.01804342)496:0.12173257)437:0.05022374)396:0.03978587)502:0.45918427)260:0.10359837)497:0.35386859,(T2R12:0.86190728,TA2R3:0.98188091)179:0.06130932)249:0.13783183,(TA2R8:0.58227083,(T2R10:0.84072992,T2R42:1.54392483)104:0.01827407)229:0.24493082)81:0.07129918)265:0.33788428,(TA2R1:1.24426857,((TA2R4:1.17045525,(T2R39:0.35168993,T2R40:1.16404066)509:0.44576303)452:0.35132782,(T2R16:1.22334938,(T2R41:0.75071021,T2R60:1.08333131)361:0.21045372)485:0.47042677)291:0.13114523)138:0.15770055)108:0.14829513)207:0.25839892)440:0.79202146,((VN1R1:1.01495502,(VN1R4:0.33448267,(VN1R3:0.51554050,VN1R2:0.43731706)291:0.24828230)185:0.12570252)510:1.65703465,(VNRL4:0.79525310,VN1R5:1.06130043)495:1.36823492)220:0.46775613)138:0.34582086)76:0.36567939,(GPC5D:1.67539528,RAI3:3.90401448)420:1.27001036)468:1.73813206)479:1.06302310)407:0.38024432)510:0.45803858)340:0.08968951)397:0.05765751);
