# Supplementary material for: Sequence, Structure and Ligand Binding Evolution of Rhodopsin-Like G Protein-Coupled Receptors: A Crystal Structure-Based Phylogenetic Analysis
Source: PLoS One. 2015 Apr 16;10(4):e0123533. doi: 10.1371/journal.pone.0123533 (PMC4399913; doi:10.1371/journal.pone.0123533)
Supplement: S4 File — (DOCX) [file pone.0123533.s011.docx]

(HCAR2:0.02044656,HCAR3:0.03475816,(HCAR1:0.45537036,((OXER1:0.75948663,GPR31:1.20468883)187:0.07396273,(GPR20:0.87149579,((((GP183:1.03880974,((GP171:1.63230398,(P2Y14:0.56159737,(GPR87:0.54182367,(P2Y12:0.41370292,P2Y13:0.53162380)492:0.24843884)354:0.04753263)488:0.30604984)381:0.23761718,(GPR82:1.79664223,GPR34:0.75208812)182:0.15927518)221:0.20297661)61:0.20756858,((((GPER:0.99435319,GP143:7.43619338)27:0.35656970,(((((UR2R:1.71040165,(KISSR:1.23378263,(GP151:2.49650246,(GALR1:0.69612607,(GALR2:0.19964964,GALR3:0.41627624)515:0.46246781)237:0.05009258)308:0.12634780)265:0.17635043)105:0.11877141,((NPSR1:1.17870808,(V2R:0.61402444,(V1AR:0.61489947,(OXYR:0.44378710,V1BR:0.45486669)333:0.13152418)297:0.13394945)488:0.37863856)331:0.17173928,(GNRHR:1.16233507,(GNRR2:0.77499467,GP150:3.85250075)257:0.25016138)200:0.26047977)301:0.52564550)9:0.05929224,((((BRS3:0.22803413,(NMBR:0.54540452,GRPR:0.33849548)359:0.09927701)523:0.44187009,(EDNRA:0.40345507,EDNRB:0.43000670)528:0.75836383)467:0.58558287,((TRFR:1.30168543,GP139:1.87124332)154:0.13055147,((NMUR1:0.44996078,NMUR2:0.45897956)525:0.42878455,((GHSR:0.60417567,MTLR:0.42433103)519:0.46157446,(GPR39:1.43873883,(NTR2:0.70923664,NTR1:0.55647928)521:0.41265344)251:0.08423729)292:0.10332042)428:0.27228106)248:0.24239605)36:0.17198399,(((PKR1:0.09969299,PKR2:0.16482048)528:1.36735752,(((GPR22:1.69843028,(GP176:2.16085817,O3FA1:1.36891637)54:0.00015370)39:0.10983725,(GPR45:0.52104474,GPR63:0.46461286)528:0.81418547)39:0.16259077,(((((((GP148:6.90861929,GP119:1.26724638)56:0.45224616,(((GPR12:0.22724173,(GPR6:0.24872905,GPR3:0.64242344)408:0.21139485)525:0.98151631,(ACTHR:0.60067406,(MSHR:0.76595154,(MC5R:0.39687069,(MC3R:0.22460604,MC4R:0.27790123)144:0.03573058)303:0.05870594)396:0.21980843)521:0.52342980)227:0.14281331,((CNR1:0.61781953,CNR2:0.87437586)506:0.70788769,((LPAR1:0.43002963,(LPAR3:0.41012051,LPAR2:0.57848481)521:0.33290524)518:0.36878709,((S1PR3:0.56286107,(S1PR5:0.58026703,S1PR2:0.64996385)421:0.20000359)308:0.11439300,(S1PR1:0.58444697,S1PR4:0.85489937)272:0.10124695)374:0.20949362)469:0.36462531)103:0.09457989)271:0.25438816)11:0.11377486,(GPR19:1.69227942,((AA2AR:0.43261985,AA2BR:0.41540215)397:0.09374027,(AA3R:0.72114438,AA1R:0.46197600)416:0.19974108)526:0.67869917)76:0.14582833)0:0.03990160,((HRH1:1.14279834,(((ACM4:0.44799234,ACM2:0.29555960)477:0.25177044,(ACM1:0.30968933,(ACM3:0.33002826,ACM5:0.73899016)262:0.14395728)414:0.16765235)525:0.74681586,(HRH4:0.94104966,HRH3:0.49666636)520:0.51310114)240:0.15197817)142:0.15420047,(((((HRH2:0.80196548,((ADA2C:0.30890318,(ADA2B:0.31156675,ADA2A:0.30892502)478:0.16352833)528:0.60278679,(DRD4:0.87184716,(DRD3:0.32790561,DRD2:0.38089412)526:0.42094176)363:0.19495357)89:0.09405513)67:0.04586351,(DRD1:0.25302083,DRD5:0.52297738)516:0.47600292)65:0.03977039,(ADA1A:0.25429347,(ADA1B:0.51313778,ADA1D:0.35138487)503:0.16554159)527:0.49297445)59:0.06826775,(n5HT7R:0.77381876,(n5HT5A:0.87305733,(n5HT1A:0.59285099,(n5HT1E:0.80881314,(n5HT1F:0.33878813,(n5HT1B:0.26050442,n5HT1D:0.24898422)476:0.28373755)242:0.15627816)361:0.16008145)300:0.18097587)188:0.09744813)204:0.21192268)38:0.07007542,((n5HT6R:1.21795927,(n5HT2B:0.61353648,(n5HT2A:0.43377108,n5HT2C:0.28761711)242:0.06853192)525:0.70857170)154:0.19429152,((ADRB2:0.38555571,(ADRB3:0.52848447,ADRB1:0.22041855)216:0.08387991)527:0.53280361,(n5HT4R:0.68692552,((TAAR1:0.41287557,(TAAR2:0.35966083,TAAR3:0.74618631)495:0.27257260)370:0.19261959,(TAAR5:0.91096310,(TAAR9:0.21543269,(TAAR6:0.12943306,TAAR8:0.16775059)499:0.17675322)528:0.49701170)434:0.18102353)522:0.39919084)440:0.19000113)77:0.06849589)23:0.07255822)44:0.08935770)132:0.27821474)15:0.18629917,((GP160:8.24364215,((((PE2R4:1.47589743,(PE2R3:1.05634278,(GP157:7.57991960,(TA2R:0.58539147,(PF2R:0.94450964,PE2R1:0.89032663)427:0.27018330)448:0.22100787)170:0.17272445)170:0.19533227)93:0.12130258,(PD2R:1.03719730,(PI2R:0.55476550,PE2R2:0.64551440)392:0.20029422)523:0.51868001)130:0.22427753,(GLRCORR:6.82605650,CRFR1CORR:8.24378548)122:0.47730763)39:0.31659577,(GP146:4.22335997,(GRM1CORRH:6.22840543,((GP153:0.43448178,GP162:0.55871100)527:4.45007789,(((((OR6F1:0.38519011,(OR6X1:0.49185073,((OR6S1:0.36061101,(OR6V1:0.46393143,(OR9A4:0.10058115,(OR9A1:0.18097351,OR9A2:0.09866075)377:0.04569512)526:0.43325076)450:0.13948493)223:0.09482262,((OR6M1:0.32824983,OR6J1:0.34854415)226:0.03996077,(OR6T1:0.37606107,((O2AP1:0.15316709,OR6C4:0.06023979)528:0.12814534,(((OR6C6:0.15460098,O6C70:0.14443139)449:0.06096177,(O6C74:0.16402833,((O6C68:0.20694140,OR6C2:0.09088430)527:0.17835745,(O6C65:0.16117497,O6C76:0.12437150)337:0.01157914)245:0.02618217)225:0.02438841)309:0.02736355,(O6C75:0.09098765,(OR6C1:0.15961088,OR6C3:0.12528803)528:0.09277735)372:0.03089089)453:0.07126178)526:0.33993978)321:0.10818214)152:0.04051139)310:0.05887108)407:0.06660607)412:0.09624557,((O2AT4:0.47573296,(((O56A3:0.07794031,(O56A1:0.08642389,(O56A4:0.03955636,O56A5:0.09055217)526:0.08220261)417:0.04870690)528:0.28345392,(O56B4:0.17762556,(O56B1:0.17249306,O56B2:0.15842700)322:0.06860075)455:0.19158241)527:0.40765570,((O52I1:0.04051944,O52I2:0.00538885)528:0.65150129,(O52W1:0.60292702,((O52L1:0.03457066,O52L2:0.04090765)528:0.25427489,((O52P1:0.33424516,(O52M1:0.39800768,((O52K1:0.03061273,O52K2:0.03207580)528:0.33889952,((O51E1:0.27914823,(O51D1:0.34536912,O51E2:0.25727868)311:0.06354017)484:0.09370833,(((O51L1:0.35591099,(O51Q1:0.47778929,((O51T1:0.45217820,(O51F1:0.24755604,O51F2:0.20263096)512:0.11938910)502:0.09263756,(O51M1:0.40979887,(O51V1:0.32715887,(O51B5:0.13509157,(O51B2:0.21191443,(O51B6:0.14795054,O51B4:0.23294610)254:0.01924624)507:0.07826682)528:0.27034270)465:0.10239468)292:0.07496636)186:0.03514969)63:0.02525828)36:0.02171722,(O51H1:0.46999843,(O51G2:0.21228251,(O51G1:0.31124159,(O51S1:0.79155871,(O51A7:0.17908867,(O51A2:0.02769260,O51A4:0.00435727)528:0.13769407)528:0.20696335)132:0.05938247)77:0.03774088)151:0.05516526)130:0.07209214)30:0.03065377,(O51J1:0.82855893,(O51I2:0.25241409,O51I1:0.41342648)338:0.09833191)154:0.06782835)448:0.12106381)495:0.15178630)246:0.03998361)282:0.07745381)150:0.03275195,((O52R1:0.35562515,((O52A5:0.11354259,(O52A4:0.32579212,O52A1:0.12390038)409:0.04018407)525:0.36344430,(O52N4:0.09979912,(O52N1:0.13118294,(O52N5:0.19745769,O52N2:0.11267771)267:0.01862088)417:0.07199124)527:0.32019076)205:0.05060995)184:0.06492732,((O52D1:0.23839950,(O52Z1:0.25794812,(O52B6:0.30705119,(O52B2:0.19518549,(O52B4:0.37227881,O52H1:0.35467459)279:0.02848933)426:0.08161683)391:0.07325441)456:0.09735463)468:0.11666879,(O52J3:0.32517311,((O52E6:0.06541550,O52E8:0.03387678)527:0.19197669,((O52E2:0.16347185,O52E1:0.16472326)386:0.06366267,(O52E4:0.17505105,O52E5:0.21910004)151:0.01778305)266:0.04047268)521:0.18267066)313:0.08399305)183:0.08799219)145:0.04975067)185:0.07254869)187:0.11663623)203:0.04207568)445:0.21725745)490:0.48735107)381:0.37644160,(OR6Q1:0.50165334,((OR6B1:0.34253965,(OR6A2:0.28933146,(OR6P1:0.30789666,OR6Y1:0.30320067)493:0.07211352)265:0.06843603)130:0.02158307,(OR6B2:0.02293342,OR6B3:0.01090039)528:0.34835690)481:0.13309007)283:0.07411793)54:0.04910081)21:0.02360604,((OR6K2:0.25452418,(OR6K3:0.20058406,OR6K6:0.24533316)333:0.06521982)525:0.27163130,(OR6N1:0.18708465,OR6N2:0.24158787)384:0.06267812)518:0.25835843)86:0.04797278,(O11L1:0.46325093,(O11A1:0.60798847,(O11G2:0.23897415,((O11H7:0.23724776,O11H4:0.17882017)484:0.10432087,(O11H6:0.18321306,(O11H2:0.00675737,(O11HC:0.00751767,O11H1:0.00386050)313:0.00863800)528:0.26436588)212:0.05396679)228:0.04991910)513:0.26790252)194:0.05661486)157:0.06416020)114:0.04258700,(((((OR1L4:0.03025768,OR1L6:0.02220177)528:0.16412668,(OR1L8:0.25306895,(OR1L1:0.10086344,OR1L3:0.13823152)321:0.03907070)480:0.08984169)523:0.16090467,(OR1Q1:0.50692018,(((OR1C1:0.34688109,OR1FC:0.30937931)220:0.05426871,((OR1I1:0.40325679,(OR1G1:0.29516172,((OR7D2:0.23251827,OR7D4:0.17752210)250:0.03316742,((OR7G3:0.16525508,(OR7G1:0.14100607,OR7G2:0.12903742)527:0.09259377)524:0.11355827,((OR7A5:0.11113833,(OR7AA:0.05233806,(OR7A2:0.12330251,OR7AH:0.08925401)297:0.02153841)316:0.02551384)526:0.10979597,(O7E24:0.32070405,(OR7C1:0.09610789,OR7C2:0.10723834)527:0.07125741)201:0.03542944)62:0.01693758)125:0.03492597)525:0.19654366)293:0.03951651)161:0.04561186,(((OR1E3:0.17706291,(OR1E1:0.01587915,OR1E2:0.01957245)524:0.05240829)526:0.16714586,(OR1J4:0.10036521,(OR1J1:0.18655342,OR1J2:0.14173635)258:0.02463676)528:0.20423073)462:0.11013657,((OR1P1:0.32548467,(OR1S1:0.01520586,OR1S2:0.06312825)528:0.35888646)137:0.04930195,(OR1M1:0.39596788,(OR1N1:0.28403275,(OR1N2:0.27886744,(OR1D2:0.06259567,(OR1D4:0.00368093,OR1D5:0.00740236)527:0.10891270)527:0.36760901)266:0.09048599)141:0.05296925)27:0.01003120)68:0.03488721)40:0.02127798)85:0.03046412)201:0.05374607,((OR1A1:0.04170089,OR1A2:0.11561691)528:0.40517288,(OR1F1:0.06945763,OR1F2:0.13677190)528:0.13596883)188:0.05175716)160:0.04417833)127:0.04845511)127:0.02194448,(OR1K1:0.32935923,OR1B1:0.63732624)364:0.09349102)424:0.20685902,(O10AD:0.80964215,((((OR5V1:0.41490537,((OR3A4:0.21175866,(OR3A3:0.01592502,(OR3A1:0.13202229,OR3A2:0.03206598)304:0.05378346)384:0.06945503)528:0.43059872,(O13A1:0.46274306,O13G1:0.33795547)515:0.24855275)297:0.10671216)82:0.03291715,((O12D2:0.20386874,O12D3:0.21140263)526:0.43270158,(((OR4E2:0.27850239,OR4E1:0.22930089)513:0.11544941,(((OR4S1:0.35806379,(OR4P4:0.38441624,OR4S2:0.23327201)358:0.06101265)250:0.02217177,(OR4B1:0.29984400,((OR4X1:0.34115360,OR4X2:0.21453397)441:0.10810427,((OR4CB:0.13647402,OR4CG:0.27902182)527:0.24757021,((O4A15:0.22364118,((O4A47:0.02821760,OR4A4:0.03675675)528:0.20781333,(OR4A5:0.11857305,(O4A16:0.19590565,OR4A8:0.14022294)333:0.01063093)519:0.08883751)433:0.04877293)527:0.12033424,((OR4C6:0.32436594,((OR4CC:0.25741486,(O4C46:0.08027261,OR4CD:0.09822969)525:0.06519632)343:0.05936773,(OR4C3:0.28085409,OR4CF:0.23973145)298:0.06141376)243:0.02769955)129:0.02437233,(OR4C5:0.21616140,O4C45:0.36170985)180:0.03505882)398:0.05918616)492:0.07756824)278:0.02495711)175:0.02925291)214:0.03088657)484:0.14469685,(((OR4D5:0.20692623,((OR4D1:0.09496592,OR4D2:0.11483872)527:0.14017840,(OR4D6:0.24345835,(OR4DA:0.12389027,(OR4D9:0.07980898,OR4DB:0.09400906)243:0.02161558)527:0.13093884)411:0.04386624)524:0.12379169)510:0.09795820,(OR4Q2:0.25876314,((OR4M1:0.00388312,OR4M2:0.05346349)528:0.26059251,(OR4N5:0.04114858,(OR4N2:0.04977590,OR4N4:0.07196393)493:0.05396423)525:0.26597254)513:0.13547276)369:0.06600134)135:0.04996008,(OR4Q3:0.32862173,(((OR4F5:0.00728760,(O4F17:0.00005940,OR4F4:0.00369275)426:0.00010028)528:0.26040855,(OR4F6:0.15892736,(OR4FL:0.16558567,(O4F15:0.14110661,(O4F21:0.00737060,OR4F3:0.00010029)528:0.20338176)470:0.06429711)457:0.02526296)528:0.21857609)498:0.09925823,(OR4L1:0.35120477,(OR4KH:0.32732137,((OR4K1:0.27999546,((OR4K5:0.25820878,OR4KF:0.13123237)431:0.09364551,(OR4KE:0.17334326,OR4KD:0.26132273)126:0.03290503)81:0.02229648)53:0.03271907,(OR4K3:0.11666076,OR4K2:0.29576280)500:0.10318004)46:0.02214753)120:0.03731144)171:0.03066885)472:0.18540706)213:0.02507220)270:0.05193450)211:0.05619128)406:0.19594360,((((O10G8:0.09011805,O10G7:0.02185511)433:0.01292660,(O10G9:0.02025883,O10G4:0.02879403)314:0.01853939)526:0.30559507,(O10S1:0.38416853,(O10G6:0.33035826,(O10G2:0.11003522,O10G3:0.17375191)527:0.14855654)340:0.06272659)407:0.08738229)379:0.08925510,(SMOCORR:8.25390223,(O10D3:0.17867904,O10D4:0.18255453)524:0.22455284)103:0.12494081)107:0.15209110)111:0.06350737)119:0.05641141)41:0.05027849,(OR8S1:0.69703323,(OR9K2:0.56069032,((OR5J2:0.42987900,(((((OR5G3:0.42426561,(((OR5L1:0.03819166,OR5L2:0.04633713)528:0.25488928,((OR5DG:0.19271298,OR5DI:0.06230506)500:0.05941198,(OR5DD:0.30255010,OR5DE:0.19207340)475:0.03896574)525:0.13052301)525:0.14563345,(O5AK2:0.10603934,O5AK3:0.06224081)527:0.39683417)94:0.02831476)74:0.02547858,((O5AS1:0.29210053,OR5I1:0.31451283)446:0.09585397,(O5AR1:0.30923817,(OR5C1:0.37428303,OR5W2:0.31950508)279:0.06169254)193:0.04085762)191:0.06037105)13:0.01330810,(OR5T2:0.11600260,(OR5T1:0.05162527,OR5T3:0.07762986)329:0.03847677)527:0.30110235)23:0.02959916,(O5AP2:0.33374829,(((O5AU1:0.42142793,((OR5BL:0.16758162,(OR5BC:0.12166277,(OR5B2:0.11639160,(OR5B3:0.11689483,OR5BH:0.21458489)244:0.01719469)516:0.08781125)462:0.07831584)528:0.16627999,(OR9I1:0.27696737,(OR9Q1:0.22653702,OR9Q2:0.08187409)527:0.19276557)527:0.22634274)146:0.06467682)31:0.01934318,(OR5P2:0.24724476,OR5P3:0.21558456)520:0.23667307)16:0.01348899,((OR5A1:0.21293767,(O5AN1:0.38318450,OR5A2:0.20363730)318:0.04502229)528:0.18889644,(OR9G4:0.23846826,(OR9G1:0.03464276,OR9G9:0.02632629)528:0.46259956)519:0.16397525)276:0.07666829)67:0.04037087)19:0.02390650)13:0.01258884,(((OR5K1:0.00010024,OR5K2:0.04963537)528:0.13122070,(OR5K4:0.14693330,OR5K3:0.12266777)326:0.02627090)527:0.27203670,((OR5H2:0.08552830,((O5H15:0.03730634,OR5H1:0.01049412)528:0.04692560,(OR5H6:0.10673488,O5H14:0.03870258)350:0.04444476)524:0.07528065)526:0.14152828,(O5AC1:0.17522711,O5AC2:0.14258732)502:0.09383578)515:0.16062161)508:0.22213344)28:0.03190375)57:0.03563547,(((OR8D4:0.23512182,(OR8A1:0.20725967,((OR8D2:0.19214697,OR8D1:0.17050417)496:0.10254560,((OR8G2:0.13174627,(OR8G1:0.06106062,OR8G5:0.07315591)528:0.06433806)528:0.11154547,((OR8B8:0.05517645,OR8BC:0.14969923)528:0.12350980,(OR8B4:0.24466500,(OR8B2:0.01490337,OR8B3:0.00651951)528:0.10968863)281:0.02635560)528:0.14017413)408:0.04315888)272:0.03058988)387:0.04720932)524:0.21056926,((O5AL1:0.37564614,(((OR8J2:0.09499116,(OR8J1:0.05942997,OR8J3:0.04322722)528:0.08808189)528:0.26377455,(OR8K5:0.18916908,(OR8K1:0.24684571,OR8K3:0.13751518)286:0.01782843)526:0.12939511)479:0.10337519,(OR5R1:0.16824972,(OR8U9:0.03941222,(OR8U1:0.00010027,OR8U8:0.04069016)500:0.02232933)528:0.19109436)449:0.04146006)274:0.04651473)474:0.08471862,((OR5M8:0.16262487,(OR5M3:0.10497299,OR5M9:0.16143299)527:0.15267105)524:0.12242104,(OR5MB:0.23057478,(OR5M1:0.02444686,OR5MA:0.01232744)520:0.16871398)349:0.06457304)513:0.18817207)352:0.05203952)98:0.02892775,(OR5F1:0.32348888,(OR8I2:0.28978333,(OR8H1:0.05526258,(OR8H2:0.03674624,OR8H3:0.02808093)453:0.03305279)526:0.27440790)479:0.12664068)296:0.06957865)129:0.03725883)156:0.04407283)360:0.08318120)240:0.06299738)2:0.03126685,((O14CZ:0.45485549,(O14I1:0.41588493,((O14AG:0.26441725,(O14K1:0.39090919,O14A2:0.30496416)422:0.08564809)189:0.04372963,(O14L1:0.39929090,O14J1:0.43131416)107:0.02620501)423:0.09307289)355:0.06795637)492:0.34886046,(((((O2A25:0.08936165,(OR2A4:0.00746938,OR2A7:0.00010024)528:0.13565836)527:0.13073987,(OR2A1:0.06302432,(OR2A5:0.06136082,(O2A12:0.19724371,(OR2A2:0.18729740,O2A14:0.09086099)375:0.02883795)272:0.02908549)435:0.09478735)291:0.06710881)517:0.31918928,(OR2D2:0.26964185,OR2D3:0.23050777)525:0.25311526)131:0.03405089,(((O13J1:0.48875745,((O13F1:0.26114228,OR2K2:0.18405326)514:0.09409767,(O13D1:0.23997453,((O13C9:0.04679608,(O13C2:0.00785184,O13C5:0.05802279)493:0.04275892)528:0.16283467,((O13C3:0.06340828,O13C4:0.08661954)525:0.16078682,(O13C8:0.21484740,(OR2S1:0.10289252,YI035:0.11015182)527:0.07588544)493:0.05886765)449:0.05942395)518:0.10419433)456:0.04205681)320:0.05106001)509:0.13314957,(O13H1:0.54768387,(OR2F1:0.02634374,OR2F2:0.05572079)526:0.40324358)140:0.01633953)262:0.07416120,(((OR2BB:0.33874438,(OR2B3:0.20765339,(OR2B2:0.07411564,OR2B6:0.11986748)446:0.05194237)522:0.11676571)231:0.04250058,((OR2G3:0.23249315,(OR2C1:0.30263317,(OR2H1:0.07255073,OR2H2:0.04871166)527:0.27508056)269:0.05310162)93:0.02656058,(((OR2C3:0.32708298,(OR2G2:0.19353682,OR2G6:0.25498118)258:0.03316843)287:0.05462980,(OR2B8:0.23449840,(OR2Y1:0.24900229,OR2I1:0.46796757)490:0.09883809)306:0.04156739)92:0.02923950,((OR2J3:0.03250883,(OR2J1:0.02645226,OR2J2:0.03735169)525:0.03125033)526:0.30531500,(OR2W6:0.15187559,(OR2W3:0.24639292,(OR2W5:0.90964533,OR2W1:0.28304461)142:0.05261698)141:0.04595802)442:0.11958985)113:0.03216793)143:0.03336286)322:0.07756981)494:0.26089412,(((O2AJ1:0.34577250,(O2AK2:0.38897809,(OR2LD:0.19820522,((OR2L3:0.01615597,OR2L8:0.02503564)522:0.05137135,(OR2L2:0.06886593,OR2L5:0.03607561)450:0.02845762)468:0.06872411)528:0.15959833)399:0.13482602)269:0.07876737,(O2AE1:0.43338827,(((O2T10:0.19457897,((O2T34:0.01668691,OR2T3:0.00519308)528:0.27076523,(OR2T4:0.04446758,(O2T29:0.00006644,OR2T5:0.00006644)528:0.03902466)527:0.07735520)343:0.02590337)528:0.13743688,((O2T11:0.17362371,(O2T35:0.00010025,OR2T2:0.00357241)528:0.11283505)500:0.06803459,((O2T27:0.00252131,OR2T7:0.01919912)528:0.17286188,(OR2T1:0.08342121,OR2T6:0.21824539)509:0.05532386)522:0.08078120)481:0.05275184)518:0.12277953,((O2T12:0.03066312,(OR2T8:0.01527358,O2T33:0.00285378)376:0.01067946)523:0.29825257,((OR2V1:0.07797409,OR2V2:0.06724560)528:0.26487755,(OR2M4:0.18136909,(OR2M2:0.04042214,(OR2M3:0.03560360,(OR2M7:0.03595453,OR2M5:0.02928692)282:0.01462149)282:0.00010052)515:0.13739952)514:0.19064487)396:0.06947466)362:0.06961765)313:0.04041921)150:0.02404803)148:0.03471308,(OR2Z1:0.40543383,(O2AG1:0.07776217,O2AG2:0.07709561)528:0.34179358)385:0.12684866)460:0.22139256)108:0.03919353)70:0.03273398)155:0.07784971,((((O10A3:0.03301924,O10A6:0.12491807)528:0.35271005,(O10A7:0.30248629,(O10A4:0.13359482,(O10A2:0.02483799,O10A5:0.00890134)528:0.13200718)519:0.12573243)413:0.06277904)453:0.08912184,(O10AG:0.50653368,(O10P1:0.47909236,O10C1:0.39681355)377:0.08839690)183:0.03384133)446:0.11505277,((((O10R2:0.31866789,O10T2:0.29972616)338:0.07226316,(((O10J3:0.15732401,O10J4:0.16727019)433:0.06248500,(O10J1:0.13059586,(O10J6:0.16548280,O10J5:0.16211300)522:0.08007579)496:0.07067617)528:0.27784179,(O10Z1:0.36759020,(O10X1:0.42425930,((O10H3:0.05490820,O10H4:0.03103943)528:0.16445744,(O10H2:0.04481897,(O10H1:0.00779575,O10H5:0.02645821)508:0.04584219)407:0.05550390)527:0.31439098)485:0.14419762)179:0.06545605)161:0.04672169)155:0.02105793,(O10K1:0.07604768,O10K2:0.07749449)528:0.29603615)447:0.09734919,(O10AC:0.74485425,(O10W1:0.44601258,(O10Q1:0.26006312,O10V1:0.38155924)398:0.07208596)410:0.11463345)419:0.09617007)453:0.05847508)320:0.05284887)45:0.03467223)17:0.03039833)5:0.02019553)9:0.02367135)60:0.05511810)125:0.46186219)47:0.00010205)79:0.14006365)30:0.15428999)5:0.10159816)1:0.12415909,((GPR21:0.29088060,GPR52:0.42373527)527:1.41457866,(GPR27:0.73118926,(GPR85:0.44357459,GP173:0.57629424)411:0.23706965)527:1.14524848)154:0.15879575)2:0.07628133)1:0.04317877,((GP161:1.71883362,((OPSX:1.19295185,(OPN3:0.92326543,(OPSD:0.54719709,(OPSB:0.78920374,(OPSG:0.00016027,OPSR:0.33067864)528:0.55161056)240:0.08033507)521:0.57110379)287:0.21902819)223:0.18931094,(OPN5:1.34645319,(OPN4:0.76273379,RGR:2.02660142)107:0.04669771)207:0.25546108)252:0.14615208)82:0.20062374,((GP101:1.50007420,GP135:1.12439383)96:0.18222735,(((GPBAR:2.35144800,GPR75:3.00933896)80:0.45573598,(GPR88:4.06767612,(GPR61:0.78784097,GPR62:1.48163159)389:0.29595743)162:0.13328557)28:0.11236986,(GPR26:0.68531544,GPR78:0.52843281)523:1.05586598)19:0.19251054)5:0.14382804)5:0.21319282)0:0.01196499,(MTR1L:0.54320158,(MTR1A:0.16547799,MTR1B:0.41124720)447:0.22533596)523:1.12952539)0:0.11375766)2:0.11734262)0:0.08936300,((CCKAR:0.44967219,GASR:0.38112906)527:0.81495246,(((NK2R:0.55292524,(NK1R:0.25051211,NK3R:0.36711309)362:0.18718956)527:0.72290907,(GPR83:0.86101265,(PRLHR:0.66081673,(NPY2R:0.84022323,((GPR84:2.19126432,NPY5R:0.84259315)124:0.54719404,(NPY6R:0.66132459,(NPY4R:0.56230654,NPY1R:0.44526693)284:0.09445144)511:0.34904821)65:0.15768433)32:0.19647334)15:0.14266869)16:0.14173530)8:0.12188836,((OX1R:0.31608810,OX2R:0.40069531)528:0.69664143,(QRFPR:1.32734733,(NPFF1:0.36744201,NPFF2:0.37578210)527:0.41385752)101:0.18260208)53:0.15783734)8:0.09973569)15:0.15256850)1:0.09683210)5:0.14703937)2:0.06679329,((((SSR1:0.35450392,SSR4:0.29885097)500:0.20660256,(SSR2:0.40969400,(SSR3:0.35759459,SSR5:0.35839496)385:0.08847324)478:0.13231512)433:0.16853373,((NPBW1:0.35898189,NPBW2:0.33096958)526:0.53606976,(OPRX:0.39959099,(OPRK:0.22077531,(OPRM:0.22459890,OPRD:0.27893634)322:0.06180458)348:0.07756426)527:0.42068874)242:0.10406618)364:0.18714877,(MCHR1:0.86135556,MCHR2:1.03965447)313:0.22600571)221:0.23670460)12:0.09664796,((((((CML1:0.78455779,GPR1:0.86721001)154:0.12542570,((FPR1:0.32293019,(FPR3:0.30582928,FPR2:0.06652860)381:0.17883527)526:0.58271678,(GPC39:0.62084354,GPR32:0.20277888)528:0.79522839)365:0.22901472)100:0.07704819,(C3AR:0.94003445,(C5ARL:0.88128172,C5AR:0.62630396)379:0.22921437)303:0.20906712)95:0.04077107,(PD2R2:0.98166955,GPR33:1.05057882)215:0.25559273)219:0.16972665,(GP152:1.74628875,((MRGX2:0.32812276,(MRGX1:0.10527223,(MRGX4:0.21115017,MRGX3:0.19040350)383:0.10639496)343:0.13821241)527:0.53026757,(MRGRF:1.47998623,((MAS1L:1.31741841,(MRGRD:0.91849203,MAS:1.09249558)250:0.04493014)217:0.14927824,(MRGRE:1.43334502,MRGRG:1.12684258)223:0.23975507)110:0.10168608)165:0.10860058)485:0.47421513)265:0.19400846)106:0.09496383,(LT4R2:0.90156940,LT4R1:0.52665302)519:0.78635139)121:0.12665396)22:0.10415732)24:0.06626834,(((AGTR2:1.01848712,(RL3R1:0.49119911,RL3R2:1.10541981)522:0.60064846)100:0.16518900,((GPR25:0.72174178,GPR15:0.82891531)488:0.39895650,(APJ:1.17537859,AGTR1:0.83221299)288:0.16001890)193:0.15231738)29:0.06443977,((GP182:0.93299581,CXCR7:0.62023236)511:0.47063156,(XCR1:1.08975223,((CX3C1:0.74226212,(CCR8:0.60716718,(CCR4:0.35998363,((CCR2:0.11602761,CCR5:0.11273060)513:0.15841358,(CCRL2:0.83373513,(CCR1:0.37818893,CCR3:0.33651547)498:0.10035765)229:0.05066132)421:0.15677255)381:0.13979837)268:0.09867924)434:0.21033423,((((BKRB2:0.69236802,BKRB1:0.75154470)525:0.61928859,(CXCR1:0.32186237,CXCR2:0.20328343)522:0.44727624)74:0.05130838,(CCBP2:0.67658967,CXCR3:0.63702752)140:0.04222490)237:0.32132754,(((CCR6:0.49104261,CCR7:0.67863318)187:0.07453047,(CXCR6:0.75977138,(CCRL1:0.81877140,CCR9:0.54822254)282:0.07416001)212:0.07230916)270:0.18656680,((CCR10:0.81368090,DUFFY:4.11797464)194:0.25768114,(CXCR4:0.67797036,CXCR5:0.91838116)265:0.12707533)122:0.14068194)103:0.10274384)102:0.08453444)92:0.12563494)106:0.09821455)86:0.11923575)40:0.07001728)148:0.22669644,((GPR18:1.25360425,(GPR17:0.76278102,(CLTR2:0.81430521,CLTR1:0.72718658)300:0.17887191)118:0.10303284)76:0.07919509,(P2Y11:2.26702642,((P2RY1:0.56754740,(SUCR1:1.17134114,OXGR1:0.76655102)310:0.21939757)246:0.16264818,(P2RY6:0.81865557,(P2RY2:0.28481692,P2RY4:0.36981318)507:0.34230274)393:0.16345582)228:0.08926403)148:0.18532818)53:0.09308063)12:0.04504950)5:0.05271028,((FFAR1:1.32806900,(FFAR2:0.87454905,(FFAR3:0.06396306,GPR42:0.10702251)526:0.44846867)371:0.16375944)520:0.74673897,(PAR4:1.12137634,(PAR3:1.21672092,(PAR1:0.81460978,(P2RY8:0.89672528,PAR2:0.67440068)181:0.06286216)242:0.10720468)110:0.11335789)82:0.07590375)71:0.16295801)14:0.06315809,((PTAFR:1.34941344,((OGR1:0.67668403,GPR4:0.47443588)511:0.32805902,(PSYR:0.84776158,GP132:1.07313098)366:0.25035325)487:0.44403011)185:0.13268503,((LPAR5:1.21733163,(GPR55:1.06256939,GPR35:0.86091253)447:0.29984253)157:0.05494718,((GP141:7.26408766,(LPAR4:0.34150792,LPAR6:0.51924234)510:0.23450361)102:0.09920980,(GP174:0.45943384,P2Y10:0.58249677)528:0.60343643)140:0.19629833)49:0.09115563)15:0.05924315)101:0.13148327)509:0.52414896)495:0.22840404)528:0.37321437);
